# Male survival disadvantage in pulmonary hypertension: independent of aetiology, age, disease severity, comorbidities and treatment

**DOI:** 10.1016/j.ebiom.2025.106063

**Published:** 2025-12-16

**Authors:** Athiththan Yogeswaran, Jeffrey S. Annis, Meike Fünderich, Jochen Wilhelm, David G. Kiely, Luke Howard, Allan Lawrie, Martin R. Wilkins, Aparna Balasubramanian, Paul M. Hassoun, Ziad Konswa, Christina A. Eichstaedt, Ekkehard Grünig, Andrew J. Sweatt, Roham T. Zamanian, Gabor Kovacs, Horst Olschewski, Mona Lichtblau, Silvia Ulrich, Thenappan Thenappan, Imad Al Ghouleh, Stephen Y. Chan, Jean Elwing, Arun Jose, John Cannon, Joanna Pepke-Zaba, Robert Frantz, Yuriy Sirenko, Olena Torbas, Sandeep Sahay, Zhenguo Zhai, Zhu Zhang, Alexandra Arvanitaki, George Giannakoulas, Marlize Frauendorf, Paul G. Williams, Keiichiro Kuronuma, Hiromi Matsubara, Stefano Ghio, Laura Scelsi, Hanni Sabbour, Khaled Saleh, Anastasia Anthi, Effrosyni Dima, Raphael W. Majeed, Hossein-Ardeschir Ghofrani, Friedrich Grimminger, Khodr Tello, Hector R. Cajigas, Evan Brittain, Werner Seeger, The PVRI Consortium, James Anderson, Tobiah Antoine, Felix Ballmann, Harm Jan Bogaard, Victoria Damonte, Nathan Dwyer, Diego Echazarreta, Kai Förster Lars Harbaum, Melanie Heberling, Anne Hilgendorff, Ernesto Junaeda, Ingrid King, Hans Klose, Philipp Krieb, Edmund Lau, Melanie Lavender, Kurt Marquardt, Mauricio Orozco-Levi, Karen Osborn, Stephan Rosenkranz, Siva Sivakumaran, Ioan Tilea, Andrea Varga, Helen M. Whitford, Christoph B. Wiedenroth

**Affiliations:** a Department of Internal Medicine, Universities of Giessen and Marburg Lung Center (UGMLC), Member of the German Center for Lung Research (DZL), Giessen, Germany; b Institute for Lung Health (ILH), Cardio-Pulmonary Institute (CPI), Giessen, Germany; c Vanderbilt University Medical Center, Nashville, USA; d Sheffield Pulmonary Vascular Disease Unit, Royal Hallamshire Hospital, University of Sheffield and National Institute for Health and Care Research Sheffield Biomedical Research Centre, UK; e National Heart and Lung Institute, Imperial College London, London, UK; f Division of Pulmonary and Critical Care Medicine, Department of Medicine, Johns Hopkins University School of Medicine, Baltimore, USA; g Center for Pulmonary Hypertension, Thoraxklinik Heidelberg gGmbH at Heidelberg University Hospital and Translational Lung Research Center Heidelberg (TLRC), German Center for Lung Research (DZL), Heidelberg, Germany; h Laboratory for Molecular Genetic Diagnostics, Institute of Human Genetics, Heidelberg University, Heidelberg, Germany; i Division of Pulmonary, Allergy, and Critical Care, and Vera Moulton Wall Center for Pulmonary Vascular Disease, Stanford University, USA; j Division of Respiratory Medicine, Lung Research Cluster, Medical University of Graz, Graz, Austria; k Charité-Universitätsmedizin Berlin, corporate member of Freie Universität Berlin and Humboldt-Universität zu Berlin, Department of Infectious Diseases and Respiratory Medicine, Berlin, Germany; l Division of Pulmonology, University Hospital Zurich, Switzerland; m University of Minnesota, USA; n Cardiovascular Research Center, Brown University Health Cardiovascular Institute, Department of Medicine, The Warren Alpert Medical School of Brown University, USA; o University of Pittsburgh, USA; p University of Cincinnati, USA; q Royal Papworth Hospital Cambrigde, UK; r Division of Pulmonary and Critical Care Medicine, Mayo Clinic, Rochester, USA; s Strazhensku Cardiology Institute Kiev, Ukraine; t The Methodist, Houston, USA; u Department of Respiratory and Critical Care Medicine, China-Japan Friendship Hospital, Beijing, China; v 1th Department of Cardiology, Aristotle University of Thessaloniki, Greece; w Milpark Hospital, Johannesburg, ZA, South Africa; x Pulmonary Hypertension Center, Department of Cardiology, NHO Okayama Medical Center, Okayama, Japan; y University di Pavia, Italy; z Cleveland Clinic Abu Dhabi, United Arab Emirates; aa Evangelismos Hospital Athens, Greece; ab Institute of Medical Informatics, RWTH Aachen University, Aachen, Germany

**Keywords:** Pulmonary hypertension, Meta-registry, Sex differences

## Abstract

**Background:**

Sex-based differences in morbidity and mortality in pulmonary hypertension (PH) are underexplored, yet understanding these differences is vital for improving clinical management. This study investigates the influence of sex on survival of patients with PH in dependency of various disease conditions.

**Methods:**

The PVRI GoDeep meta-registry integrates data from international PH registries, of which we analysed 21,123 incident hemodynamically fully characterised patients with PH. Survival analyses employed Kaplan–Meier and Cox proportional hazards models, adjusted for confounders and subjected to sensitivity analyses.

**Findings:**

Male patients consistently showed significantly higher mortality than females across the overall PH population (hazard ratio 1.36 [1.23, 1.50] after adjustment) and within PAH and non-PAH groups. These sex differences in survival persisted regardless of P(A)H severities, age and obesity, cardiovascular diseases, and PAH-specific therapies. The male survival disadvantage was noted across low-, intermediate-, and high-risk groups of the ESC/ERS 2022, REVEAL lite 2, and COMPERA 4-strata scores, but not the REVEAL 2.0 risk score, which incorporates male sex as non-modifiable factor. Stratification by race revealed that male sex was associated with worse survival in White patients, but not in Black or Asian patients with PH.

**Interpretation:**

Male patients with PH exhibit significantly higher mortality risks than females across both PAH and non-PAH PH groups. This disparity persists regardless of PH severity, underlying cause, age, obesity, comorbidities, or treatment status, though race might modify the observed risk difference. These insights provide new avenues for investigating underlying mechanisms and suggest including male sex as an independent factor in clinical risk assessment tools.

**Funding:**

This work is funded by the Pulmonary Vascular Research Institute (PVRI) and the 10.13039/100016421Cardiovascular Medical Research and Education Fund (CMREF), 10.13039/100000002NIH.

**Research in context**

**Evidence before this study**

Previous research on sex-based differences in pulmonary hypertension (PH) outcomes has yielded conflicting evidence, constrained by methodological limitations such as narrow populations, small cohorts, or inadequately adjustment for disease severity, treatment variables, and comorbidities. A global, multi-subtype analysis of survival disparities across sex remained absent.

**Added value of this study**

This investigation leverages data from 21,123 incident patients with PH in the PVRI GoDeep meta-registry—the largest hemodynamically characterised cohort to date—to provide a comprehensive assessment of sex-based mortality trends. Key advances include demonstrating male sex as an independent predictor of higher mortality across PAH and non-PAH subtypes, persisting after rigorous adjustment for age, obesity, comorbidities, treatment status, and disease severity. We also identified potential race-specific variations, with the survival disadvantage for males definitely proven in Whites, but possibly absent in Black and Asian subgroups. Finally, we validated male sex as an additional prognostic marker across multiple risk frameworks (e.g., ESC/ERS risk score), except for REVEAL 2.0, which already incorporates sex in its algorithm.

**Implications of all the available evidence**

These findings support the formal inclusion of male sex in PH risk stratification tools, and strongly underscore the need for further mechanistic research to delineate the biological drivers and sociocultural factors underlying sex–race interdependence in PH-related mortality. They also highlight the importance of developing sex- and race-sensitive management strategies to address disparities in PH outcomes.

## Introduction

Sex-based differences in prevalence, phenotype, treatment response, and survival are evident in several cardiovascular diseases including heart failure.^1^, ^2^, ^3^, ^4^ In pulmonary arterial hypertension (PAH), females are diagnosed more frequently, aligning with the “classic” phenotype of young idiopathic PAH (IPAH) patients. However, they exhibit better survival rates than male patients with PAH, a phenomenon termed “sex paradox”.^5^, ^6^, ^7^, ^8^ A pooled analysis of prospective randomised controlled trials provided evidence for complex sex-dependent interactions involving age, body size, exercise capacity, and pulmonary haemodynamics.^9^ Moreover, experimental and clinical studies indicate that females display superior load-independent contractility of the right ventricle compared to males.^10^, ^11^, ^12^, ^13^, ^14^

Registry data suggest a trend towards “atypical” PAH, with increasing diagnoses among older and obese men.^5^^,^^6^^,^^15^ Additionally, it remains uncertain whether the female survival advantage is present in also non-PAH pulmonary hypertension (PH) groups, which include significantly larger patient populations than PAH.^16^, ^17^, ^18^ Further open questions include the dependency of sex-related survival disparities in P(A)H on disease severity, age, comorbidities, race and the response to treatment with PAH-targeting drugs. To investigate these questions, we analysed sex-dependent features in the large Pulmonary Vascular Research Institute (PVRI) GoDeep meta-registry of over 33,000 patients with invasively diagnosed PH.

## Methods

### PVRI GoDeep registry

The PVRI GoDeep is a global meta-registry that integrates both retrospective and prospective data from PH referral centres worldwide, each of which systematically records PH patient cohorts.^19^^,^^20^ Patient data undergo harmonisation, validation and anonymisation (k-anonymisation) prior to inclusion, adhering to diagnostic criteria and classification guidelines.^21^, ^22^, ^23^ Specifically, GoDeep applies standardised terminology and coding systems (SNOMED-CT, LOINC) within a FHIR-based data model to harmonise datasets across international centres, ensuring interoperability and regulatory compliance. Data quality is safeguarded through multiple control steps: time-sequence checks confirm the correct chronological order of events, plausibility checks ensure that values are consistent with physiological norms and disease-specific characteristics, and cross-variable consistency is evaluated (e.g., between haemodynamic and clinical data). Furthermore, participating centres receive continuous feedback reports to identify, verify, and resolve potential discrepancies. This standardised approach ensures robust and reliable integration of data across all contributing registries.

As of April 2025, 26 centres have contributed a total of 33,362 patients to the meta-registry (Figure E1). Patients from the following PH centres were included in the analyses: Vanderbilt, Giessen, Sheffield, London, Baltimore, Heidelberg, Stanford, Graz, Zurich, Minnesota, Pittsburgh, Cincinnati, Cambridge, Rochester, Kiev, Houston, Beijing, Thessaloniki, Johannesburg, Okayama, Pavia, Abu Dhabi, and Athens. The University of Giessen/University Hospital Ethics Committee (AZ 30/19) and the responsible local ethic committees have approved the PVRI-GoDeep central data repository structure, including transfer of anonymised data from the local referral centres to this meta-registry (NCT05329714). All investigations were conducted in compliance with relevant ethical regulations. Written informed consent was obtained from all participants upon entering into the single registries of the local referral centres.

### Patient and data selection

Patients aged >18 years, incident cases, with complete haemodynamic data, reported sex (female or male), and without data inconsistencies were included (Figure E1). Baseline data were collected from −1 to 6 months surrounding the date of diagnosis and registry entry. PH diagnosis was based on right heart catheterisation (RHC) undertaken by the referral centres according to international guidelines at enrolment.^21^^,^^24^^,^^25^ Patients originated from Europe, the Americas, Africa, and Asia, with the vast majority (99%) diagnosed after 2000. The outcome variable was transplant-free survival, defined as the time from diagnosis to either death from any cause or lung/heart–lung transplantation, whichever occurred first. Patients were censored at the date of last follow-up if alive and without transplantation. Analyses were conducted for the entire PH cohort as well as for the following subgroups: PH-treated, PH-untreated, PAH, PAH-treated, PAH-untreated patients, and non-PAH patients with PH excluding unclassified patients and those with mixed diagnoses, PH Group 2, PH Group 3, PH Group 4, as well as PH mixed and undefined. Additional subgroup analyses were conducted for patients from centres reporting cardiovascular comorbidities, those reporting obesity, patients with available estimated glomerular filtration rate (eGFR), and patients with recorded race information. Cardiovascular comorbidities included arterial hypertension, valvular heart disease, coronary artery disease, left heart failure (heart failure with preserved ejection fraction, heart failure with reduced ejection fraction, heart failure with mildly reduced ejection fraction), cardiomyopathies, and atrial flutter/fibrillation.

### Statistics

Data were analysed using R version 4.3.3^26^ using the *survival* package version 3.5.8.^27^ Missing values were calculated from available data whenever feasible. Continuous variables were summarised in tables as median [Q1, Q3]. Analyses were first conducted using only patients with complete data, followed by sensitivity analyses that included imputed datasets.

Unadjusted survival by groups is shown by Kaplan–Meier estimates which are compared by log-rank tests. Multivariable Cox proportional hazards models were fitted using the *coxph* function, incorporating age at diagnosis, sex, centre, diagnosis decade, world health organisation (WHO) functional class (FC), body mass index (BMI), pulmonary vascular resistance (PVR) or PVR index, mean pulmonary artery pressure (mPAP) and PH-targeting treatment information. Age was included as a natural spline with 2 degrees of freedom and centre and diagnosis decade were added as strata. An additional analysis was conducted using the PVR index instead of PVR, limited to patients with available body surface area. Depending on the analysis, stratification was performed by PVR, risk score, race, or age group. Additional subgroup analyses were conducted for comorbidities. All Cox models were calculated using either unimputed data only or datasets including imputed data. Missing data were imputed using the *mice* package version 3.16.0, utilising information from all variables in the Cox PH model except race, risk score, and PH treatment information. The plausibility of the imputed data distribution was verified using diagnostic plots, confirming the independence of missingness on sex (Figure E2). The proportional hazards assumption was confirmed by diagnostic plots of Schoenfeld residuals, and influential data points were assessed with deviance residual plots. Statistical significance of the estimated effects on survival was determined via Type-III likelihood-ratio tests, while individual coefficient significance was derived from Wald z-tests. Sensitivity analyses included estimating Heller Explained Relative Risk and by adding and removing individual covariables and groups of covariables to the base and from the full Cox model with imputed data. Severe PH was defined as a PVR >5 Wood units (WU)^23^; using a linear regression model of the log PVR index against the log PVR, the corresponding cutoff for the PVR index was determent to be at >9.5 WU·m^2^. Chronic kidney disease was defined as eGFR <90 ml/min/1.73 m^2^.

### Role of funders

The funders had no involvement in the design, conduct, data collection, analysis, interpretation, or manuscript preparation.

## Results

### Baseline characteristics

As of April 2025, the PVRI GoDeep meta-registry included 29,838 incident patients with PH, of whom 21,123 patients met inclusion criteria (Figure E1). Among included patients, 25.4% had PAH (n = 5374), 9.2% Group 2 (n = 1950), 12.0% Group 3 (n = 2544), 12.0% Group 4 (n = 2528), 2.2% Group 5 (n = 455), and 39.2% had mixed or unclassified PH (n = 8272). The median age of the study population was 64 [52, 72] years. Most patients exhibited impaired exercise capacity, with 9470 (65% of those with available WHO FC) classified as WHO FC III and 1820 (13%) as class IV. Median 6-min walk distance (6MWD) was 290 [200, 380] metres. The median follow-up time was 2.2 [0.5, 5.2] years, and 5880 patients (28%) died within five years after diagnosis.

Pulmonary haemodynamics were severely impaired, with a median mean pulmonary artery pressure (mPAP) of 38 [29, 48] mmHg (Figure E3) and pulmonary vascular resistance (PVR) of 4.7 [2.6, 8.7] Wood units (WU). Further baseline characteristics are provided in Table 1.

**Table 1:** Baseline characteristics of patients stratified by sex and severity of pulmonary hypertension.

Sex	female		male		Overall
PVR	≤5 WU	>5 WU	≤5 WU	>5 WU	
N	5651	6179	5502	3791	21,123
**Age at diagnosis (years)**					
Median [Q1, Q3]	65 [54, 73]	62 [49, 72]	64 [54, 72]	65 [53, 73]	64 [52, 72]
Missing	0 (0%)	0 (0%)	0 (0%)	0 (0%)	0 (0%)
**WHO FC**					
I	122 (3.6%)	76 (1.6%)	185 (5.8%)	65 (2.1%)	448 (3.1%)
II	831 (25%)	651 (13%)	778 (25%)	490 (16%)	2750 (19%)
III	2152 (64%)	3362 (69%)	1893 (60%)	2063 (66%)	9470 (65%)
IV	242 (7.2%)	749 (15%)	312 (9.8%)	517 (16%)	1820 (13%)
Missing	2304 (41%)	1341 (22%)	2334 (42%)	656 (17%)	6635 (31%)
**BMI (kg/m^2^)**					
Median [Q1, Q3]	30 [25, 36]	27 [23, 32]	29 [25, 34]	27 [24, 31]	28 [24, 33]
Missing	307 (5.4%)	510 (8.3%)	201 (3.7%)	241 (6.4%)	1259 (6%)
**BSA (m^2^)**					
Median [Q1, Q3]	1.8 [1.7, 2]	1.7 [1.6, 1.9]	2.1 [1.9, 2.3]	2 [1.8, 2.1]	1.9 [1.7, 2.1]
Missing	83 (1.5%)	193 (3.1%)	55 (1%)	88 (2.3%)	419 (2%)
**Height (cm)**					
Median [Q1, Q3]	160 [160, 170]	160 [160, 170]	180 [170, 180]	170 [170, 180]	170 [160, 180]
Missing	327 (5.8%)	572 (9.3%)	218 (4%)	274 (7.2%)	1391 (6.6%)
**Weight (kg)**					
Median [Q1, Q3]	78 [65, 95]	70 [59, 84]	91 [78, 110]	81 [70, 94]	80 [67, 96]
Missing	363 (6.4%)	628 (10%)	229 (4.2%)	296 (7.8%)	1516 (7.2%)
**6MWD (m/6 min)**					
Median [Q1, Q3]	300 [210, 390]	270 [180, 360]	330 [240, 420]	280 [190, 390]	290 [200, 380]
Missing	3842 (68%)	2695 (44%)	4257 (77%)	1651 (44%)	12,445 (59%)
**BNP (pg/mL)**					
Median [Q1, Q3]	180 [64, 530]	260 [97, 570]	320 [100, 860]	290 [130, 640]	260 [93, 650]
Missing	2582 (46%)	2696 (44%)	2254 (41%)	1593 (42%)	9125 (43%)
**mPAP (mmHg)**					
Median [Q1, Q3]	30 [26, 36]	48 [41, 56]	31 [26, 37]	47 [40, 54]	38 [29, 48]
Missing	0 (0%)	0 (0%)	0 (0%)	0 (0%)	0 (0%)
**sPAP (mmHg)**					
Median [Q1, Q3]	47 [39, 57]	78 [66, 92]	46 [38, 57]	77 [65, 89]	60 [45, 78]
Missing	253 (4.5%)	529 (8.6%)	220 (4%)	384 (10%)	1386 (6.6%)
**CVP (mmHg)**					
Median [Q1, Q3]	8 [5, 11]	8 [5, 13]	7 [4, 11]	8 [5, 12]	8 [5, 12]
Missing	3533 (63%)	2593 (42%)	3935 (72%)	1599 (42%)	11,660 (55%)
**PAWP (mmHg)**					
Median [Q1, Q3]	16 [11, 21]	10 [7, 14]	17 [12, 22]	10 [8, 14]	13 [9, 19]
Missing	0 (0%)	0 (0%)	0 (0%)	0 (0%)	0 (0%)
**CO (L/min)**					
Median [Q1, Q3]	5.2 [4.3, 6.4]	3.6 [2.9, 4.5]	5.4 [4.5, 6.6]	4 [3.3, 4.8]	4.5 [3.6, 5.7]
Missing	0 (0%)	0 (0%)	0 (0%)	0 (0%)	0 (0%)
**CI (L/(min·m^2^))**					
Median [Q1, Q3]	2.8 [2.3, 3.4]	2.1 [1.7, 2.5]	2.6 [2.2, 3.1]	2 [1.7, 2.4]	2.4 [1.9, 2.9]
Missing	78 (1.4%)	191 (3.1%)	46 (0.84%)	85 (2.2%)	400 (1.9%)
**PVR (WU)**					
Median [Q1, Q3]	2.9 [2, 3.8]	9.6 [6.9, 14]	2.5 [1.7, 3.5]	8.3 [6.3, 11]	4.7 [2.6, 8.7]
Missing	0 (0%)	0 (0%)	0 (0%)	0 (0%)	0 (0%)

PVR: Pulmonary Vascular Resistance; WU: Wood Units; WHO FC: World Health Organisation Functional Class; BMI: Body Mass Index; BSA: Body Surface Area; 6MWD: 6-Minute Walk Distance; BNP: Brain Natriuretic Peptide; mPAP: Mean Pulmonary Arterial Pressure; sPAP: Systolic Pulmonary Arterial Pressure; CVP: Central Venous Pressure, PAWP: Pulmonary Arterial Wedge Pressure; CO: Cardiac Output; CI: Cardiac Index.

### Association of sex and survival in PH

**Fig. 1:**
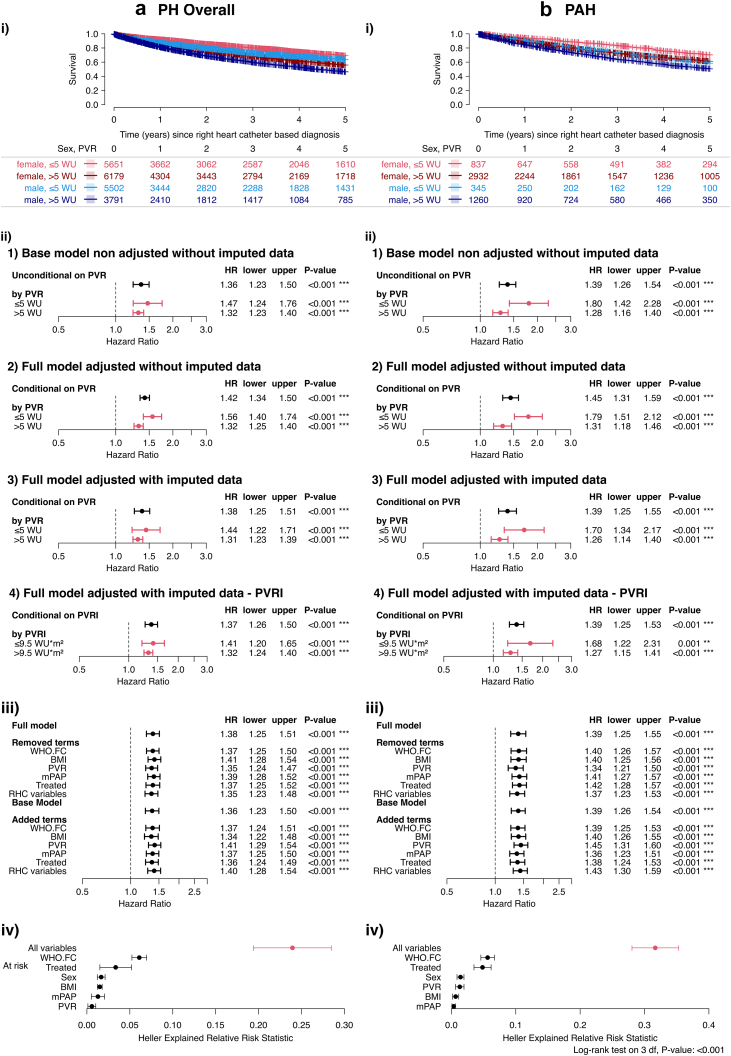
Sex Differences in Survival. a) Patients with pulmonary hypertension (21,123 patients). Subpanel i) shows the Kaplan–Meier survival analysis with 95% confidence bands by sex and pulmonary hypertension severity (log-rank test: p < 0.001). Subpanel ii) displays hazard ratios comparing men to women. 1) Results from the base-model using unimputed data, including the interaction term of the dichotomised PVR. The model is only adjusted for centre and diagnosis decade as strata and age, as natural spline with 2 degrees of freedom. 2) Results from the full model similar to 1). The model is additionally adjusted for WHO FC, BMI, PVR, mPAP and PH treatment. 3) Results from the full model similar to b) but using the imputed data set (Wald z-test: p < 0.001). 4) Results from the model similar to 3) but PVR is replaced by the PVR index as a covariate; this analysis was restricted to patients with available PVR index. The plots show the hazard ratios for men compared to women. Subpanel iii) shows results from a sensitivity analysis, and subpanel iv) illustrates the Heller Explained Relative Risk within the PH cohort. b) Patients with pulmonary arterial hypertension (PAH, 5374 patients). i) Kaplan–Meier survival analysis stratified by sex and pulmonary hypertension severity (log-rank test: p < 0.001). ii) Hazard ratios for men compared to women (Wald z-test: p < 0.001). iii) Sensitivity analysis, and iv) Heller Explained Relative Risk in the PH and PAH population. Kaplan–Meier estimates were compared using log-rank tests, while the statistical significance of survival effects was assessed via Type-III likelihood-ratio tests and individual coefficient significance was evaluated using Wald z-tests. PH = pulmonary hypertension; PAH = pulmonary arterial hypertension; PVR = pulmonary vascular resistance, WU = wood units; WHO FC = WHO functional class; BMI = body mass index; mPAP = mean pulmonary arterial pressure; HR = hazard ratio; lower/upper = lower and upper limits of the 95% confidence interval of the HR.

In the overall study population of 29,838 patients, female sex was associated with significantly higher survival rates compared to males (Figure E4). This was accompanied by significantly higher hazard ratios (HR) for men (Figure E4). For subsequent in-depth analysis, we focused on patients with complete haemodynamic data (21,123 patients; Figure E1). We stratified the overall study population by PH severity (using PVR >5 WU to define severe PH^23^) and sex (Table 1). Only the body-size dependent variables height, weight (but not BMI) and cardiac output (but not cardiac index) showed sex differences (Table 1). Female patients had higher baseline PVR, though exercise capacity and B-type natriuretic peptide were comparable (Table 1). Kaplan–Meier analysis indicated significantly higher overall survival for female patients with both non-severe and severe PH (Fig. 1a). The 1-, 3-, and 5-year survival rates for female patients with non-severe PH were 92%, 80%, and 69%, respectively, while male patients had significantly lower survival rates (88%, 73%, and 63%; log-rank p < 0.001; Fig. 1a). Similarly, females with severe PH survived longer than males with severe PH (86%, 69%, and 56% vs. 81%, 60%, and 46%; log-rank p < 0.001; Fig. 1a). Accordingly, unadjusted hazard ratios (HR) for males were significantly increased (HR [95% confidence interval] 1.47 [1.24, 1.76], Wald z-test p < 0.001 for non-severe PH and 1.32 [1.23, 1.40], Wald z-test p < 0.001 for severe PH; Fig. 1a). Likewise, increased HRs were noted after adjustment for confounders (full model including adjustment for WHO FC, BMI, mPAP, PVR, and PH-targeted treatment, Fig. 1a). In the PVR index-adjusted model, males again showed an increased hazard ratio (HR 1.37, 95% CI 1.26–1.50; Wald z-test p < 0.001; Fig. 1a), confirming the robustness of the presented findings (Fig. 1a).

In-depth sensitivity analyses were performed: Adding or removing confounders from the base or full models in the Cox regressions did not change the results, confirming their reliability (Fig. 1a). Heller's explained relative risk analysis indicated a significant impact of sex on mortality risk (Fig. 1a).

### Subgroup analyses based on PH-aetiology and use of PH-targeted drugs

We performed subgroup analyses in patients with PAH, pooled non-PAH PH patients, and separately for PH Groups 2, 3, 4, and mixed or unclassified PH.

Baseline characteristics of patients with PAH (n = 5374) stratified by PH severity and sex again indicated a tendency of female patients towards higher PVR compared to men at baseline (Table E1). Nevertheless, Kaplan–Meier analysis showed better survival rates in females as compared to males, in both moderate and severe PAH (Fig. 1b). These results were confirmed in the various Cox regression models, as shown in Fig. 1b. Sensitivity analyses and Heller's explained relative risk analysis indicated a robust and strong association between sex and survival in PAH (Fig. 1b).

**Fig. 2:**
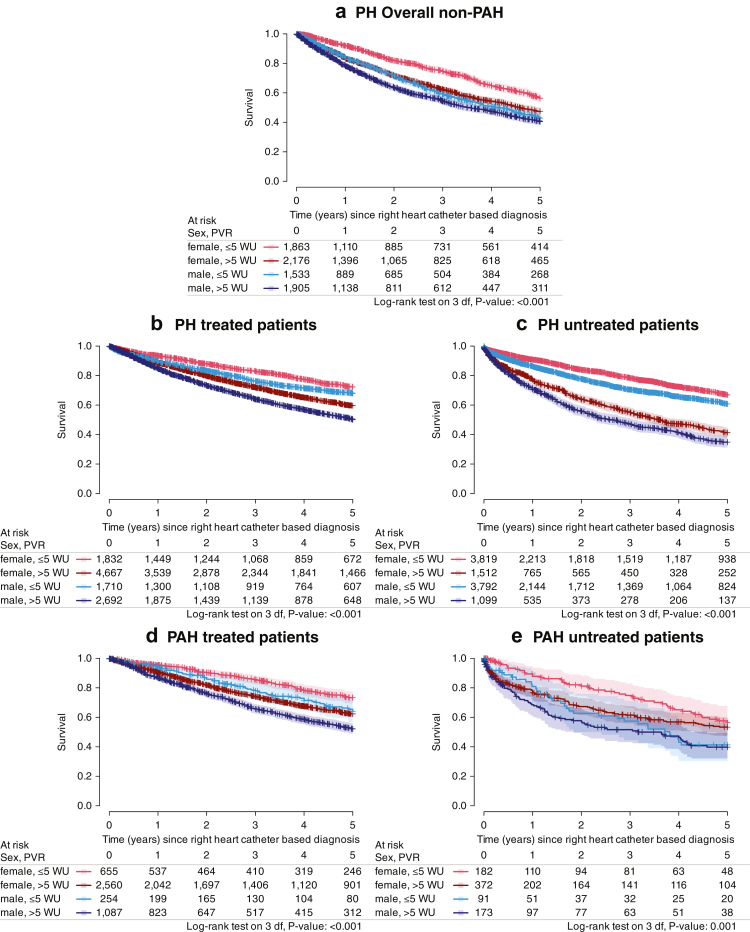
Kaplan–Meier Analysis. a) Kaplan–Meier Analysis for the Overall non-PH PH study population stratified by sex and PVR background (log-rank test: p < 0.001). b) Kaplan–Meier Analysis for treated patients with PH stratified by sex and PVR background (log-rank test: p < 0.001). c) Kaplan–Meier Analysis for untreated patients with PH stratified by sex and PVR background (log-rank test: p < 0.001). d) Kaplan–Meier Analysis for treated patients with PAH stratified by sex and PVR background (log-rank test: p < 0.001). e) Kaplan–Meier Analysis for untreated patients with PAH stratified by sex and PVR background (log-rank test: p < 0.001). Kaplan–Meier estimates were compared using log-rank tests. PH = pulmonary hypertension; PVR = pulmonary vascular resistance, PAH = pulmonary arterial hypertension; WU = wood units; WHO FC = WHO functional class; BMI = body mass index; mPAP = mean pulmonary arterial pressure; HR = hazard ratio; lower/upper = lower and upper limits of the 95% confidence interval of the HR.

Similarly, in non-PAH PH patients (n = 7477, Table E2), men had poorer survival than women regardless of PH severity (Fig. 2a). In this group, HR for men were 1.65 [1.45, 1.87] (Wald z-test p < 0.001) in non-severe PH and 1.33 [1.22, 1.45] (Wald z-test p < 0.001) in severe PH (Figure E5). Detailed analyses in PH Groups 2–4 and in the mixed/unclassified group consistently demonstrated an association between sex and survival (Table E3 and Figure E6). In Cox regression models, males showed higher hazard ratios across groups: 1.65 (95% CI 1.38–1.97) in PH Group 2, 1.41 (95% CI 1.23–1.61) in PH Group 3, 1.40 (95% CI 1.28–1.54) in PH Group 4, and 1.24 (95% CI 1.10–1.38) in the mixed/undefined group (Figure E6).

10,222 patients (48%) of the overall PH group and 818 patients (15%) of the PAH group did not receive any PH-targeted treatment during all follow-up visits available. Baseline characteristics of patients with treated and non-treated PH and PAH are shown in Tables E4 and E5. For treated patients, the distribution of PH-targeting drugs is shown in Table E6, indicating that PDE5 (phosphodiesterase type 5) inhibitors were the mostly used drugs. Significant female advantage of survival was again documented for all four groups (treated and non-treated PH; treated and non-treated PAH), as shown by Kaplan–Meier analyses (Fig. 2b–e) as well as Cox regression analyses (Figure E5).

### Association of sex and survival in PH Depending on age of diagnosis

**Fig. 3:**
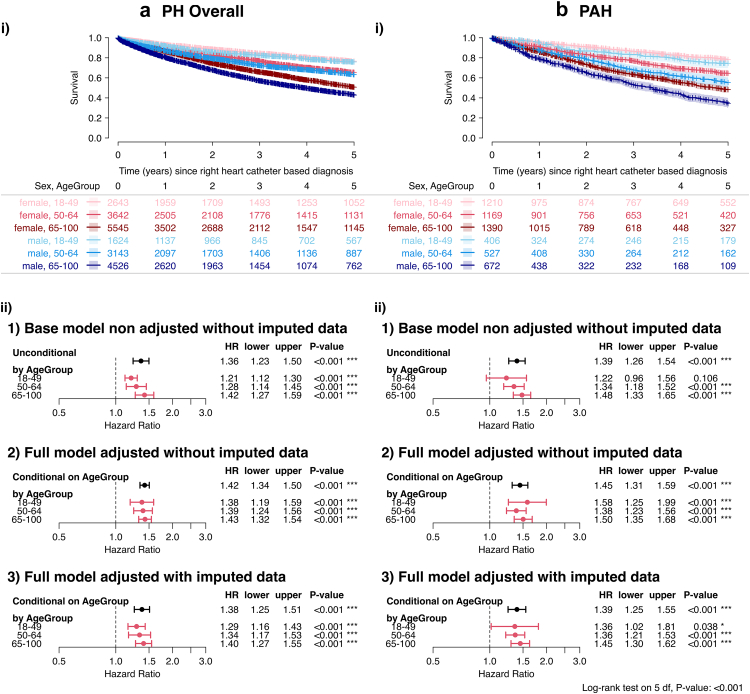
Survival and Hazard Ratios by Age and Sex. a) i) Kaplan–Meier survival curves (log-rank test: p < 0.001) and ii) Hazard Ratios (comparing men to women; Wald z-test: p < 0.001) ii) stratified by age and sex for the overall PH population. b) i) Kaplan–Meier survival curves (log-rank test: p < 0.001) and ii) Hazard Ratios (comparing men to women; Wald z-test: p < 0.001) stratified by age and sex for the PAH population. The Cox PH models were calculated for: **1)** the base-model using unimputed data, including the interaction term of the dichotomised Age Group and Sex. The model is only adjusted for centre and diagnosis decade as strata. **2)** Results from the full model similar to 1). The model is additionally adjusted for WHO FC, BMI, PVR, mPAP and PH treatment. **3)** Results from the full model similar to 2) but using the imputed data set. The plots show the hazard ratios for men compared to women. Kaplan–Meier estimates were compared using log-rank tests, while the statistical significance of survival effects was assessed via Type-III likelihood-ratio tests and individual coefficient significance was evaluated using Wald z-tests. PH = pulmonary hypertension; PAH = pulmonary arterial hypertension; WHO FC = WHO functional class; BMI = body mass index; PVR = pulmonary vascular resistance; mPAP = mean pulmonary arterial pressure; WU = wood units; HR = hazard ratio; lower/upper = lower and upper limits of the 95% confidence interval of the HR.

Table E7 shows the baseline characteristics stratified by age and sex. Kaplan–Meier analysis again showed better female survival rates for all age groups in PH as well as patients with PAH (Fig. 3a and b). Correspondingly, significantly increased HRs of male patients were calculated for all age groups in the overall PH study population (Fig. 3a). Similar association of age, sex, and survival was observed in the PAH subgroup with less rigorous association for patients between 18 and 49 years (Fig. 3b).

### Association of sex and survival in PH Depending on estimated risk of mortality

**Fig. 4:**
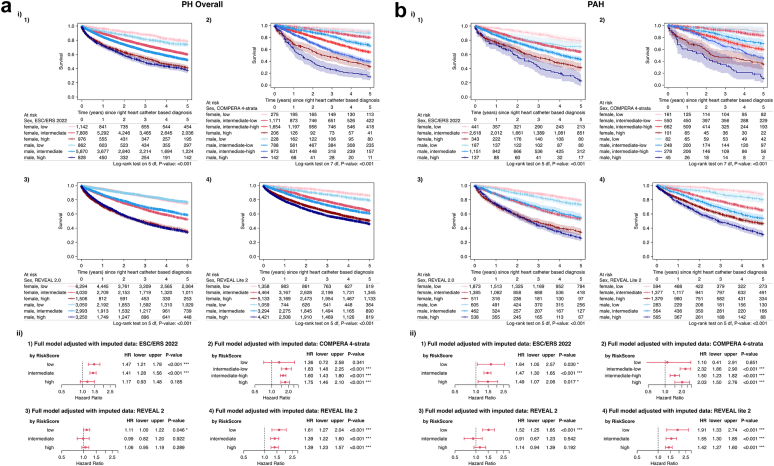
Impact of Sex on Estimated Risk of Mortality for patients with a) PH and with b) PAH. (i) Kaplan–Meier survival curves, illustrating the impact of sex on risk scores. Subpanel 1) shows the ESC/ERS 2022 risk score (log-rank test: p < 0.001 for patients with a) PH and b) PAH), subpanel 2) the COMPERA 4-strata risk score (log-rank test: p < 0.001 for patients with a) PH and b) PAH), subpanel 3) the Reveal 2.0 risk score (log-rank test: p < 0.001 for patients with a) PH and b) PAH), and subpanel 4) the Reveal Lite 2 risk score (log-rank test: p < 0.001 for patients with a) PH and b) PAH). (ii) Hazard ratios for according to sex (comparing men to women) within risk classes using different scoring systems (ESC/ERS 2022: Wald z-test low p < 0.001, intermediate p < 0.001 and high p = 0.185; COMPERA 4-strata: Wald z-test low p = 0.341, intermediate-low p < 0.001, intermediate-high p < 0.001 and high p < 0.001; REVEAL 2 Wald z-test low p = 0.046, intermediate p = 0.922 and high p = 0.289; REVEAL Lite 2 Wald z-test low p < 0.001, intermediate p < 0.001 and high p < 0.001 for patients with a) PH and ESC/ERS 2022: Wald z-test low p = 0.030, intermediate p < 0.001 and high p = 0.017; COMPERA 4-strata: Wald z-test low p = 0.851, intermediate-low p < 0.001, intermediate-high p < 0.001 and high p < 0.001; REVEAL 2 Wald z-test low p < 0.001, intermediate p = 0.542 and high p = 0.192; REVEAL Lite 2 Wald z-test low p < 0.001, intermediate p < 0.001 and high p < 0.001 for patients with b) PAH). The Cox PH models were calculated using the imputed data set and are adjusted for WHO FC, BMI, PVR, mPAP and PH treatment, as well as centre and diagnosis decade as strata and age, as natural spline with 2 degrees of freedom. Kaplan–Meier estimates were compared using log-rank tests, while the statistical significance of survival effects was assessed via Type-III likelihood-ratio tests and individual coefficient significance was evaluated using Wald z-tests. WHO FC = WHO functional class; BMI = body mass index; PVR = pulmonary vascular resistance; mPAP = mean pulmonary arterial pressure; PH = pulmonary hypertension; PAH = pulmonary arterial hypertension; PVR = pulmonary vascular resistance, WU = wood units; HR = hazard ratio; lower/upper = lower and upper limits of the 95% confidence interval of the HR.

We stratified patients according to the ESC/ERS 2022, COMPERA 4-strata, REVEAL 2.0, and REVEAL Lite 2 risk scores to assess the impact of sex on estimated risk of mortality. Using the ESC/ERS 2022 risk stratification scheme, 2004 (11.5%), 13,678 (78.2%), and 1804 (10.3%) patients were classified as low-, intermediate-, and high-risk, respectively, in the overall study population. Female patients had higher survival rates compared to men in all respective risk groups (Fig. 4a). Differences were most pronounced in the intermediate-risk group and less so in the low- and high-risk groups. Similarly, 2417 (12.3%), 7758 (39.3%), and 9554 (48.4%) patients were classified as low-, intermediate-, and high-risk using the REVEAL lite 2 score. For the COMPERA 4-strata risk score, 503 (9.3%), 1959 (36%), 2627 (48.3%), and 348 (6.4%) patients were classified as low-, intermediate-low, intermediate-high, and high-risk, respectively. Females had higher survival rates in all risk groups for both scores (Fig. 4a). Cox regression analyses supported these findings, showing higher hazard ratios for men across all three risk scores (Fig. 4a). In contrast, the REVEAL 2.0 risk score, which includes sex (and age) in the risk calculation scheme, showed no meaningful survival differences between male and female patients (Fig. 4a). In the PAH group (Group 1 PH), similar results were observed; the REVEAL 2.0 risk score was again not influenced by sex disparities (except for the low-risk group), in contrast to the ESC/ERS 2022, COMPERA 4-strata, and REVEAL lite 2 risk scores (Fig. 4b).

### Association of sex and survival in PH Depending on comorbidities

**Fig. 5:**
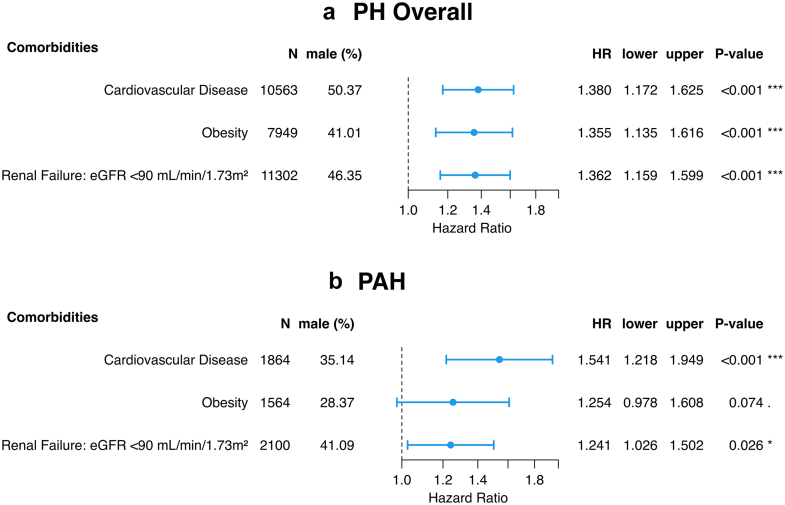
Impact of Cardiovascular Comorbidities, Obesity and Chronic Kidney Disease on Sex-Associated Survival Differences. Hazard ratios comparing men to women for the a) PH population (10,563 patients with PH with reported cardiovascular comorbidities Wald z-test: p < 0.001; 8352 patients with PH with reported obesity Wald z-test: p < 0.001; 11,302 patients with PH with recorded chronic kidney disease Wald z-test: p < 0.001) and the b) PAH population (1864 patients with PAH with reported cardiovascular comorbidities Wald z-test: p < 0.001; 1686 patients with PAH with reported obesity Wald z-test: p = 0.062; 2100 patients with PAH with recorded chronic kidney disease Wald z-test: p = 0.026). The Cox PH models were calculated using the imputed data set and are adjusted for WHO FC, BMI, PVR, mPAP and PH treatment, as well as centre and diagnosis decade as strata and age, as natural spline with 2 degrees of freedom. The statistical significance of survival effects was assessed via Type-III likelihood-ratio tests and individual coefficient significance was evaluated using Wald z-tests. PH = pulmonary hypertension; WHO FC = WHO functional class; BMI = body mass index; PVR = pulmonary vascular resistance; mPAP = mean pulmonary arterial pressure; PAH = pulmonary arterial hypertension; HR = hazard ratio; lower/upper = lower and upper limits of the 95% confidence interval of the HR.

In the overall study cohort, 10,563 patients (61%) had cardiovascular comorbidities, 8352 (38%) were obese, and 11,302 (76%) had chronic kidney disease; data originating from centres reporting cardiovascular disease and obesity, and from patients with reported eGFR, respectively (Fig. 5). The distribution of comorbidities within the study population is presented in Table E8. Baseline characteristics stratified by sex and presence/absence of cardiovascular comorbidities, obesity, as well as chronic kidney disease are shown in Tables E9–E11, again with females showing a tendency towards more pronounced pulmonary vascular impairment at baseline. Predicted HRs revealed significantly higher survival rates of females in the presence of cardiovascular comorbidities, obesity or chronic kidney disease in both the overall PH study population and the PAH group (Fig. 5a and b).

### Association of sex and survival in PH Depending on race

14,405 of the included patients (68% of the overall study population) were classified by their respective PH expert centres into race groups. Of these, 11,879 (82.5%) were White, 1740 (12.2%) were Black, 610 (4.2%) were Asian, and 176 (1.2%) were other/mixed. The ratio between incident females and males was 1.1:1 in White, while it was 1.6:1 in Black, 1.6:1 in Asian, and 2.1:1 in the other/mixed group. In Europe and in America, 96% and 80.2% of the patients, respectively, were classified as White. In Asia and the rest of the world, 79% were classified as Asian. Kaplan–Meier analysis showed no major survival disparities between Black, Asian, and White patients with PH, independent of sex (Figure E7). Correspondingly, the HRs for the Asian PH group showed no significant difference to the White PH group in all models (Figure E7). However, slightly increased HRs were obtained for Black patients with PH throughout the full model with imputed data (Figure E7).

When stratifying for both sex and race (Table E12), a major difference was noted. For the White population, the male survival disadvantage was again noted, both in the moderate (PVR ≤5 WU) and the severe PH subgroups (PVR >5 WU), as evident from Kaplan–Meier statistics and Cox-regression (Figure E7). In contrast, in both the Black and the Asian patients with PH, survival did not markedly differ between females and males in either Kaplan–Meier or Cox regression analyses, regardless of PH severity (Figure E7). However, interaction analysis examining race × sex did not demonstrate statistical significance. Numbers were too low for separate analysis of the different PH groups with respect to the impact of race on survival.

## Discussion

This study examined the effect of sex on survival in patients with PH, including PAH and non-PAH PH (Group 2–5) patients. Our key findings include: (i) Male sex was consistently associated with significantly lower survival rates compared to females, regardless of age, PH severity, PH aetiology, obesity, or cardiovascular comorbidities; (ii) Sex significantly influenced survival predictions in commonly used risk scores, except for REVEAL 2.0, which explicitly considers male sex as a non-modifiable risk factor; (iii) The survival disadvantage of males was independent of treatment with PAH-specific drugs, indicating that better treatment responses in women cannot account for this disparity; and (iv) The sex-related survival differences were fully reproduced in White patients with PH, but not in Black or Asian populations.

Sex differences in various cardiovascular diseases are well-established.^9^^,^^28^, ^29^, ^30^, ^31^ In contrast, understanding sex differences in PH is still developing, despite growing recognition of its importance.^18^ Our study, to the best of our knowledge the largest to date investigating sex differences in PH, found that male sex was independently and significantly associated with poorer survival rates among over 21,000 patients with PH. This association persisted after adjusting for confounding variables such as pulmonary haemodynamics, exercise capacity, and treatment, and was consistent across analyses stratified by age groups, PH severity, presence/absence of obesity and further cardiovascular comorbidities, and when specifically analysing for either the PAH group, the combined non-PAH PH group, PH Groups 2–4 or mixed/undefined, respectively. Moreover, the robustness of our findings was confirmed through in-depth sensitivity analyses including Heller Explained Relative Risk.

Additionally, sex impacted survival within estimated risk groups using established risk scores, including the ESC/ERS 2022 risk scheme, COMPERA 4-strata risk score, and the REVEAL lite 2 score. Notably, the REVEAL 2.0 risk score, which includes male sex as non-modifiable risk factor, did not show sex-specific differences within risk classes.^32^ Thus, we hypothesise that integrating sex into risk stratification tools may narrow the sex gap in PH survival prognostication.

Compared to other contemporary registries, the distribution of PH groups in PVRI GoDeep differs substantially.^7^^,^^33^^,^^34^ For example, PHAR, which includes only patients with PAH and CTEPH from expert centres in the United States, and ASPIRE, a single-centre referral registry from Sheffield, illustrate different case mixes.^7^^,^^33^^,^^34^ In contrast, PVRI GoDeep integrates data from more than 31,000 hemodynamically characterised patients, collected exclusively at PH expert centres worldwide.^20^ Such differences in scope, inclusion criteria, and referral patterns likely account for the observed differences in baseline characteristics between GoDeep and other contemporary registries.

Cluster analyses of patients with idiopathic PAH identified a subset characterised by younger age and female sex, which exhibited better treatment response and survival compared to groups mainly defined by male sex, age, comorbidities, and/or impaired lung diffusion capacity.^35^ Similarly, REVEAL registry analyses indicated that besides a higher prevalence of PAH among women, men may have more severe haemodynamic limitations at the time of diagnosis.^36^ Our study, however, revealed more severe haemodynamic impairment in females upon RHC-based diagnosis, yet survival probability for females was comparable across both studies.^36^ Notably, differences emerged when stratifying by age: in the REVEAL registry, sex differences were observed only in patients with PAH diagnosed at age ≤60 years, while adjusted Cox regression analysis did not reach statistical significance for the overall study population of 2059 patients with PAH.^36^ In contrast, our analysis noted poorer survival rates of males across all three age groups (18–49, 50–64, >64 years) for both the overall PH group and PAH group. More granular documentation of the sex disparity in PH survival in the current analysis may have been enabled by the significantly higher sample size.

It has been suggested that better treatment response of women with PH or PAH may contribute to their higher survival probabilities.^6^^,^^35^^,^^37^ Our large-scale database identified a substantial cohort of patients with PH and PAH who had never received any PAH-targeting medication. Notably, in these entirely treatment-naïve patients, the survival disadvantage for males persisted. Therefore, the male survival disadvantage cannot be attributed to better therapy responses of women.

This study furthermore investigates the race-dependency of the male survival disadvantage in PH among a large cohort of patients with P(A)H. When not segregating for sex, survival curves and hazard ratios for White and Asian populations did not significantly differ, but slightly increased hazard ratios were noted in Black PH as compared to White patients with PH. This corresponds to conflicting reports in this field so far,^38^^,^^39^ in contrast with other cardiovascular diseases, for which higher mortality rates for Black men and women as compared to White patients have been clearly demonstrated.^40^ Interestingly, the female-male survival disparity was replicated only in the White cohort, with no such differences in patients of Black or Asian backgrounds. As a word of caution, it must be kept in mind that the population classified as White was by far the largest. Further studies including larger proportions of non-white populations are thus mandatory to verify whether the male survival disadvantage in PH is indeed only a “white men phenomenon”.

Although not designed to elucidate the underlying mechanisms of the sex-disparity in P(A)H survival, our data challenge the “vascular hypothesis” (more severe/progressive pulmonary vascular remodelling/resistance in males). First, PVR was higher in female patients. Second, the molecular mechanisms of lung vascular remodelling differ substantially among PH groups,^41^^,^^42^ yet the male survival disadvantage persisted across both PAH and non-PAH cohorts. Third, all current PH-specific therapies target PVR, but the female-male survival disparity is independent of these treatments. This may favour the “right ventricular” hypothesis (worse right ventricular–pulmonary artery (RV-PA) coupling in male patients with PH) of the sex disparity of PH survival. The RV function is a common denominator of survival in all hitherto investigated PH entities.^43^ Sophisticated analyses of RV-PA coupling in patients with PAH indeed documented worse RV function in men as compared to women, independent of PH severity as judged by PVR.^9^ This corresponds to testosterone-dependent RV malfunction noted in a rodent model with fixed increase of RV afterload due to pulmonary artery banding.^36^ A small study of Dutch patients with PAH indicated that while PVR decreased similarly after initiating PAH-targeted treatment, RV ejection fraction improved in females but deteriorated in males.^44^ The PVRI GoDeep meta-registry currently lacks detailed analyses of RV function (e.g., echo, MRI), which should be envisioned in future studies to further elucidate the putative disadvantageous male RV function as underlying mechanism of the sex-disparity in PH survival.

Oestrogen and its metabolites impact lung vascular remodelling and have been linked to better survival of females with PH, despite a higher incidence of the disease in females (“oestrogen paradox”).^14^^,^^36^^,^^37^ If the sex disparity is primarily attributed to differences in RV function, it may be associated with oestrogen-dependent cardiac signalling pathways. However, our analysis of different age groups demonstrated the female survival advantage in both pre-menopausal and post-menopausal women. If confirmed, the finding that sex disparities in PH survival are most pronounced in white patients suggests the need to consider race-associated X- and Y-linked genetic and epigenetic factors, as well as broader social, environmental, and healthcare-related determinants that may contribute to these differences.

This study is limited by its retrospective design, which excludes definite pinpointing of causal relationships between the observed differences. Moreover, as to be expected for real world data collection, we have substantial missingness in some of the categories, though we only included patients with documented baseline haemodynamics. White patients represented the largest group in spite of the global design of the GoDeep meta-registry, which does not correspond to the worldwide distribution of ethnicities. Nevertheless, the large sample size and sophisticated statistical approaches, considering various putative confounders, performing in-depth sensitivity analyses and showing reproducibility in various subgroups of patients with PH, substantiate the robustness of our findings.

Taken together, our study indicates worse survival probability for male patients with PH, including both PAH and non-PAH entities, regardless of PH severity, PH aetiology, age, obesity, cardiovascular comorbidities as well as presence or absence of PAH-specific treatment. Including male sex as non-modifiable risk factor may improve the accuracy of risk assessment scores. The surprising finding that the sex disparity in PH survival may be race-dependent opens a novel angle of research but needs further verification.

## Contributors

Study conceptualisation: A.Y., K.T., W.S.; Study design and data collection: A.Y., J.S.A., M.F., J.W., D.G.K., L.H., A.L., M.R.W., A.B., P.M.H., Z.K., C.A.E., E.G., A.J.S., R.T.Z., G.K., H.O., M.L., S.U., T.T., I.A., S.Y.C., J.E., A.J., J.C., J.P.Z., R.F., Y.S., O.T., S.S., Z.Z., Z.Zh., A.A., G.G., M.Fr., P.G.W., K.K., H.M., S.G., L.S., H.S., K.S., A.A., E.D., R.W.M., H.A.G., F.G., K.T., H.R.C., E.B., W.S.; Data analysis: A.Y., M.F., J.W.; Drafting of the manuscript: A.Y., M.F., K.T., E.L.B., W.S.; Accessed and verified data: A.Y., M.F., J.W., W.S.; Critical revision and approval of the manuscript for submission: A.Y., J.S.A., M.F., J.W., D.G.K., L.H., A.L., M.R.W., A.B., P.M.H., Z.K., C.A.E., E.G., A.J.S., R.T.Z., G.K., H.O., M.L., S.U., T.T., I.A., S.Y.C., J.E., A.J., J.C., J.P.Z., R.F., Y.S., O.T., S.S., Z.Z., Z.Zh., A.A., G.G., M.Fr., P.G.W., K.K., H.M., S.G., L.S., H.S., K.S., A.A., E.D., R.W.M., H.A.G., F.G., K.T., H.R.C., E.B., W.S. Consortium: Data contribution.

All authors read and approved the final version of the manuscript prior to submission.

## Data sharing statement

The data that support the findings of this study will be made available to qualified researchers upon reasonable request to the corresponding author. Available data include de-identified individual participant data and key variables underlying the analyses presented in this manuscript. Access will be granted to researchers whose proposed use of the data is consistent with the aims of the original study and complies with institutional and ethical guidelines. Data will be shared via secure file transfer.

## Declaration of interests

**AYogeswaran** reports non-financial support from the University of Giessen during the conduct of the study, research grants from the German Research Foundation, support for attending a meeting from OrphaCare and AOP, and personal fees from MSD and Ferrer outside the submitted work. **JSAnnis**, **MFünderich**, **JWilhelm** have nothing to disclose. **DGKiely** reports grants from Janssen Pharmaceuticals, National Institute of Health Research Sheffield Biomedical Research Centre, Ferrer, consulting fees from Janssen Pharmaceuticals, Ferrer, Altavant, MSD, United Therapeutics, and support for attending meetings from Janssen, Ferrer, MSD, and United Therapeutics, participation on advisory board of Janssen and MSD. **LHoward** reports personal fees and non-financial support from Janssen, personal fees from MSD, Gossamer, Altavant. **ALawrie** has nothing to disclose. **MRWilkins** reports personal fees from MorphogenIX, Janssen, Chiesi, Aerami, grants from British Heart Foundation, NIHR, personal fees from MSD, Benevolent AI, from Tiakis Biotech, outside the submitted work. In addition, Dr. Wilkins has a patent Zip12 as a drug target issued. **ABalasubramanian** has nothing to disclose. **PMHassoun** reports personal fees from Merck Co; outside the submitted work. **ZKonswa** has nothing to disclose. **CAEichstaedt** received honoraria for lectures and presentations from OMT, MSD and Ferrer, consulting fees from MSD. **EGrünig** received fees for lectures and/or consultations from Actelion, Bayer, GSK, Janssen, MSD, Pfizer and United Therapeutics; reports grants from Acceleron, Actelion, Aerovate, Bayer, Ferrer, Gossamer, Insmed, Janssen, Keros, Liquidia, Merck, MSD, Novartis, OMT, United Therapeutics, consulting fees from Actelion, Ferrer, Janssen, Merck, MSD, honoraria from Actelion, AOP, Bayer, Ferrer, GEBRO, GSK, GWT, Janssen, MSD, OMT, phev, and participation on advisory boards from Actelion, Ferrer, and MSD. **AJSweatt** eceived K23 grant support (K23HL151892) from NIH/NHLBI.**RTZamanian** has a patent FK506 for treatment of PAH issued to Stanford University. **GKovacs** reports grants from Janssen and Boehringer-Ingelheim, consulting fees from MSD, Boehringer-Ingelheim, AOP Orphan, Chiesi, Ferrer, Bayer, Janssen, GSK, Liquidia, Astra Zeneca, United Therapeutics, honoraria from MSD, Boehringer-Ingelheim, AOP Orphan, Chiesi, Ferrer, Bayer, Janssen, GSK, Liquidia, Astra Zeneca, support for attending meetings from MSD, Janssen, Boehringer-Ingelheim, AOP Orphan, and participating on advisory boards from MSD, Boehringer-Ingelheimm, Ferrer, and Liquidia. **HOlschewski** has received grants from Astra Zeneca, Bayer, IQVIA, Janssen, Liquidia, MSD, Medupdate, Menarini, Pfizer. He has received consulting fees from MSD and Menarini. He has received payments or honoraria for lectures, presentations, speakers bureaus, manuscript writing or educational events from MSD, and has also received travel support from MSD. He has a planned patent publication related to the diagnosis of PH using lipid ratios. He received grants from Bayer, IQVIA, Liquidia and Pfizer for participation on advisory Board. He holds equity interests in Bayer.**MLichtblau** received travel support and consultancy fees from Orpha Swiss, Johnson and Johnson, MSD and Gebro all unrelated to the present work. **SUlrich** receives research grants from the Swiss National Science Foundation, Zurich and Swiss Lung League and grants, travel support and consultancy fees from Orpha Swiss, Janssen SA, MSD SA, Gebro and Ideogen all unrelated to the present work. **TThenappan** served as consultant to United Therapeutics, J&J, Merck, Aria CV, Gossiermer Bio, and Altvant Science. He has received research funding from Merck, Aria CV, Tenax therapeutics, and Liquidia for clincial trials. **IAl Ghouleh** has nothing to disclose. **SYChan** reports personal fees from Janssen, United Therapeutics, and Merck as well as grant funding from Bayer and United Therapeutics. SYChan is a director, officer, and shareholder of Synhale Therapeutics and Amlysion Therapeutics. **JElwing** reports grants from United Therapeutics, Gossamer Bio, Bayer, Acceleron/Merck, Altavant, Aerovate, Tenax, Pharmosa, Actelion/Janssen, Lung LLC, personal fees from United Therapeutics, Altavant, Aerovate, Bayer, Gossamer Bio, Liquida, Acceleron/Merck, Janssen, Insmed, during the conduct of the study.**AJose** reports grants from United Therapeutics and National Institute of Health K23 Career Development, Payment from Law firm of Huff, Powell, Bailey in Atlanta Georgia, and participating on advisory boad of Merck and Janssen. **JCannon**, **JPepke-Zaba**, **HRCajigas** have nothing to disclose. **RFrantz** received consulting fees from Merck Inc, Insmed, Inhibikase, Gossamer Bio. He holds equity interests in Merck.**YSirenko**, **OTorbas** have nothing to disclose. **SSahay** reports personal fees from Gossamer Bio, Merck, Keros, Janssen, United Therapeutics, Liquidia. **ZZhai** received grants or contracts from The National Key Research and Development Program of China, and the Chinese Academy of Medical Sciences Innovation Fund for Medical Sciences.**ZZhang** has nothing to disclose. **AArvanitaki** received travel grants from MSD Greece and Innovis Pharma.**GGiannakoulas** reports Speaker fees from ELPEN Pharmaceuticals, Ferrer/Galenica, GossamerBio, Janssen Pharmaceutical Companies of Johnson & Johnson, MSD, Travel fees from ELPEN Pharmaceuticals, Ferrer/Galenica, GossamerBio, Janssen Pharmaceutical Companies of Johnson & Johnson, MSD, and Advisory board fees from GossamerBio, Janssen Pharmaceutical Companies of Johnson & Johnson, MSD. **MFrauendorf**, **PGWilliams**, **KKuronuma** have nothing to disclose. **HMatsubara** has received institutional support for basic research from Nippon Shinyaku. He received honoraria from Bayer, Janssen, Kaneka Medix, Mochida, MSD, Nippon Shinyaku, Nipro and AOP Orphan for delivering lectures. He received consulting fees for advisory related to clinical trials. He has served as a member of an advisory board for Bayer, Janssen, Mochida and MSD. He participated at the Executive Board for the international CTEPH Association without receiving compensation.**SGhio**, **LScelsi**, **HSabbour**, **KSaleh**, **AAnthi**, **EDima**, **RWMajeed** have nothing to disclose. **HAGhofrani** has received fees from Actelion, AstraZeneca, Bayer, GSK, Janssen-Cilag, Lilly, Novartis, OMT, Pfizer, and United Therapeutics. **FGrimminger** has nothing to disclose. **KTello** has received personal fees from Bayer, AstraZeneca, Gossamer. **EBrittain** has received investigator initiated grant funding and advisory board related personal fees from United Therapeutics. **WSeeger** has received consultancy fees from United Therapeutics, Tiakis Biotech AG, Liquidia, Pieris Pharmaceuticals, Abivax, Pfitzer, Medspray BV.

## Appendix A Supplementary data

Figure E1

Figure E2

Figure E3

Figure E4

Figure E5

Figure E6

Figure E7

Supplementary Figure captions and Tables

Consortium Supplement
