# Supplementary material for: Male survival disadvantage in pulmonary hypertension: independent of aetiology, age, disease severity, comorbidities and treatment
Source: eBioMedicine. 2025 Dec 16;123:106063. doi: 10.1016/j.ebiom.2025.106063 (PMC12768861; doi:10.1016/j.ebiom.2025.106063)
Supplement: Figure E1 [file mmc1.pdf]

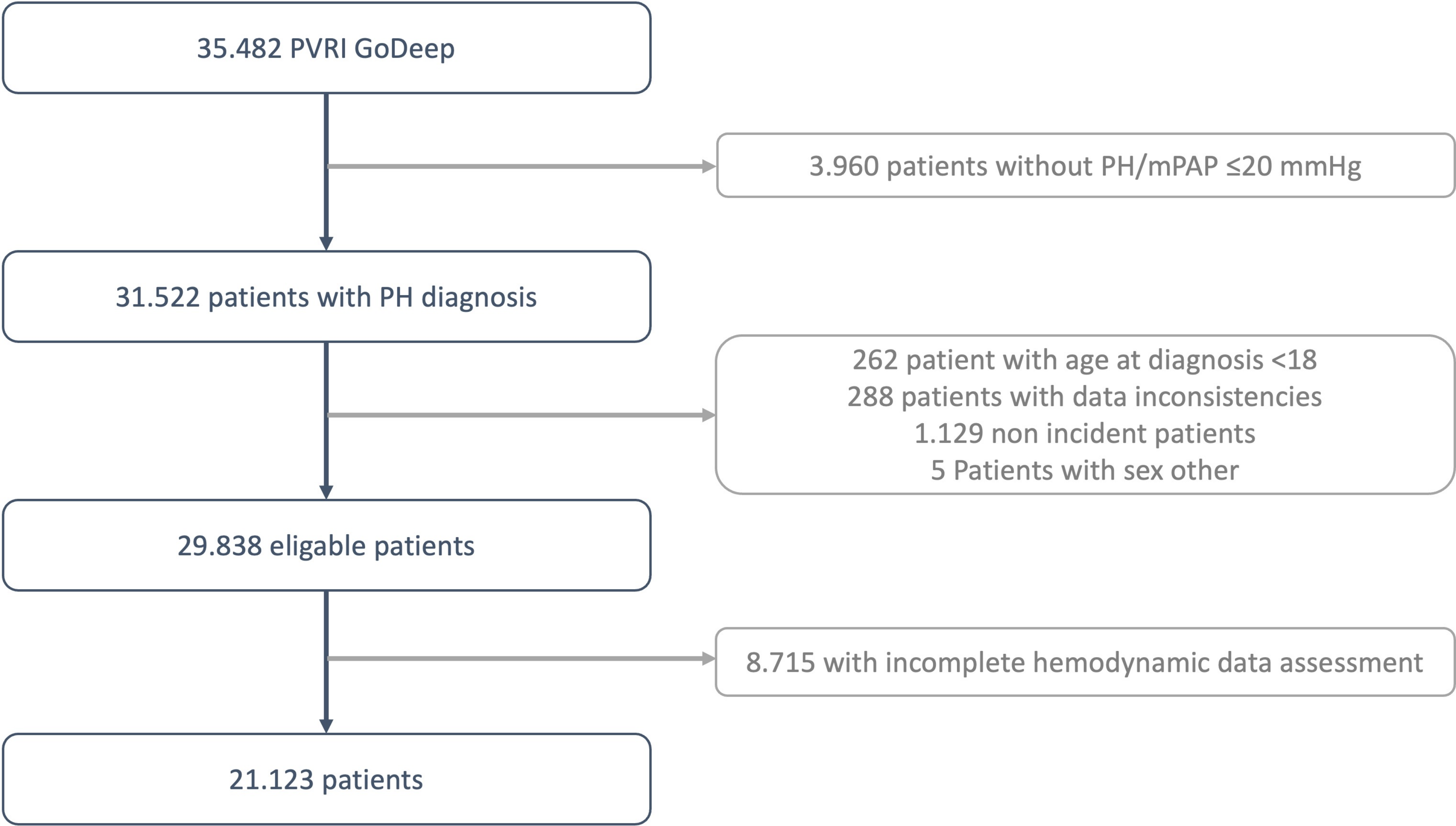
35.482 PVRI GoDeep
3.960 patients without PH/mPAP ≤20 mmHg
31.522 patients with PH diagnosis
262 patient with age at diagnosis <18
288 patients with data inconsistencies
1.129 non incident patients
5 Patients with sex other
29.838 eligable patients
8.715 with incomplete hemodynamic data assessment
21.123 patients
