# Supplementary figures and images for: Male survival disadvantage in pulmonary hypertension: independent of aetiology, age, disease severity, comorbidities and treatment
Source: eBioMedicine. 2025 Dec 16;123:106063. doi: 10.1016/j.ebiom.2025.106063 (PMC12768861; doi:10.1016/j.ebiom.2025.106063)

A)

imputed 6% missing values

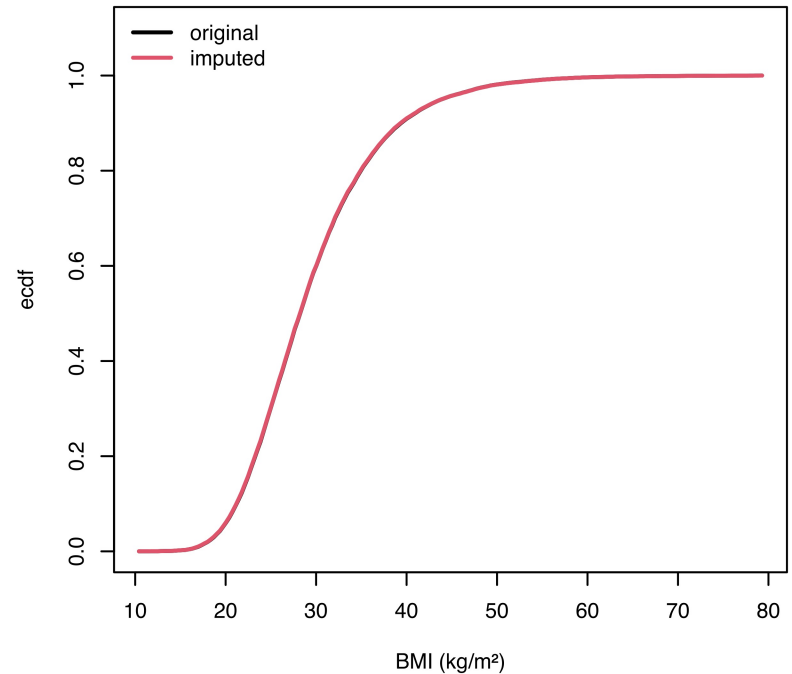

imputed 31% missing values

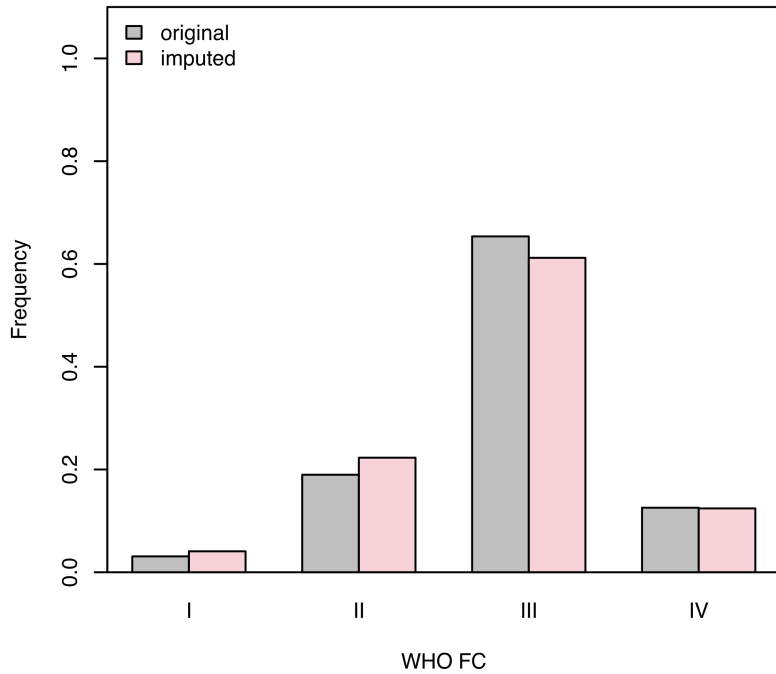

B)

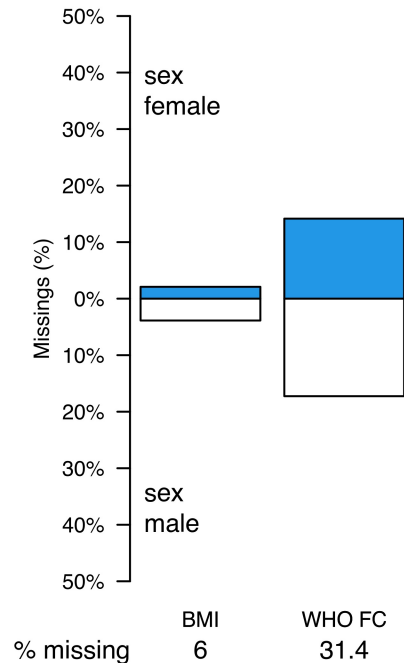

Supplement: Figure E2 [file mmc2.pdf]

**A) PH Overall**

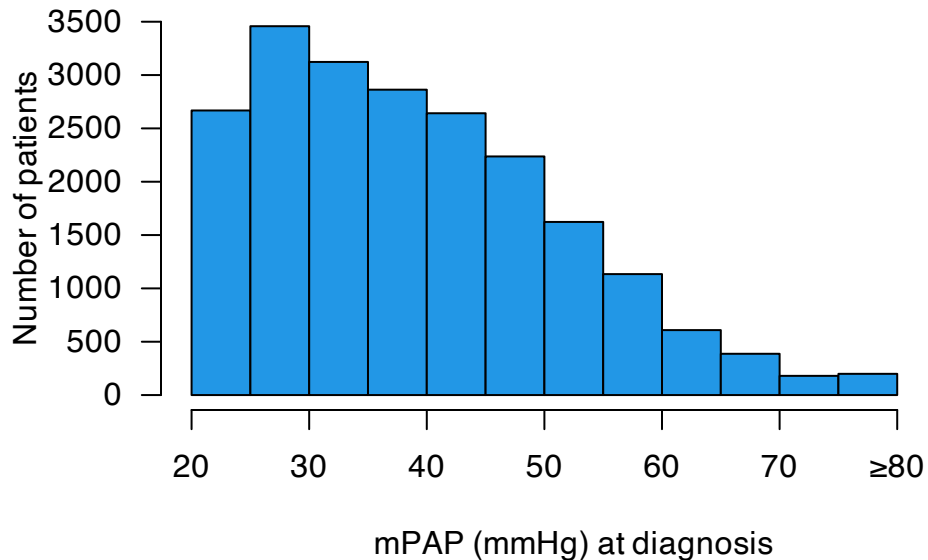

**B) PAH**

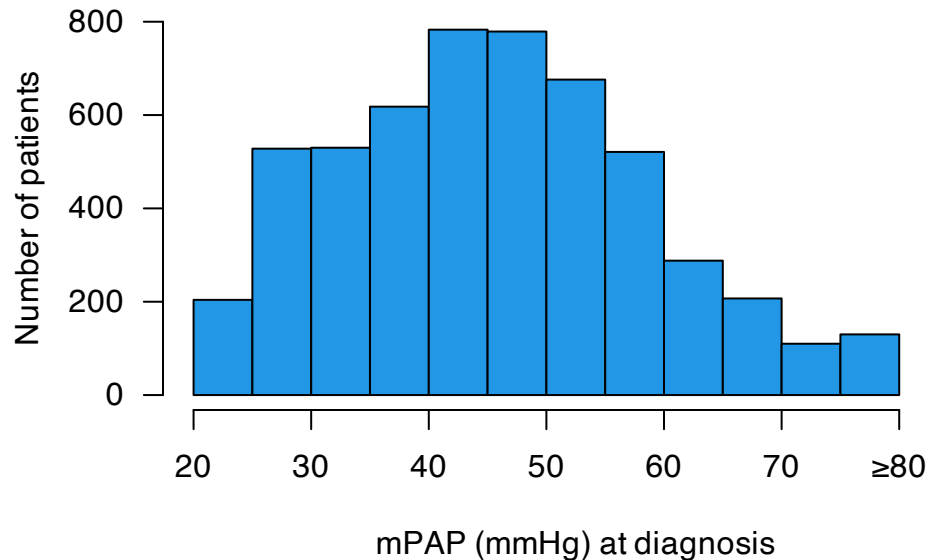

Supplement: Figure E3 [file mmc3.pdf]

# i) A) PH Overall

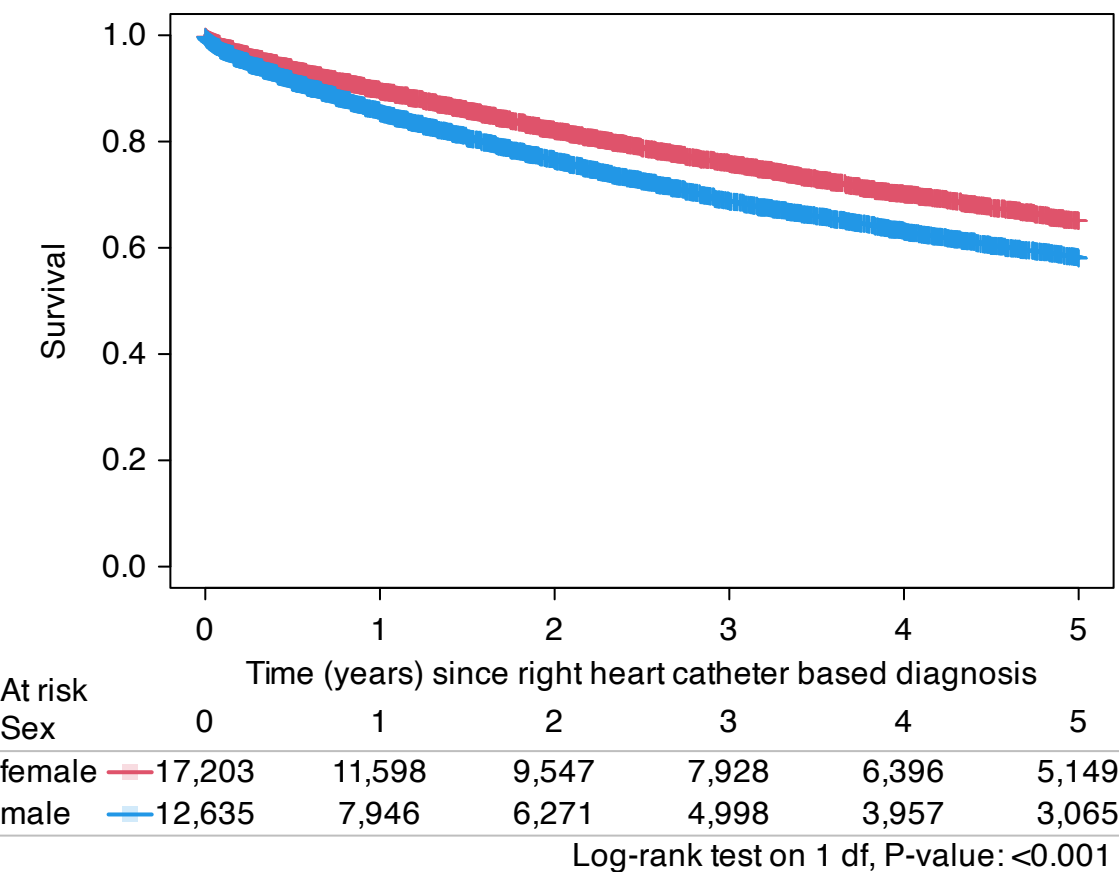

ii)

Base model non adjusted without imputed data

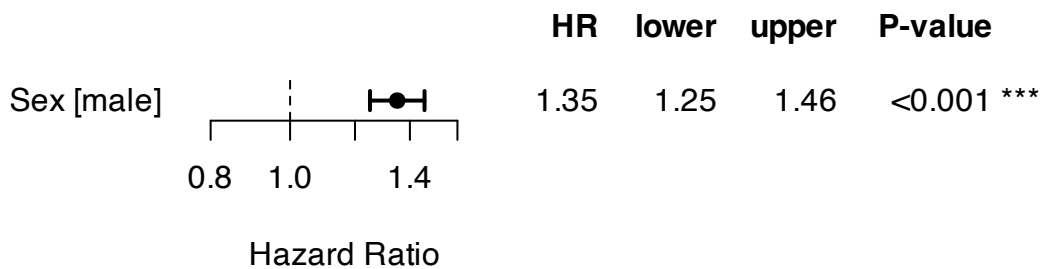

Supplement: Figure E4 [file mmc4.pdf]

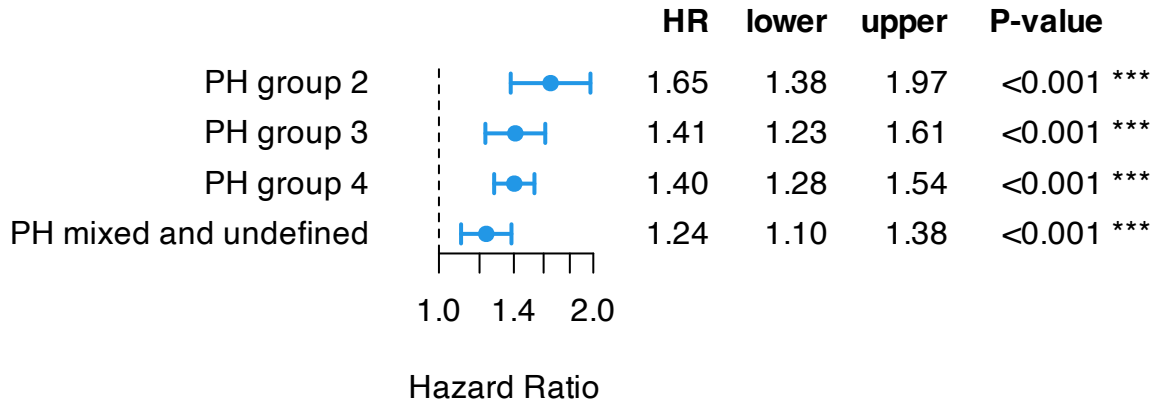

Supplement: Figure E6 [file mmc6.pdf]

A)

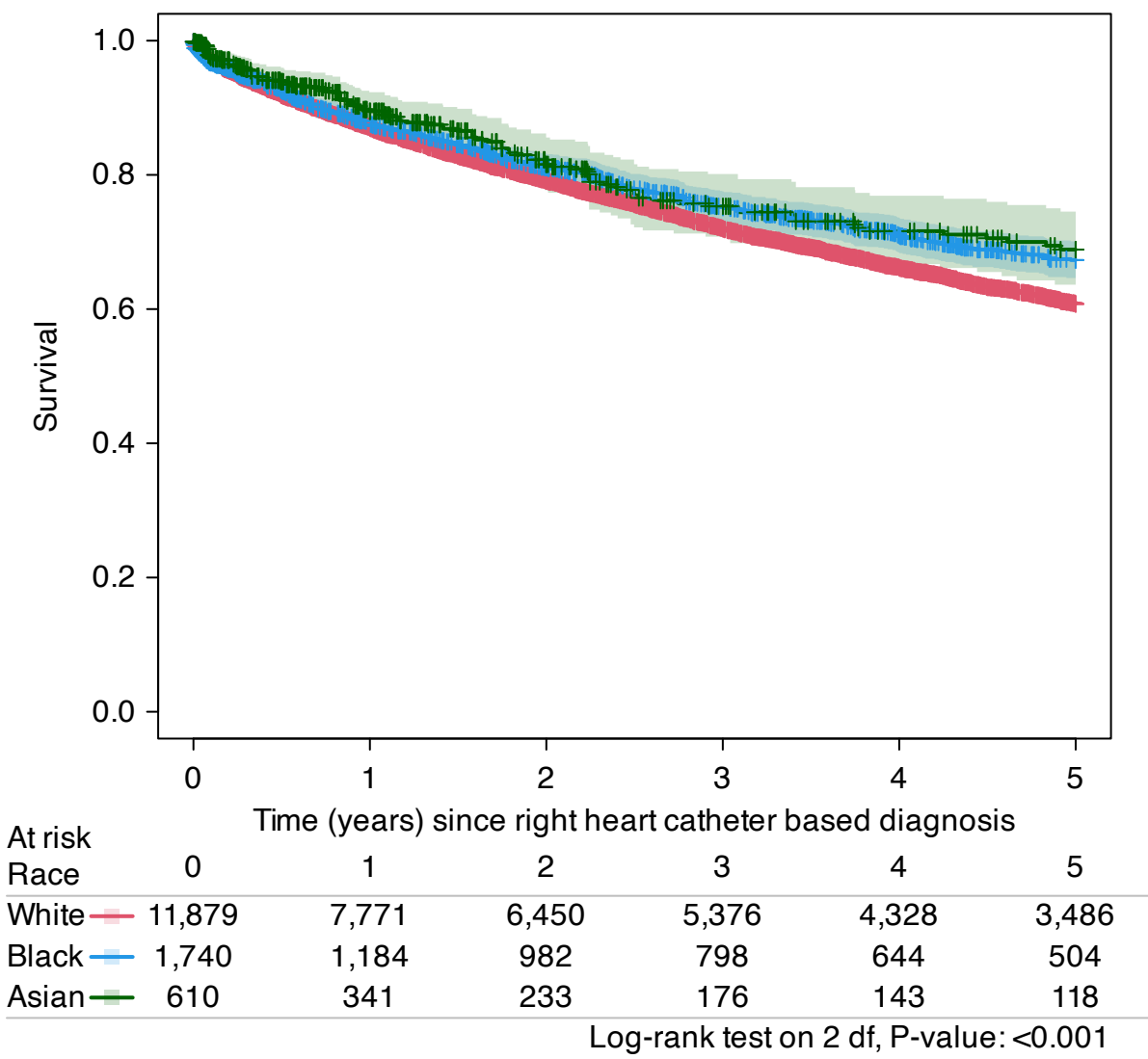

B)

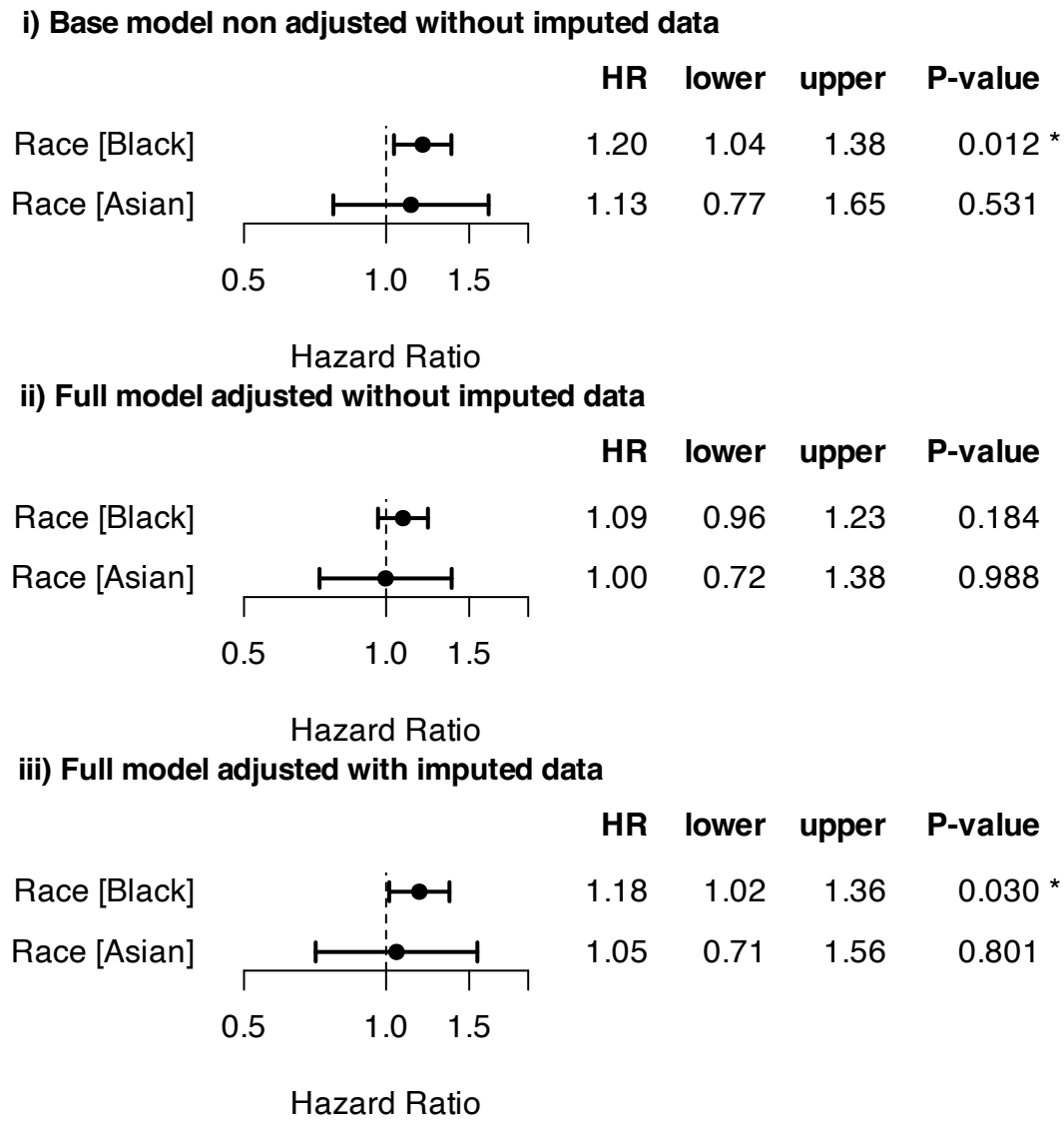

C)

i)

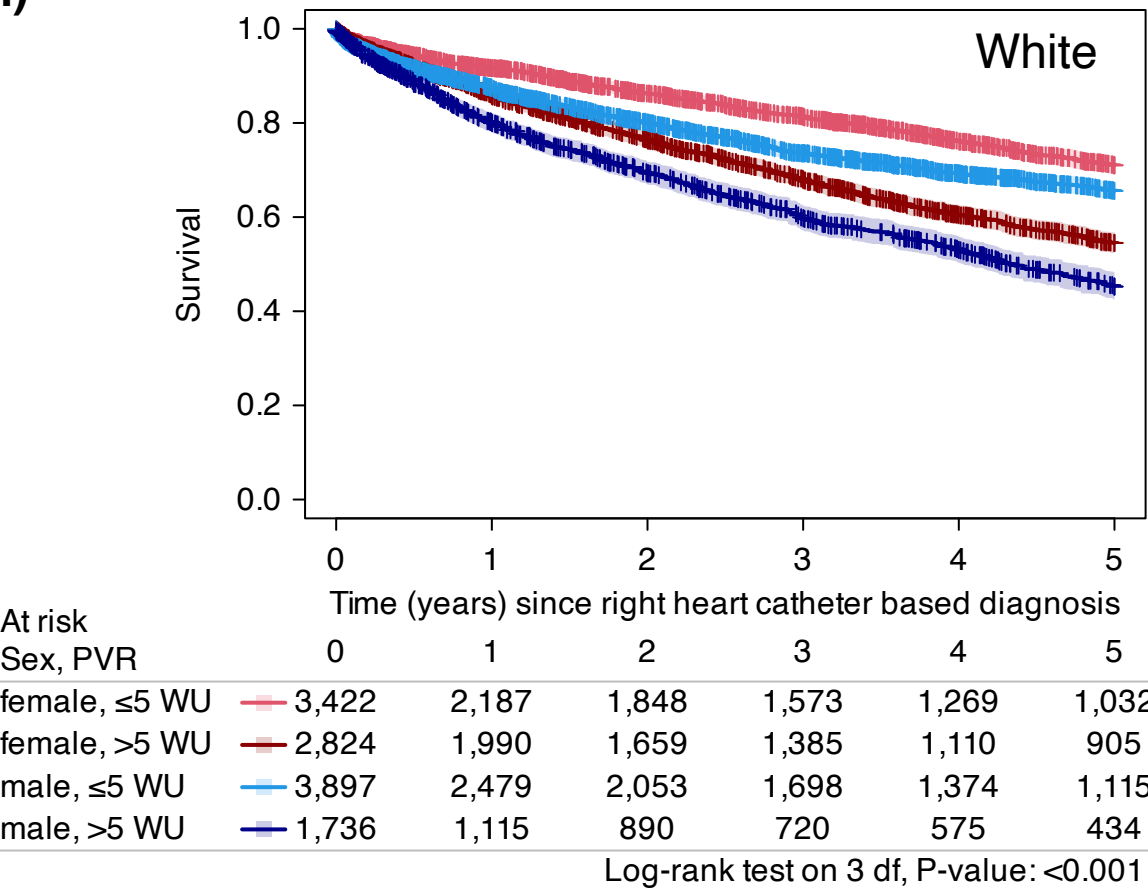

iii)

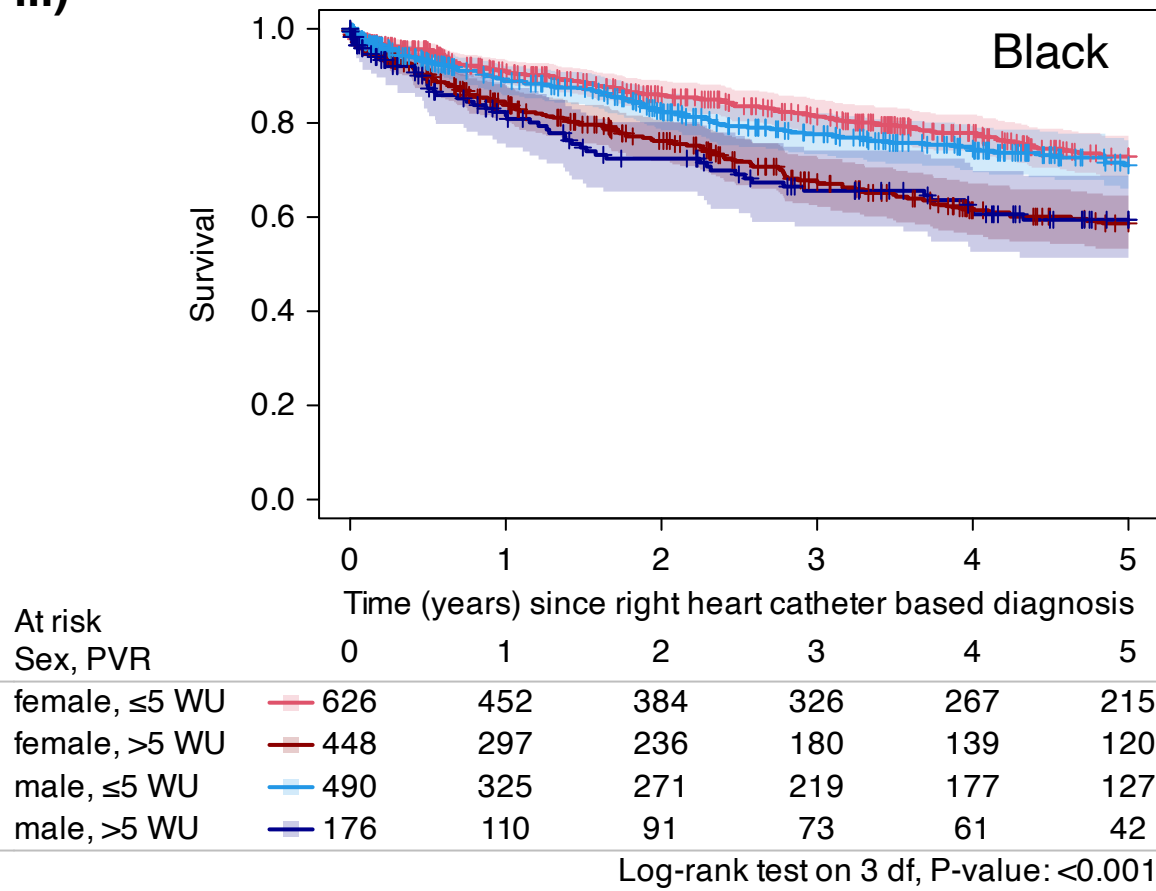

ii)

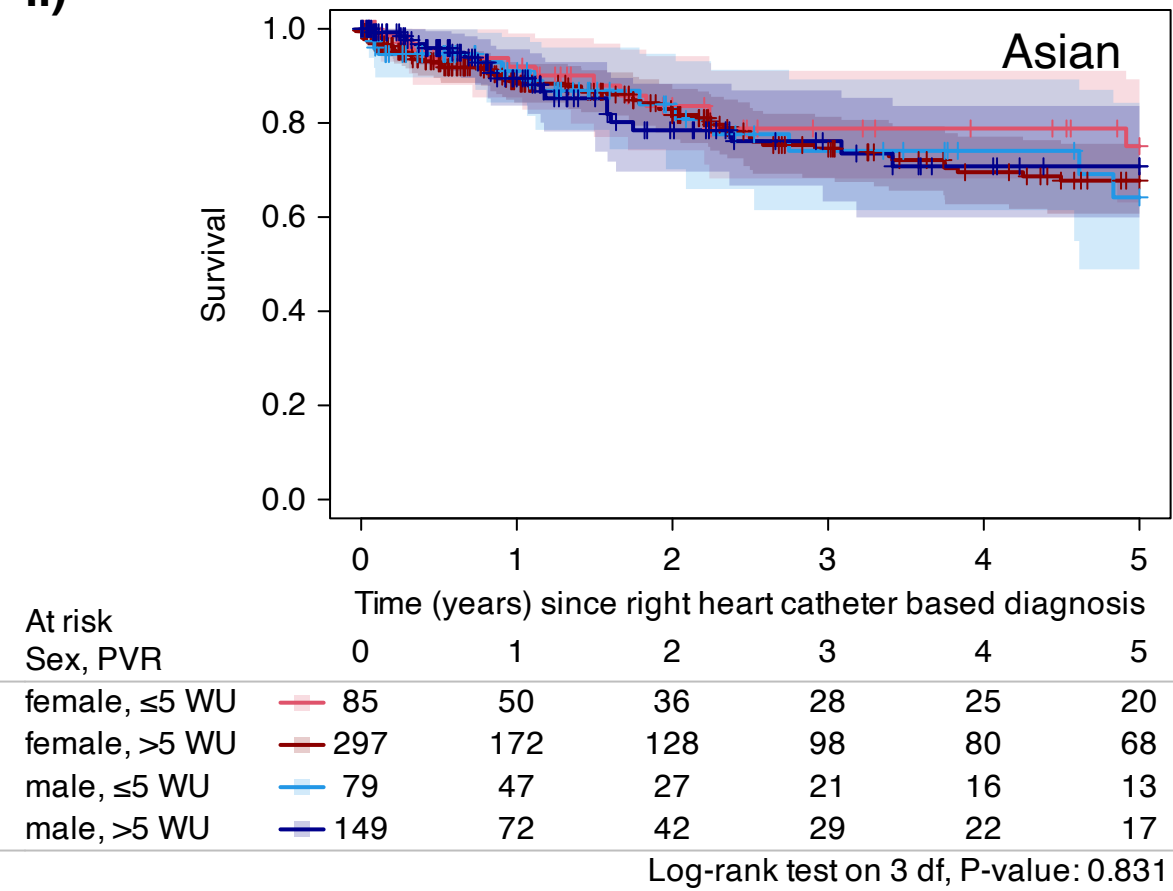

D)

| Sex   | female     | male       | Overall     |
|-------|------------|------------|-------------|
| N     | 7702       | 6527       | 14229       |
| Race  |            |            |             |
| Asian | 382 (5%)   | 228 (3.5%) | 610 (4.3%)  |
| Black | 1074 (14%) | 666 (10%)  | 1740 (12%)  |
| White | 6246 (81%) | 5633 (86%) | 11879 (83%) |
| Other | 0 (0%)     | 0 (0%)     | 0 (0%)      |

E)

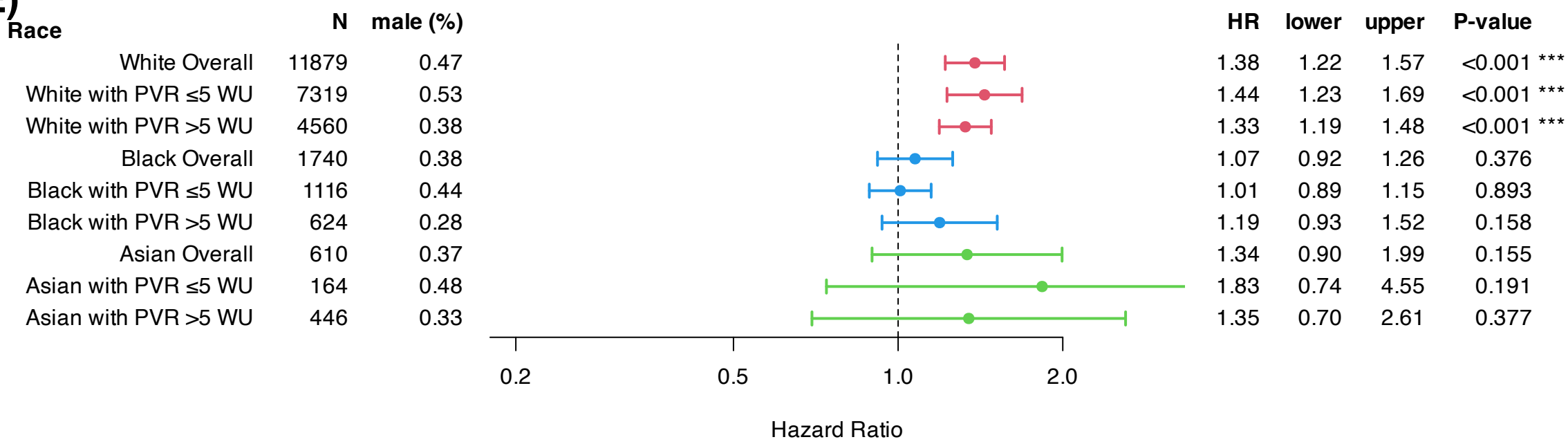

Supplement: Figure E7 [file mmc7.pdf]
