# Supplementary material for: Male survival disadvantage in pulmonary hypertension: independent of aetiology, age, disease severity, comorbidities and treatment
Source: eBioMedicine. 2025 Dec 16;123:106063. doi: 10.1016/j.ebiom.2025.106063 (PMC12768861; doi:10.1016/j.ebiom.2025.106063)
Supplement: Figure E5 [file mmc5.pdf]

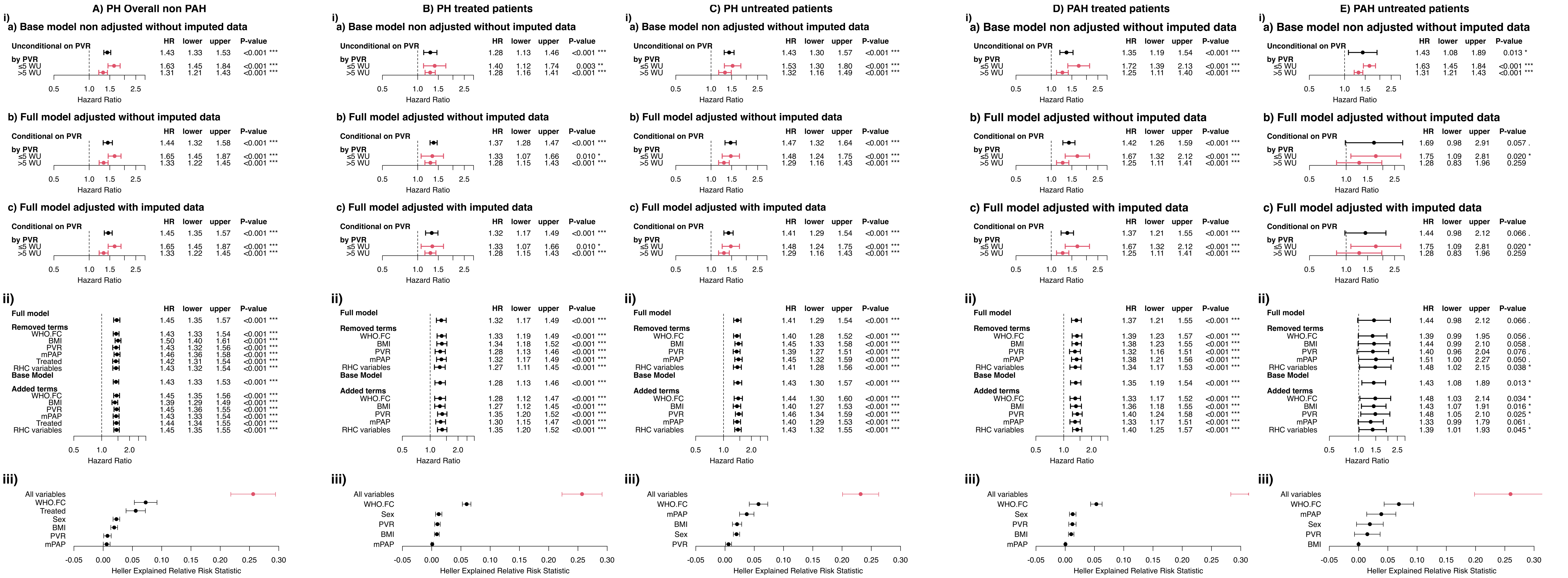
A) PH Overall non PAH
i)
a) Base model non adjusted without imputed data
HR lower upper P-value
Unconditional on PVR 1.43 1.33 1.53 <0.001 ***
by PVR
≤5 WU 1.63 1.45 1.84 <0.001 ***
>5 WU 1.31 1.21 1.43 <0.001 ***
0.5 1.0 1.5 2.5
Hazard Ratio
b) Full model adjusted without imputed data
HR lower upper P-value
Conditional on PVR 1.44 1.32 1.58 <0.001 ***
by PVR
≤5 WU 1.65 1.45 1.87 <0.001 ***
>5 WU 1.33 1.22 1.45 <0.001 ***
0.5 1.0 1.5 2.5
Hazard Ratio
c) Full model adjusted with imputed data
HR lower upper P-value
Conditional on PVR 1.45 1.35 1.57 <0.001 ***
by PVR
≤5 WU 1.65 1.45 1.87 <0.001 ***
>5 WU 1.33 1.22 1.45 <0.001 ***
0.5 1.0 1.5 2.5
Hazard Ratio
ii)
HR lower upper P-value
Full model 1.45 1.35 1.57 <0.001 ***
Removed terms
WHO.FC 1.43 1.33 1.54 <0.001 ***
BMI 1.50 1.40 1.61 <0.001 ***
PVR 1.43 1.32 1.56 <0.001 ***
mPAP 1.46 1.36 1.58 <0.001 ***
Treated 1.42 1.31 1.54 <0.001 ***
RHC variables 1.43 1.32 1.54 <0.001 ***
Base Model 1.43 1.33 1.53 <0.001 ***
Added terms
WHO.FC 1.45 1.35 1.56 <0.001 ***
BMI 1.39 1.29 1.49 <0.001 ***
PVR 1.45 1.36 1.55 <0.001 ***
mPAP 1.43 1.33 1.54 <0.001 ***
Treated 1.44 1.34 1.55 <0.001 ***
RHC variables 1.45 1.35 1.55 <0.001 ***
0.5 1.0 2.0
Hazard Ratio
iii)
All variables
WHO.FC
Treated
Sex
BMI
PVR
mPAP
-0.05 0.00 0.05 0.10 0.15 0.20 0.25 0.30
Heller Explained Relative Risk Statistic
B) PH treated patients
i)
a) Base model non adjusted without imputed data
HR lower upper P-value
Unconditional on PVR 1.28 1.13 1.46 <0.001 ***
by PVR
≤5 WU 1.40 1.12 1.74 0.003 **
>5 WU 1.28 1.16 1.41 <0.001 ***
0.5 1.0 1.5 2.5
Hazard Ratio
b) Full model adjusted without imputed data
HR lower upper P-value
Conditional on PVR 1.37 1.28 1.47 <0.001 ***
by PVR
≤5 WU 1.33 1.07 1.66 0.010 *
>5 WU 1.28 1.15 1.43 <0.001 ***
0.5 1.0 1.5 2.5
Hazard Ratio
c) Full model adjusted with imputed data
HR lower upper P-value
Conditional on PVR 1.32 1.17 1.49 <0.001 ***
by PVR
≤5 WU 1.33 1.07 1.66 0.010 *
>5 WU 1.28 1.15 1.43 <0.001 ***
0.5 1.0 1.5 2.5
Hazard Ratio
ii)
HR lower upper P-value
Full model 1.32 1.17 1.49 <0.001 ***
Removed terms
WHO.FC 1.33 1.19 1.49 <0.001 ***
BMI 1.34 1.18 1.52 <0.001 ***
PVR 1.28 1.13 1.46 <0.001 ***
mPAP 1.32 1.17 1.49 <0.001 ***
RHC variables 1.27 1.11 1.45 <0.001 ***
Base Model 1.28 1.13 1.46 <0.001 ***
Added terms
WHO.FC 1.28 1.12 1.47 <0.001 ***
BMI 1.27 1.12 1.45 <0.001 ***
PVR 1.35 1.20 1.52 <0.001 ***
mPAP 1.30 1.15 1.47 <0.001 ***
RHC variables 1.35 1.20 1.52 <0.001 ***
0.5 1.0 2.0
Hazard Ratio
iii)
All variables
WHO.FC
Sex
PVR
BMI
mPAP
-0.05 0.00 0.05 0.10 0.15 0.20 0.25 0.30
Heller Explained Relative Risk Statistic
C) PH untreated patients
i)
a) Base model non adjusted without imputed data
HR lower upper P-value
Unconditional on PVR 1.43 1.30 1.57 <0.001 ***
by PVR
≤5 WU 1.53 1.30 1.80 <0.001 ***
>5 WU 1.32 1.16 1.49 <0.001 ***
0.5 1.0 1.5 2.5
Hazard Ratio
b) Full model adjusted without imputed data
HR lower upper P-value
Conditional on PVR 1.47 1.32 1.64 <0.001 ***
by PVR
≤5 WU 1.48 1.24 1.75 <0.001 ***
>5 WU 1.29 1.16 1.43 <0.001 ***
0.5 1.0 1.5 2.5
Hazard Ratio
c) Full model adjusted with imputed data
HR lower upper P-value
Conditional on PVR 1.41 1.29 1.54 <0.001 ***
by PVR
≤5 WU 1.48 1.24 1.75 <0.001 ***
>5 WU 1.29 1.16 1.43 <0.001 ***
0.5 1.0 1.5 2.5
Hazard Ratio
ii)
HR lower upper P-value
Full model 1.41 1.29 1.54 <0.001 ***
Removed terms
WHO.FC 1.40 1.28 1.52 <0.001 ***
BMI 1.45 1.33 1.58 <0.001 ***
PVR 1.39 1.27 1.51 <0.001 ***
mPAP 1.45 1.32 1.59 <0.001 ***
RHC variables 1.41 1.28 1.56 <0.001 ***
Base Model 1.43 1.30 1.57 <0.001 ***
Added terms
WHO.FC 1.44 1.30 1.60 <0.001 ***
BMI 1.40 1.27 1.53 <0.001 ***
PVR 1.46 1.34 1.59 <0.001 ***
mPAP 1.40 1.29 1.53 <0.001 ***
RHC variables 1.43 1.32 1.55 <0.001 ***
0.5 1.0 2.0
Hazard Ratio
iii)
All variables
WHO.FC
mPAP
BMI
Sex
PVR
-0.05 0.00 0.05 0.10 0.15 0.20 0.25 0.30
Heller Explained Relative Risk Statistic
D) PAH treated patients
i)
a) Base model non adjusted without imputed data
HR lower upper P-value
Unconditional on PVR 1.35 1.19 1.54 <0.001 ***
by PVR
≤5 WU 1.72 1.39 2.13 <0.001 ***
>5 WU 1.25 1.11 1.40 <0.001 ***
0.5 1.0 1.5 2.5
Hazard Ratio
b) Full model adjusted without imputed data
HR lower upper P-value
Conditional on PVR 1.42 1.26 1.59 <0.001 ***
by PVR
≤5 WU 1.67 1.32 2.12 <0.001 ***
>5 WU 1.25 1.11 1.41 <0.001 ***
0.5 1.0 1.5 2.5
Hazard Ratio
c) Full model adjusted with imputed data
HR lower upper P-value
Conditional on PVR 1.37 1.21 1.55 <0.001 ***
by PVR
≤5 WU 1.67 1.32 2.12 <0.001 ***
>5 WU 1.25 1.11 1.41 <0.001 ***
0.5 1.0 1.5 2.5
Hazard Ratio
ii)
HR lower upper P-value
Full model 1.37 1.21 1.55 <0.001 ***
Removed terms
WHO.FC 1.39 1.23 1.57 <0.001 ***
BMI 1.38 1.23 1.55 <0.001 ***
PVR 1.32 1.16 1.51 <0.001 ***
mPAP 1.38 1.21 1.56 <0.001 ***
RHC variables 1.34 1.17 1.53 <0.001 ***
Base Model 1.35 1.19 1.54 <0.001 ***
Added terms
WHO.FC 1.33 1.17 1.52 <0.001 ***
BMI 1.36 1.18 1.55 <0.001 ***
PVR 1.40 1.24 1.58 <0.001 ***
mPAP 1.33 1.17 1.51 <0.001 ***
RHC variables 1.40 1.25 1.57 <0.001 ***
0.5 1.0 2.0
Hazard Ratio
iii)
All variables
WHO.FC
Sex
PVR
BMI
mPAP
-0.05 0.00 0.05 0.10 0.15 0.20 0.25 0.30
Heller Explained Relative Risk Statistic
E) PAH untreated patients
i)
a) Base model non adjusted without imputed data
HR lower upper P-value
Unconditional on PVR 1.43 1.08 1.89 0.013 *
by PVR
≤5 WU 1.63 1.45 1.84 <0.001 ***
>5 WU 1.31 1.21 1.43 <0.001 ***
0.5 1.0 1.5 2.5
Hazard Ratio
b) Full model adjusted without imputed data
HR lower upper P-value
Conditional on PVR 1.69 0.98 2.91 0.057 .
by PVR
≤5 WU 1.75 1.09 2.81 0.020 *
>5 WU 1.28 0.83 1.96 0.259
0.5 1.0 1.5 2.5
Hazard Ratio
c) Full model adjusted with imputed data
HR lower upper P-value
Conditional on PVR 1.44 0.98 2.12 0.066 .
by PVR
≤5 WU 1.75 1.09 2.81 0.020 *
>5 WU 1.28 0.83 1.96 0.259
0.5 1.0 1.5 2.5
Hazard Ratio
ii)
HR lower upper P-value
Full model 1.44 0.98 2.12 0.066 .
Removed terms
WHO.FC 1.39 0.99 1.95 0.056 .
BMI 1.44 0.99 2.10 0.058 .
PVR 1.40 0.96 2.04 0.076 .
mPAP 1.51 1.00 2.27 0.050 .
RHC variables 1.48 1.02 2.15 0.038 *
Base Model 1.43 1.08 1.89 0.013 *
Added terms
WHO.FC 1.48 1.03 2.14 0.034 *
BMI 1.43 1.07 1.91 0.016 *
PVR 1.48 1.05 2.10 0.025 *
mPAP 1.33 0.99 1.79 0.061 .
RHC variables 1.39 1.01 1.93 0.045 *
0.5 1.0 1.5 2.5
Hazard Ratio
iii)
All variables
WHO.FC
mPAP
Sex
PVR
BMI
-0.05 0.00 0.05 0.10 0.15 0.20 0.25 0.30
Heller Explained Relative Risk Statistic
