# Supplementary material for: Male survival disadvantage in pulmonary hypertension: independent of aetiology, age, disease severity, comorbidities and treatment
Source: eBioMedicine. 2025 Dec 16;123:106063. doi: 10.1016/j.ebiom.2025.106063 (PMC12768861; doi:10.1016/j.ebiom.2025.106063)
Supplement: Supplementary Figure captions and Tables [file mmc8.docx]

**Supplementary data**

**Figure E1. Patient Flow Chart.**

PVRI = pulmonary vascular research institute; PH = pulmonary hypertension; mPAP = mean pulmonary arterial pressure; PAWP = pulmonary arterial wedge pressure; PVR = pulmonary vascular resistance; CO = cardiac output; PAH = pulmonary arterial hypertension.

# Figure E2. Quality check for imputation procedure.

**A)** Sanity check of the imputation. The diagrams show the empirical cumulative distributions of the variables for which values were imputed. The black line and grey bar show the distribution of the original data; the red line and red bar show the distributions of the variables including the imputed values. **B)** Proportions of missing values in variables. The bars indicate the proportions of missing values separated by sex.

# Figure E3. Distribution of mean pulmonary arterial pressure

Histograms showing the distributions of mPAP at diagnosis for PH patients (A) and PAH patients (B). All mPAP values greater than 60 were summarized into the respective rightmost bar.

mPAP = mean pulmonary arterial pressure; PH = pulmonary hypertension; PAH = pulmonary arterial hypertension.

# Figure E4. Sex differences in survival in all PH patients.

Subpanel i) shows the Kaplan-Meier survival analysis with 95% confidence bands by sex. Subpanel ii) presents hazard ratios comparing men to women, based on the base-model using unimputed data. The model is only adjusted for center and diagnosis decade as strata and age, as natural spline with 2 degrees of freedom.

# Figure E5. Hazard Ratios and Sensitivity Analysis for Sex Differences in Survival, separating Patients receiving or non-receiving PH-specific Treatment.

Subpanel i) displays hazard ratios comparing men to women. Subpanel ii) shows results from a sensitivity analysis, and subpanel iii) illustrates the Heller Explained Relative Risk within the PH cohort. A) Overall non-PAH PH study population. B) Treated patients with pulmonary hypertension (PH). C) Untreated patients with PH. D) Treated patients with pulmonary arterial hypertension (PAH). E) Untreated patients with PAH. The Cox PH models were calculated for the **a)** base-model using unimputed data, including the interaction term of the dichotomized PVR and Sex. The model is only adjusted for center and diagnosis decade as strata and age, as natural spline with 2 degrees of freedom. **b)** The full model similar to a). The model is additionally adjusted for WHO FC, BMI, PVR, mPAP and PH treatment. **c)** The full model similar to b) but using the imputed data set. The plots show the hazard ratios for men compared to women.

# Figure E6. Hazard Ratios for Sex Differences in Survival for PH groups 2 – 4 and the mixed/undefined group

The Cox PH models are adjusted for center and diagnosis decade as strata and age, as natural spline with 2 degrees of freedom, as well as, WHO FC, BMI, PVR, mPAP and PH treatment. The plots show the hazard ratios for men compared to women.

PH = pulmonary hypertension; PAH = pulmonary arterial hypertension; WHO FC = WHO functional class; BMI = body mass index; PVR = pulmonary vascular resistance; mPAP = mean pulmonary arterial pressure; WU = wood units; HR = hazard ratio; lower / upper = lower and upper limits of the 95% confidence interval of the HR.

**Figure E7.** **Influence of Race Background on Survival and on Sex-Associated Survival Differences of PH patients.**

(A) Kaplan-Meier Survival curves stratified by race backgrounds. (B) Corresponding hazard ratios for race differences in survival compared to White, for i) Base model only adjusted for center and diagnosis decade as strata and age, as natural spline with 2 degrees of freedom, ii) Full model without imputed data additionally adjusted for WHO FC, BMI, PVR, mPAP and PH treatment and iii) Full model adjusted with imputed data. (C) Kaplan-Meier Survival curves stratified by sex and PVR backgrounds for i) White, ii) Black, iii) Asian and iv) Combined non-White races. (D) Contingency table. (E) Corresponding hazard ratios for sex differences in survival, comparing men to women, for subgroups of White, Black and Asian. The Cox PH models were calculated using the imputed data set and are adjusted for WHO FC, BMI, PVR, mPAP and PH treatment, as well as center and diagnosis decade as strata and age, as natural spline with 2 degrees of freedom.

PH = pulmonary hypertension; PAH = pulmonary arterial hypertension; WHO FC = WHO functional class; BMI = body mass index; PVR = pulmonary vascular resistance; mPAP = mean pulmonary arterial pressure; WU = wood units; HR = hazard ratio; lower / upper = lower and upper limits of the 95% confidence interval of the HR.

# Table E1: Baseline characteristics of PAH patients stratified by PH severity and sex.

| **Sex** | **female** | | **male** | | **Overall** |
| --- | --- | --- | --- | --- | --- |
| **PVR** | **≤5 WU** | **>5 WU** | **≤5 WU** | **>5 WU** |  |
| N | 837 | 2932 | 345 | 1260 | 5374 |
| **Age at diagnosis (years)** | | | | | |
| Median [Q1, Q3] | 64 [53, 72] | 57 [43, 69] | 61 [51, 73] | 61 [49, 71] | 60 [47, 70] |
| Missing | 0 (0%) | 0 (0%) | 0 (0%) | 0 (0%) | 0 (0%) |
| **WHO FC** | | | | | |
| I | 24 (3.7%) | 46 (2%) | 10 (3.9%) | 16 (1.6%) | 96 (2.3%) |
| II | 214 (33%) | 340 (15%) | 66 (26%) | 172 (17%) | 792 (19%) |
| III | 384 (59%) | 1595 (68%) | 166 (65%) | 680 (66%) | 2825 (66%) |
| IV | 30 (4.6%) | 352 (15%) | 12 (4.7%) | 156 (15%) | 550 (13%) |
| Missing | 185 (22%) | 599 (20%) | 91 (26%) | 236 (19%) | 1111 (21%) |
| **BMI (kg/m²)** | | | | | |
| Median [Q1, Q3] | 27 [23, 32] | 27 [23, 31] | 28 [25, 31] | 27 [24, 31] | 27 [23, 31] |
| Missing | 68 (8.1%) | 304 (10%) | 26 (7.5%) | 83 (6.6%) | 481 (9%) |
| **BSA (m²)** | | | | | |
| Median [Q1, Q3] | 1.8 [1.6, 1.9] | 1.7 [1.6, 1.9] | 2 [1.9, 2.2] | 2 [1.8, 2.1] | 1.8 [1.6, 2] |
| Missing | 27 (3.2%) | 118 (4%) | 6 (1.7%) | 29 (2.3%) | 180 (3.3%) |
| **Height (cm)** | | | | | |
| Median [Q1, Q3] | 160 [160, 170] | 160 [160, 170] | 180 [170, 180] | 170 [170, 180] | 160 [160, 170] |
| Missing | 79 (9.4%) | 366 (12%) | 31 (9%) | 107 (8.5%) | 583 (11%) |
| **Weight (kg)** | | | | | |
| Median [Q1, Q3] | 70 [61, 85] | 68 [58, 82] | 85 [74, 97] | 81 [71, 94] | 74 [62, 87] |
| Missing | 87 (10%) | 387 (13%) | 34 (9.9%) | 112 (8.9%) | 620 (12%) |
| **6MWD (m/6min)** | | | | | |
| Median [Q1, Q3] | 330 [240, 420] | 290 [190, 380] | 350 [250, 450] | 300 [200, 410] | 300 [200, 400] |
| Missing | 319 (38%) | 1093 (37%) | 142 (41%) | 445 (35%) | 1999 (37%) |
| **BNP (pg/mL)** | | | | | |
| Median [Q1, Q3] | 75 [38, 170] | 230 [88, 530] | 88 [36, 190] | 240 [98, 510] | 180 [69, 440] |
| Missing | 401 (48%) | 1322 (45%) | 148 (43%) | 573 (45%) | 2444 (45%) |
| **mPAP (mmHg)** | | | | | |
| Median [Q1, Q3] | 30 [26, 34] | 50 [42, 58] | 33 [28, 38] | 49 [42, 56] | 46 [36, 55] |
| Missing | 0 (0%) | 0 (0%) | 0 (0%) | 0 (0%) | 0 (0%) |
| **sPAP (mmHg)** | | | | | |
| Median [Q1, Q3] | 48 [42, 56] | 80 [68, 94] | 53 [45, 64] | 79 [70, 90] | 75 [59, 89] |
| Missing | 67 (8%) | 214 (7.3%) | 26 (7.5%) | 117 (9.3%) | 424 (7.9%) |
| **CVP (mmHg)** | | | | | |
| Median [Q1, Q3] | 6 [4, 8] | 8 [5, 12] | 6 [4, 9] | 8 [5, 11] | 7 [5, 11] |
| Missing | 354 (42%) | 1204 (41%) | 119 (34%) | 484 (38%) | 2161 (40%) |
| **PAWP (mmHg)** | | | | | |
| Median [Q1, Q3] | 10 [8, 13] | 9 [7, 11] | 11 [8, 13] | 9 [7, 12] | 9 [7, 12] |
| Missing | 0 (0%) | 0 (0%) | 0 (0%) | 0 (0%) | 0 (0%) |
| **CO (L/min)** | | | | | |
| Median [Q1, Q3] | 5.3 [4.6, 6.4] | 3.7 [2.9, 4.5] | 6 [5.1, 7.4] | 4.2 [3.3, 5.1] | 4.2 [3.2, 5.2] |
| Missing | 0 (0%) | 0 (0%) | 0 (0%) | 0 (0%) | 0 (0%) |
| **CI (L/(min·m²))** | | | | | |
| Median [Q1, Q3] | 3 [2.6, 3.5] | 2.1 [1.7, 2.6] | 3 [2.5, 3.5] | 2.1 [1.7, 2.5] | 2.3 [1.8, 2.8] |
| Missing | 26 (3.1%) | 118 (4%) | 5 (1.4%) | 28 (2.2%) | 177 (3.3%) |
| **PVR (WU)** | | | | | |
| Median [Q1, Q3] | 3.7 [3, 4.4] | 11 [7.9, 15] | 3.8 [3.1, 4.5] | 9.2 [6.9, 12] | 8.7 [5.5, 13] |
| Missing | 0 (0%) | 0 (0%) | 0 (0%) | 0 (0%) | 0 (0%) |

# Table E2: Baseline characteristics of non-PAH PH patients stratified by PH severity and sex.

| **Sex** | **female** | | **male** | | **Overall** |
| --- | --- | --- | --- | --- | --- |
| **PVR** | **≤5 WU** | **>5 WU** | **≤5 WU** | **>5 WU** |  |
| N | 1863 | 2176 | 1533 | 1905 | 7477 |
| **Age at diagnosis (years)** | | | | | |
| Median [Q1, Q3] | 68 [58, 75] | 67 [56, 75] | 68 [58, 75] | 67 [58, 74] | 67 [58, 75] |
| Missing | 0 (0%) | 0 (0%) | 0 (0%) | 0 (0%) | 0 (0%) |
| **WHO FC** | | | | | |
| I | 24 (1.6%) | 13 (0.68%) | 31 (2.5%) | 28 (1.6%) | 96 (1.5%) |
| II | 277 (18%) | 205 (11%) | 265 (22%) | 252 (15%) | 999 (16%) |
| III | 1093 (72%) | 1376 (72%) | 822 (67%) | 1144 (67%) | 4435 (70%) |
| IV | 117 (7.7%) | 306 (16%) | 112 (9.1%) | 291 (17%) | 826 (13%) |
| Missing | 352 (19%) | 276 (13%) | 303 (20%) | 190 (10%) | 1121 (15%) |
| **BMI (kg/m²)** | | | | | |
| Median [Q1, Q3] | 30 [25, 36] | 27 [23, 32] | 28 [25, 33] | 26 [24, 30] | 28 [24, 33] |
| Missing | 129 (6.9%) | 125 (5.7%) | 84 (5.5%) | 114 (6%) | 452 (6%) |
| **BSA (m²)** | | | | | |
| Median [Q1, Q3] | 1.9 [1.7, 2] | 1.7 [1.6, 1.9] | 2 [1.9, 2.2] | 1.9 [1.8, 2.1] | 1.9 [1.7, 2.1] |
| Missing | 39 (2.1%) | 54 (2.5%) | 27 (1.8%) | 48 (2.5%) | 168 (2.2%) |
| **Height (cm)** | | | | | |
| Median [Q1, Q3] | 160 [160, 170] | 160 [160, 160] | 180 [170, 180] | 170 [170, 180] | 170 [160, 180] |
| Missing | 130 (7%) | 119 (5.5%) | 87 (5.7%) | 112 (5.9%) | 448 (6%) |
| **Weight (kg)** | | | | | |
| Median [Q1, Q3] | 79 [66, 95] | 70 [59, 83] | 88 [76, 100] | 80 [70, 92] | 78 [66, 93] |
| Missing | 154 (8.3%) | 144 (6.6%) | 103 (6.7%) | 129 (6.8%) | 530 (7.1%) |
| **6MWD (m/6min)** | | | | | |
| Median [Q1, Q3] | 280 [200, 360] | 250 [170, 330] | 320 [230, 410] | 280 [190, 370] | 280 [190, 370] |
| Missing | 892 (48%) | 886 (41%) | 676 (44%) | 732 (38%) | 3186 (43%) |
| **BNP (pg/mL)** | | | | | |
| Median [Q1, Q3] | 110 [44, 240] | 220 [85, 460] | 110 [38, 320] | 270 [120, 540] | 180 [64, 400] |
| Missing | 953 (51%) | 1024 (47%) | 729 (48%) | 853 (45%) | 3559 (48%) |
| **mPAP (mmHg)** | | | | | |
| Median [Q1, Q3] | 32 [28, 38] | 46 [39, 53] | 32 [27, 37] | 45 [39, 52] | 39 [31, 48] |
| Missing | 0 (0%) | 0 (0%) | 0 (0%) | 0 (0%) | 0 (0%) |
| **sPAP (mmHg)** | | | | | |
| Median [Q1, Q3] | 50 [43, 60] | 75 [64, 88] | 52 [44, 62] | 76 [64, 88] | 64 [50, 79] |
| Missing | 155 (8.3%) | 290 (13%) | 171 (11%) | 257 (13%) | 873 (12%) |
| **CVP (mmHg)** | | | | | |
| Median [Q1, Q3] | 8 [5, 12] | 8 [5, 13] | 8 [4, 12] | 8 [5, 12] | 8 [5, 12] |
| Missing | 603 (32%) | 703 (32%) | 440 (29%) | 636 (33%) | 2382 (32%) |
| **PAWP (mmHg)** | | | | | |
| Median [Q1, Q3] | 17 [12, 21] | 11 [8, 16] | 14 [10, 20] | 10 [7, 14] | 12 [9, 18] |
| Missing | 0 (0%) | 0 (0%) | 0 (0%) | 0 (0%) | 0 (0%) |
| **CO (L/min)** | | | | | |
| Median [Q1, Q3] | 5.1 [4.2, 6.2] | 3.6 [3, 4.4] | 5.5 [4.6, 6.5] | 4 [3.3, 4.8] | 4.4 [3.5, 5.4] |
| Missing | 0 (0%) | 0 (0%) | 0 (0%) | 0 (0%) | 0 (0%) |
| **CI (L/(min·m²))** | | | | | |
| Median [Q1, Q3] | 2.8 [2.3, 3.3] | 2.1 [1.7, 2.5] | 2.7 [2.3, 3.1] | 2 [1.7, 2.4] | 2.3 [1.9, 2.8] |
| Missing | 38 (2%) | 54 (2.5%) | 26 (1.7%) | 47 (2.5%) | 165 (2.2%) |
| **PVR (WU)** | | | | | |
| Median [Q1, Q3] | 3.2 [2.3, 4] | 8.8 [6.5, 12] | 3.2 [2.3, 4.1] | 8.3 [6.3, 11] | 5.5 [3.4, 9] |
| Missing | 0 (0%) | 0 (0%) | 0 (0%) | 0 (0%) | 0 (0%) |

**Table E3: Baseline characteristics stratified by PH group.**

| **PH Group** | **1** | **2** | **3** | **4** | **5** | **multiple/ undefined** | **Overall** |
| --- | --- | --- | --- | --- | --- | --- | --- |
| N | 5374 | 1950 | 2544 | 2528 | 455 | 8272 | 21123 |
| **Age at diagnosis (years)** | | | | | | | |
| Median [Q1, Q3] | 60 [47, 70] | 72 [64, 77] | 67 [59, 73] | 65 [52, 74] | 60 [50, 69] | 62 [52, 71] | 64 [52, 72] |
| Missing | 0 (0%) | 0 (0%) | 0 (0%) | 0 (0%) | 0 (0%) | 0 (0%) | 0 (0%) |
| **Sex** | | | | | | | |
| male | 1605 (30%) | 726 (37%) | 1335 (52%) | 1210 (48%) | 167 (37%) | 4250 (51%) | 9293 (44%) |
| Missing | 0 (0%) | 0 (0%) | 0 (0%) | 0 (0%) | 0 (0%) | 0 (0%) | 0 (0%) |
| **WHO FC** | | | | | | | |
| I | 96 (2.3%) | 28 (1.7%) | 7 (0.33%) | 51 (2.2%) | 10 (2.8%) | 256 (6.6%) | 448 (3.1%) |
| II | 792 (19%) | 240 (15%) | 267 (13%) | 429 (19%) | 63 (18%) | 959 (25%) | 2750 (19%) |
| III | 2825 (66%) | 1210 (75%) | 1395 (67%) | 1594 (69%) | 236 (67%) | 2210 (57%) | 9470 (65%) |
| IV | 550 (13%) | 138 (8.5%) | 424 (20%) | 221 (9.6%) | 43 (12%) | 444 (11%) | 1820 (13%) |
| Missing | 1111 (21%) | 334 (17%) | 451 (18%) | 233 (9.2%) | 103 (23%) | 4403 (53%) | 6635 (31%) |
| **BMI (kg/m²)** | | | | | | | |
| Median [Q1, Q3] | 27 [23, 31] | 30 [26, 35] | 27 [23, 32] | 27 [24, 32] | 27 [23, 32] | 29 [25, 35] | 28 [24, 33] |
| Missing | 481 (9%) | 189 (9.7%) | 106 (4.2%) | 134 (5.3%) | 23 (5.1%) | 326 (3.9%) | 1259 (6%) |
| **BSA (m²)** | | | | | | | |
| Median [Q1, Q3] | 1.8 [1.6, 2] | 1.9 [1.8, 2.1] | 1.9 [1.7, 2] | 1.9 [1.7, 2.1] | 1.9 [1.7, 2] | 2 [1.8, 2.2] | 1.9 [1.7, 2.1] |
| Missing | 180 (3.3%) | 83 (4.3%) | 39 (1.5%) | 32 (1.3%) | 14 (3.1%) | 71 (0.86%) | 419 (2%) |
| **Height (cm)** | | | | | | | |
| Median [Q1, Q3] | 160 [160, 170] | 160 [160, 170] | 170 [160, 180] | 170 [160, 180] | 170 [160, 170] | 170 [160, 180] | 170 [160, 180] |
| Missing | 583 (11%) | 166 (8.5%) | 118 (4.6%) | 148 (5.9%) | 16 (3.5%) | 360 (4.4%) | 1391 (6.6%) |
| **Weight (kg)** | | | | | | | |
| Median [Q1, Q3] | 74 [62, 87] | 82 [70, 97] | 77 [65, 91] | 78 [66, 92] | 76 [63, 90] | 86 [72, 100] | 80 [67, 96] |
| Missing | 620 (12%) | 214 (11%) | 130 (5.1%) | 163 (6.4%) | 23 (5.1%) | 366 (4.4%) | 1516 (7.2%) |
| **6MWD (m/6min)** | | | | | | | |
| Median [Q1, Q3] | 300 [200, 400] | 260 [180, 350] | 240 [170, 320] | 320 [220, 400] | 280 [200, 370] | 300 [200, 390] | 290 [200, 380] |
| Missing | 1999 (37%) | 1003 (51%) | 1147 (45%) | 817 (32%) | 219 (48%) | 7260 (88%) | 12445 (59%) |
| **BNP (pg/mL)** | | | | | | | |
| Median [Q1, Q3] | 180 [69, 440] | 260 [140, 470] | 130 [47, 350] | 160 [55, 360] | 180 [60, 520] | 440 [160, 1000] | 260 [93, 650] |
| Missing | 2444 (45%) | 992 (51%) | 1162 (46%) | 1129 (45%) | 276 (61%) | 3122 (38%) | 9125 (43%) |
| **mPAP (mmHg)** | | | | | | | |
| Median [Q1, Q3] | 46 [36, 55] | 38 [31, 45] | 36 [29, 45] | 42 [34, 51] | 41 [34, 49] | 32 [26, 41] | 38 [29, 48] |
| Missing | 0 (0%) | 0 (0%) | 0 (0%) | 0 (0%) | 0 (0%) | 0 (0%) | 0 (0%) |
| **sPAP (mmHg)** | | | | | | | |
| Median [Q1, Q3] | 75 [59, 89] | 60 [48, 75] | 59 [47, 74] | 72 [57, 87] | 67 [52, 79] | 48 [38, 63] | 60 [45, 78] |
| Missing | 424 (7.9%) | 153 (7.8%) | 301 (12%) | 385 (15%) | 34 (7.5%) | 89 (1.1%) | 1386 (6.6%) |
| **CVP (mmHg)** | | | | | | | |
| Median [Q1, Q3] | 7 [5, 11] | 12 [9, 16] | 6 [3, 9] | 7 [4, 11] | 9 [5, 13] | 9 [6, 13] | 8 [5, 12] |
| Missing | 2161 (40%) | 633 (32%) | 737 (29%) | 907 (36%) | 105 (23%) | 7117 (86%) | 11660 (55%) |
| **PAWP (mmHg)** | | | | | | | |
| Median [Q1, Q3] | 9 [7, 12] | 21 [19, 25] | 10 [7, 13] | 10 [8, 13] | 12 [9, 16] | 18 [13, 23] | 13 [9, 19] |
| Missing | 0 (0%) | 0 (0%) | 0 (0%) | 0 (0%) | 0 (0%) | 0 (0%) | 0 (0%) |
| **CO (L/min)** | | | | | | | |
| Median [Q1, Q3] | 4.2 [3.2, 5.2] | 4.6 [3.7, 5.8] | 4.5 [3.7, 5.5] | 4.1 [3.2, 5.1] | 4.6 [3.7, 5.8] | 4.9 [3.9, 6.2] | 4.5 [3.6, 5.7] |
| Missing | 0 (0%) | 0 (0%) | 0 (0%) | 0 (0%) | 0 (0%) | 0 (0%) | 0 (0%) |
| **CI (L/(min·m²))** | | | | | | | |
| Median [Q1, Q3] | 2.3 [1.8, 2.8] | 2.4 [2, 2.9] | 2.4 [2, 2.9] | 2.2 [1.8, 2.6] | 2.5 [2.1, 3.1] | 2.5 [2, 3.1] | 2.4 [1.9, 2.9] |
| Missing | 177 (3.3%) | 80 (4.1%) | 39 (1.5%) | 32 (1.3%) | 14 (3.1%) | 58 (0.7%) | 400 (1.9%) |
| **PVR (WU)** | | | | | | | |
| Median [Q1, Q3] | 8.7 [5.5, 13] | 3.3 [2.1, 5.2] | 5.6 [3.8, 8.5] | 7.8 [4.8, 11] | 5.7 [3.7, 9.1] | 2.6 [1.7, 4.4] | 4.7 [2.6, 8.7] |
| Missing | 0 (0%) | 0 (0%) | 0 (0%) | 0 (0%) | 0 (0%) | 0 (0%) | 0 (0%) |

# Table E4: Baseline characteristics of treated (A) PH and (B) PAH patients.

(A)

| **Sex** | **female** | | **male** | | **Overall** |
| --- | --- | --- | --- | --- | --- |
| **PVR** | **≤5 WU** | **>5 WU** | **≤5 WU** | **>5 WU** |  |
| N | 1832 | 4667 | 1710 | 2692 | 10901 |
| **Age at diagnosis (years)** | | | | | |
| Median [Q1, Q3] | 63 [51, 72] | 61 [48, 71] | 61 [52, 69] | 64 [53, 72] | 62 [50, 71] |
| Missing | 0 (0%) | 0 (0%) | 0 (0%) | 0 (0%) | 0 (0%) |
| **WHO FC** | | | | | |
| I | 32 (2.4%) | 52 (1.4%) | 57 (4.7%) | 36 (1.6%) | 177 (2.1%) |
| II | 359 (27%) | 467 (12%) | 250 (20%) | 327 (14%) | 1403 (16%) |
| III | 837 (63%) | 2645 (70%) | 773 (63%) | 1556 (68%) | 5811 (67%) |
| IV | 111 (8.3%) | 589 (16%) | 142 (12%) | 386 (17%) | 1228 (14%) |
| Missing | 493 (27%) | 914 (20%) | 488 (29%) | 387 (14%) | 2282 (21%) |
| **BMI (kg/m²)** | | | | | |
| Median [Q1, Q3] | 29 [24, 35] | 27 [23, 32] | 29 [25, 33] | 27 [24, 31] | 28 [24, 32] |
| Missing | 126 (6.9%) | 406 (8.7%) | 61 (3.6%) | 163 (6.1%) | 756 (6.9%) |
| **BSA (m²)** | | | | | |
| Median [Q1, Q3] | 1.8 [1.7, 2] | 1.7 [1.6, 1.9] | 2.1 [1.9, 2.2] | 2 [1.8, 2.1] | 1.9 [1.7, 2] |
| Missing | 37 (2%) | 140 (3%) | 13 (0.76%) | 50 (1.9%) | 240 (2.2%) |
| **Height (cm)** | | | | | |
| Median [Q1, Q3] | 160 [160, 170] | 160 [160, 170] | 180 [170, 180] | 170 [170, 180] | 170 [160, 180] |
| Missing | 143 (7.8%) | 477 (10%) | 77 (4.5%) | 207 (7.7%) | 904 (8.3%) |
| **Weight (kg)** | | | | | |
| Median [Q1, Q3] | 76 [64, 91] | 70 [59, 84] | 90 [78, 100] | 82 [71, 94] | 78 [65, 92] |
| Missing | 155 (8.5%) | 511 (11%) | 82 (4.8%) | 217 (8.1%) | 965 (8.9%) |
| **6MWD (m/6min)** | | | | | |
| Median [Q1, Q3] | 310 [220, 400] | 270 [190, 360] | 340 [240, 420] | 280 [190, 380] | 290 [200, 380] |
| Missing | 922 (50%) | 1792 (38%) | 1117 (65%) | 1012 (38%) | 4843 (44%) |
| **BNP (pg/mL)** | | | | | |
| Median [Q1, Q3] | 120 [47, 370] | 250 [95, 550] | 240 [72, 690] | 270 [120, 590] | 230 [83, 560] |
| Missing | 702 (38%) | 1933 (41%) | 549 (32%) | 1089 (40%) | 4273 (39%) |
| **mPAP (mmHg)** | | | | | |
| Median [Q1, Q3] | 31 [27, 38] | 49 [42, 57] | 32 [27, 38] | 48 [41, 55] | 43 [34, 52] |
| Missing | 0 (0%) | 0 (0%) | 0 (0%) | 0 (0%) | 0 (0%) |
| **sPAP (mmHg)** | | | | | |
| Median [Q1, Q3] | 49 [42, 60] | 80 [68, 94] | 50 [40, 60] | 78 [68, 90] | 70 [54, 85] |
| Missing | 77 (4.2%) | 417 (8.9%) | 64 (3.7%) | 289 (11%) | 847 (7.8%) |
| **CVP (mmHg)** | | | | | |
| Median [Q1, Q3] | 7 [4, 9] | 8 [5, 12] | 7 [4, 10] | 8 [5, 12] | 8 [5, 12] |
| Missing | 995 (54%) | 2072 (44%) | 1104 (65%) | 1189 (44%) | 5360 (49%) |
| **PAWP (mmHg)** | | | | | |
| Median [Q1, Q3] | 13 [10, 17] | 10 [7, 13] | 14 [10, 21] | 10 [7, 13] | 11 [8, 15] |
| Missing | 0 (0%) | 0 (0%) | 0 (0%) | 0 (0%) | 0 (0%) |
| **CO (L/min)** | | | | | |
| Median [Q1, Q3] | 5.3 [4.4, 6.4] | 3.7 [3, 4.5] | 5.5 [4.5, 6.7] | 4.1 [3.3, 4.9] | 4.3 [3.4, 5.3] |
| Missing | 0 (0%) | 0 (0%) | 0 (0%) | 0 (0%) | 0 (0%) |
| **CI (L/(min·m²))** | | | | | |
| Median [Q1, Q3] | 2.9 [2.4, 3.5] | 2.1 [1.7, 2.5] | 2.6 [2.2, 3.2] | 2 [1.7, 2.4] | 2.3 [1.8, 2.8] |
| Missing | 36 (2%) | 139 (3%) | 12 (0.7%) | 49 (1.8%) | 236 (2.2%) |
| **PVR (WU)** | | | | | |
| Median [Q1, Q3] | 3.5 [2.6, 4.2] | 10 [7.3, 14] | 3 [2, 4] | 8.7 [6.6, 12] | 7.1 [4.2, 11] |
| Missing | 0 (0%) | 0 (0%) | 0 (0%) | 0 (0%) | 0 (0%) |

(B)

| **Sex** | **female** | | **male** | | **Overall** |
| --- | --- | --- | --- | --- | --- |
| **PVR** | **≤5 WU** | **>5 WU** | **≤5 WU** | **>5 WU** |  |
| N | 655 | 2560 | 254 | 1087 | 4556 |
| **Age at diagnosis (years)** | | | | | |
| Median [Q1, Q3] | 65 [53, 72] | 57 [43, 69] | 64 [55, 73] | 61 [49, 71] | 60 [47, 70] |
| Missing | 0 (0%) | 0 (0%) | 0 (0%) | 0 (0%) | 0 (0%) |
| **WHO FC** | | | | | |
| I | 13 (2.5%) | 37 (1.8%) | 6 (2.9%) | 12 (1.3%) | 68 (1.8%) |
| II | 175 (33%) | 279 (13%) | 48 (23%) | 144 (15%) | 646 (17%) |
| III | 313 (59%) | 1454 (70%) | 143 (69%) | 629 (68%) | 2539 (68%) |
| IV | 27 (5.1%) | 308 (15%) | 11 (5.3%) | 146 (16%) | 492 (13%) |
| Missing | 127 (19%) | 482 (19%) | 46 (18%) | 156 (14%) | 811 (18%) |
| **BMI (kg/m²)** | | | | | |
| Median [Q1, Q3] | 27 [23, 32] | 27 [23, 32] | 28 [25, 31] | 27 [24, 31] | 27 [23, 32] |
| Missing | 57 (8.7%) | 281 (11%) | 17 (6.7%) | 66 (6.1%) | 421 (9.2%) |
| **BSA (m²)** | | | | | |
| Median [Q1, Q3] | 1.8 [1.6, 1.9] | 1.7 [1.6, 1.9] | 2 [1.9, 2.2] | 2 [1.8, 2.1] | 1.8 [1.7, 2] |
| Missing | 26 (4%) | 114 (4.5%) | 5 (2%) | 25 (2.3%) | 170 (3.7%) |
| **Height (cm)** | | | | | |
| Median [Q1, Q3] | 160 [160, 170] | 160 [160, 170] | 180 [170, 180] | 170 [170, 180] | 160 [160, 170] |
| Missing | 64 (9.8%) | 337 (13%) | 21 (8.3%) | 93 (8.6%) | 515 (11%) |
| **Weight (kg)** | | | | | |
| Median [Q1, Q3] | 70 [60, 85] | 69 [59, 82] | 86 [76, 98] | 82 [71, 94] | 74 [62, 87] |
| Missing | 72 (11%) | 358 (14%) | 23 (9.1%) | 96 (8.8%) | 549 (12%) |
| **6MWD (m/6min)** | | | | | |
| Median [Q1, Q3] | 330 [240, 420] | 290 [190, 370] | 360 [250, 450] | 300 [200, 400] | 300 [200, 390] |
| Missing | 233 (36%) | 899 (35%) | 88 (35%) | 345 (32%) | 1565 (34%) |
| **BNP (pg/mL)** | | | | | |
| Median [Q1, Q3] | 73 [37, 170] | 240 [93, 530] | 86 [37, 190] | 240 [97, 510] | 190 [71, 450] |
| Missing | 296 (45%) | 1098 (43%) | 93 (37%) | 474 (44%) | 1961 (43%) |
| **mPAP (mmHg)** | | | | | |
| Median [Q1, Q3] | 30 [27, 35] | 50 [43, 58] | 32 [29, 38] | 49 [43, 56] | 46 [37, 55] |
| Missing | 0 (0%) | 0 (0%) | 0 (0%) | 0 (0%) | 0 (0%) |
| **sPAP (mmHg)** | | | | | |
| Median [Q1, Q3] | 48 [42, 57] | 81 [69, 95] | 53 [45, 64] | 80 [70, 90] | 76 [61, 90] |
| Missing | 40 (6.1%) | 183 (7.1%) | 15 (5.9%) | 103 (9.5%) | 341 (7.5%) |
| **CVP (mmHg)** | | | | | |
| Median [Q1, Q3] | 6 [4, 8] | 8 [5, 12] | 6 [4, 9] | 8 [5, 12] | 8 [5, 11] |
| Missing | 319 (49%) | 1144 (45%) | 102 (40%) | 455 (42%) | 2020 (44%) |
| **PAWP (mmHg)** | | | | | |
| Median [Q1, Q3] | 10 [8, 13] | 9 [7, 11] | 11 [8.2, 13] | 9 [7, 12] | 10 [7, 12] |
| Missing | 0 (0%) | 0 (0%) | 0 (0%) | 0 (0%) | 0 (0%) |
| **CO (L/min)** | | | | | |
| Median [Q1, Q3] | 5.4 [4.6, 6.5] | 3.7 [2.9, 4.5] | 5.9 [5.1, 7.1] | 4.2 [3.3, 5.1] | 4.1 [3.2, 5.2] |
| Missing | 0 (0%) | 0 (0%) | 0 (0%) | 0 (0%) | 0 (0%) |
| **CI (L/(min·m²))** | | | | | |
| Median [Q1, Q3] | 3 [2.6, 3.5] | 2.1 [1.7, 2.6] | 2.9 [2.4, 3.4] | 2.1 [1.7, 2.5] | 2.3 [1.8, 2.8] |
| Missing | 25 (3.8%) | 114 (4.5%) | 4 (1.6%) | 25 (2.3%) | 168 (3.7%) |
| **PVR (WU)** | | | | | |
| Median [Q1, Q3] | 3.8 [3.1, 4.4] | 11 [8, 16] | 3.8 [3.2, 4.5] | 9.3 [6.9, 13] | 9 [5.7, 13] |
| Missing | 0 (0%) | 0 (0%) | 0 (0%) | 0 (0%) | 0 (0%) |

**Table E5: Baseline characteristics of non-treated (A) PH and (B) PAH patients.**

(A)

| **Sex** | **female** | | **male** | | **Overall** |
| --- | --- | --- | --- | --- | --- |
| **PVR** | **≤5 WU** | **>5 WU** | **≤5 WU** | **>5 WU** |  |
| N | 3819 | 1512 | 3792 | 1099 | 10222 |
| **Age at diagnosis (years)** | | | | | |
| Median [Q1, Q3] | 65 [55, 74] | 65 [53, 74] | 65 [55, 74] | 66 [55, 73] | 65 [55, 74] |
| Missing | 0 (0%) | 0 (0%) | 0 (0%) | 0 (0%) | 0 (0%) |
| **WHO FC** | | | | | |
| I | 90 (4.5%) | 24 (2.2%) | 128 (6.6%) | 29 (3.5%) | 271 (4.6%) |
| II | 472 (24%) | 184 (17%) | 528 (27%) | 163 (20%) | 1347 (23%) |
| III | 1315 (65%) | 717 (66%) | 1120 (58%) | 507 (61%) | 3659 (62%) |
| IV | 131 (6.5%) | 160 (15%) | 170 (8.7%) | 131 (16%) | 592 (10%) |
| Missing | 1811 (47%) | 427 (28%) | 1846 (49%) | 269 (24%) | 4353 (43%) |
| **BMI (kg/m²)** | | | | | |
| Median [Q1, Q3] | 30 [25, 37] | 28 [23, 33] | 29 [25, 34] | 26 [23, 30] | 29 [25, 34] |
| Missing | 181 (4.7%) | 104 (6.9%) | 140 (3.7%) | 78 (7.1%) | 503 (4.9%) |
| **BSA (m²)** | | | | | |
| Median [Q1, Q3] | 1.9 [1.7, 2] | 1.7 [1.6, 1.9] | 2.1 [1.9, 2.3] | 2 [1.8, 2.1] | 1.9 [1.8, 2.1] |
| Missing | 46 (1.2%) | 53 (3.5%) | 42 (1.1%) | 38 (3.5%) | 179 (1.8%) |
| **Height (cm)** | | | | | |
| Median [Q1, Q3] | 160 [160, 170] | 160 [160, 170] | 180 [170, 180] | 170 [170, 180] | 170 [160, 180] |
| Missing | 184 (4.8%) | 95 (6.3%) | 141 (3.7%) | 67 (6.1%) | 487 (4.8%) |
| **Weight (kg)** | | | | | |
| Median [Q1, Q3] | 80 [66, 97] | 71 [59, 86] | 91 [79, 110] | 80 [69, 94] | 83 [69, 100] |
| Missing | 208 (5.4%) | 117 (7.7%) | 147 (3.9%) | 79 (7.2%) | 551 (5.4%) |
| **6MWD (m/6min)** | | | | | |
| Median [Q1, Q3] | 290 [200, 370] | 260 [170, 360] | 310 [230, 410] | 290 [190, 400] | 290 [200, 380] |
| Missing | 2920 (76%) | 903 (60%) | 3140 (83%) | 639 (58%) | 7602 (74%) |
| **BNP (pg/mL)** | | | | | |
| Median [Q1, Q3] | 230 [82, 630] | 300 [120, 690] | 360 [130, 980] | 360 [150, 840] | 300 [110, 810] |
| Missing | 1880 (49%) | 763 (50%) | 1705 (45%) | 504 (46%) | 4852 (47%) |
| **mPAP (mmHg)** | | | | | |
| Median [Q1, Q3] | 30 [26, 35] | 45 [38, 52] | 30 [25, 36] | 45 [38, 51] | 33 [27, 41] |
| Missing | 0 (0%) | 0 (0%) | 0 (0%) | 0 (0%) | 0 (0%) |
| **sPAP (mmHg)** | | | | | |
| Median [Q1, Q3] | 45 [38, 55] | 72 [60, 85] | 45 [38, 55] | 72 [61, 84] | 50 [40, 65] |
| Missing | 176 (4.6%) | 112 (7.4%) | 156 (4.1%) | 95 (8.6%) | 539 (5.3%) |
| **CVP (mmHg)** | | | | | |
| Median [Q1, Q3] | 8 [5, 12] | 9 [5, 14] | 8 [5, 12] | 8 [5, 13] | 8 [5, 13] |
| Missing | 2538 (66%) | 521 (34%) | 2831 (75%) | 410 (37%) | 6300 (62%) |
| **PAWP (mmHg)** | | | | | |
| Median [Q1, Q3] | 17 [13, 22] | 13 [8, 20] | 17 [13, 22] | 12 [8, 18] | 16 [12, 22] |
| Missing | 0 (0%) | 0 (0%) | 0 (0%) | 0 (0%) | 0 (0%) |
| **CO (L/min)** | | | | | |
| Median [Q1, Q3] | 5.1 [4.2, 6.4] | 3.5 [2.8, 4.3] | 5.4 [4.5, 6.6] | 3.8 [3.2, 4.7] | 4.8 [3.8, 6.1] |
| Missing | 0 (0%) | 0 (0%) | 0 (0%) | 0 (0%) | 0 (0%) |
| **CI (L/(min·m²))** | | | | | |
| Median [Q1, Q3] | 2.8 [2.3, 3.4] | 2 [1.7, 2.4] | 2.6 [2.2, 3.1] | 2 [1.7, 2.3] | 2.5 [2, 3.1] |
| Missing | 42 (1.1%) | 52 (3.4%) | 34 (0.9%) | 36 (3.3%) | 164 (1.6%) |
| **PVR (WU)** | | | | | |
| Median [Q1, Q3] | 2.6 [1.8, 3.5] | 8 [6.1, 11] | 2.3 [1.5, 3.2] | 7.3 [5.9, 10] | 3 [1.9, 5.1] |
| Missing | 0 (0%) | 0 (0%) | 0 (0%) | 0 (0%) | 0 (0%) |

(B)

| **Sex** | **female** | | **male** | | **Overall** |
| --- | --- | --- | --- | --- | --- |
| **PVR** | **≤5 WU** | **>5 WU** | **≤5 WU** | **>5 WU** |  |
| N | 182 | 372 | 91 | 173 | 818 |
| **Age at diagnosis (years)** | | | | | |
| Median [Q1, Q3] | 63 [53, 72] | 57 [41, 68] | 57 [47, 69] | 60 [48, 70] | 59 [45, 70] |
| Missing | 0 (0%) | 0 (0%) | 0 (0%) | 0 (0%) | 0 (0%) |
| **WHO FC** | | | | | |
| I | 11 (8.9%) | 9 (3.5%) | 4 (8.7%) | 4 (4.3%) | 28 (5.4%) |
| II | 39 (31%) | 61 (24%) | 18 (39%) | 28 (30%) | 146 (28%) |
| III | 71 (57%) | 141 (55%) | 23 (50%) | 51 (55%) | 286 (55%) |
| IV | 3 (2.4%) | 44 (17%) | 1 (2.2%) | 10 (11%) | 58 (11%) |
| Missing | 58 (32%) | 117 (31%) | 45 (49%) | 80 (46%) | 300 (37%) |
| **BMI (kg/m²)** | | | | | |
| Median [Q1, Q3] | 28 [23, 33] | 25 [22, 30] | 27 [23, 31] | 26 [23, 30] | 26 [23, 31] |
| Missing | 11 (6%) | 23 (6.2%) | 9 (9.9%) | 17 (9.8%) | 60 (7.3%) |
| **BSA (m²)** | | | | | |
| Median [Q1, Q3] | 1.8 [1.6, 1.9] | 1.7 [1.6, 1.9] | 2 [1.8, 2.2] | 2 [1.8, 2.1] | 1.8 [1.6, 2] |
| Missing | 1 (0.55%) | 4 (1.1%) | 1 (1.1%) | 4 (2.3%) | 10 (1.2%) |
| **Height (cm)** | | | | | |
| Median [Q1, Q3] | 160 [160, 170] | 160 [160, 170] | 170 [170, 180] | 180 [170, 180] | 160 [160, 170] |
| Missing | 15 (8.2%) | 29 (7.8%) | 10 (11%) | 14 (8.1%) | 68 (8.3%) |
| **Weight (kg)** | | | | | |
| Median [Q1, Q3] | 71 [62, 85] | 66 [56, 80] | 81 [67, 92] | 80 [69, 94] | 71 [61, 86] |
| Missing | 15 (8.2%) | 29 (7.8%) | 11 (12%) | 16 (9.2%) | 71 (8.7%) |
| **6MWD (m/6min)** | | | | | |
| Median [Q1, Q3] | 330 [240, 420] | 330 [220, 430] | 340 [230, 400] | 350 [200, 490] | 330 [220, 430] |
| Missing | 86 (47%) | 194 (52%) | 54 (59%) | 100 (58%) | 434 (53%) |
| **BNP (pg/mL)** | | | | | |
| Median [Q1, Q3] | 88 [43, 160] | 170 [47, 540] | 120 [35, 290] | 230 [100, 470] | 150 [49, 410] |
| Missing | 105 (58%) | 224 (60%) | 55 (60%) | 99 (57%) | 483 (59%) |
| **mPAP (mmHg)** | | | | | |
| Median [Q1, Q3] | 29 [26, 33] | 46 [40, 55] | 34 [28, 39] | 48 [42, 56] | 41 [32, 51] |
| Missing | 0 (0%) | 0 (0%) | 0 (0%) | 0 (0%) | 0 (0%) |
| **sPAP (mmHg)** | | | | | |
| Median [Q1, Q3] | 47 [41, 54] | 77 [64, 90] | 54 [46, 63] | 78 [68, 87] | 69 [53, 83] |
| Missing | 27 (15%) | 31 (8.3%) | 11 (12%) | 14 (8.1%) | 83 (10%) |
| **CVP (mmHg)** | | | | | |
| Median [Q1, Q3] | 5 [4, 8] | 7 [4, 11] | 5.5 [4, 9] | 7 [5, 10] | 7 [4, 10] |
| Missing | 35 (19%) | 60 (16%) | 17 (19%) | 29 (17%) | 141 (17%) |
| **PAWP (mmHg)** | | | | | |
| Median [Q1, Q3] | 10 [8, 13] | 8.8 [6, 11] | 10 [7, 13] | 9 [7, 11] | 9 [7, 12] |
| Missing | 0 (0%) | 0 (0%) | 0 (0%) | 0 (0%) | 0 (0%) |
| **CO (L/min)** | | | | | |
| Median [Q1, Q3] | 5.2 [4.4, 6] | 3.6 [2.9, 4.5] | 6.5 [5.2, 8.2] | 4.5 [3.6, 5.2] | 4.4 [3.4, 5.5] |
| Missing | 0 (0%) | 0 (0%) | 0 (0%) | 0 (0%) | 0 (0%) |
| **CI (L/(min·m²))** | | | | | |
| Median [Q1, Q3] | 3 [2.5, 3.4] | 2.1 [1.7, 2.6] | 3.1 [2.7, 3.9] | 2.2 [1.9, 2.6] | 2.4 [1.9, 3] |
| Missing | 1 (0.55%) | 4 (1.1%) | 1 (1.1%) | 3 (1.7%) | 9 (1.1%) |
| **PVR (WU)** | | | | | |
| Median [Q1, Q3] | 3.6 [2.9, 4.3] | 9.9 [7.1, 15] | 3.8 [2.9, 4.3] | 8.3 [6.4, 11] | 6.9 [4.3, 11] |
| Missing | 0 (0%) | 0 (0%) | 0 (0%) | 0 (0%) | 0 (0%) |

**Table E6: Distribution of PH-targeting drugs in treated (A) PH and (B) PAH patients.**

PH = Pulmonary Hypertension; PAH = pulmonary arterial hypertension; PDE5i = phosphodiesterase-5 inhibitors; ERA = endothelin receptor antagonists; sGC = soluble guanylate cyclase; PGI2 = prostaglandin I2 and its analogues (inhalative or oral or parenteral).

(A)

| **Sex** | **female** | **male** | **Overall** |
| --- | --- | --- | --- |
| N | 11830 | 9293 | 21123 |
| **PDE5i** | | | |
| treated | 5136 (43%) | 3421 (37%) | 8557 (41%) |
| **ERA** | | | |
| treated | 3266 (28%) | 1458 (16%) | 4724 (22%) |
| **sGC stimulators** | | | |
| treated | 577 (4.9%) | 355 (3.8%) | 932 (4.4%) |
| **PGI2** | | | |
| treated | 1865 (16%) | 1315 (14%) | 3180 (15%) |
| **PDE5i & ERA** | | | |
| treated | 2599 (22%) | 1126 (12%) | 3725 (18%) |
| **ERA & PGI2** | | | |
| treated | 979 (8.3%) | 372 (4%) | 1351 (6.4%) |
| **PDE5i & PGI2** | | | |
| treated | 1258 (11%) | 768 (8.3%) | 2026 (9.6%) |
| **PDE5i & ERA & PGI2** | | | |
| treated | 842 (7.1%) | 316 (3.4%) | 1158 (5.5%) |
| **One or more PH drugs** | | | |
| treated | 6499 (55%) | 4402 (47%) | 10901 (52%) |
| **Treatment kind** | | | |
| none | 5331 (45%) | 4891 (53%) | 10222 (48%) |
| mono | 3132 (26%) | 2640 (28%) | 5772 (27%) |
| dual | 2215 (19%) | 1292 (14%) | 3507 (17%) |
| triple or more | 1152 (9.7%) | 470 (5.1%) | 1622 (7.7%) |

(B)

| **Sex** | **female** | **male** | **Overall** |
| --- | --- | --- | --- |
| N | 3769 | 1605 | 5374 |
| **PDE5i** | | | |
| treated | 2749 (73%) | 1153 (72%) | 3902 (73%) |
| **ERA** | | | |
| treated | 2239 (59%) | 836 (52%) | 3075 (57%) |
| **sGC stimulators** | | | |
| treated | 169 (4.5%) | 69 (4.3%) | 238 (4.4%) |
| **PGI2** | | | |
| treated | 991 (26%) | 356 (22%) | 1347 (25%) |
| **PDE5i & ERA** | | | |
| treated | 1870 (50%) | 683 (43%) | 2553 (48%) |
| **ERA & PGI2** | | | |
| treated | 723 (19%) | 252 (16%) | 975 (18%) |
| **PDE5i & PGI2** | | | |
| treated | 817 (22%) | 291 (18%) | 1108 (21%) |
| **PDE5i & ERA & PGI2** | | | |
| treated | 626 (17%) | 217 (14%) | 843 (16%) |
| **One or more PH drugs** | | | |
| treated | 3215 (85%) | 1341 (84%) | 4556 (85%) |
| **Treatment kind** | | | |
| none | 554 (15%) | 264 (16%) | 818 (15%) |
| mono | 981 (26%) | 516 (32%) | 1497 (28%) |
| dual | 1392 (37%) | 522 (33%) | 1914 (36%) |
| triple or more | 842 (22%) | 303 (19%) | 1145 (21%) |

**Table E7: Baseline characteristics stratified by age and sex in (A) PH and (B) PAH.**

(A)

| **Sex** | **female** | | | **male** | | | **Overall** |
| --- | --- | --- | --- | --- | --- | --- | --- |
| **AgeGroup** | **18-49** | **50-64** | **65-100** | **18-49** | **50-64** | **65-100** |  |
| N | 2643 | 3642 | 5545 | 1624 | 3143 | 4526 | 21123 |
| **Age at diagnosis (years)** | | | | | | | |
| Median [Q1, Q3] | 40 [33, 46] | 58 [54, 61] | 73 [69, 78] | 42 [35, 46] | 58 [54, 62] | 73 [69, 78] | 64 [52, 72] |
| Missing | 0 (0%) | 0 (0%) | 0 (0%) | 0 (0%) | 0 (0%) | 0 (0%) | 0 (0%) |
| **WHO FC** | | | | | | | |
| I | 61 (3.4%) | 81 (3.4%) | 56 (1.4%) | 72 (6.7%) | 92 (4.5%) | 86 (2.7%) | 448 (3.1%) |
| II | 394 (22%) | 450 (19%) | 638 (16%) | 255 (24%) | 455 (22%) | 558 (18%) | 2750 (19%) |
| III | 1121 (62%) | 1533 (64%) | 2860 (71%) | 641 (60%) | 1252 (61%) | 2063 (65%) | 9470 (65%) |
| IV | 221 (12%) | 320 (13%) | 450 (11%) | 108 (10%) | 253 (12%) | 468 (15%) | 1820 (13%) |
| Missing | 846 (32%) | 1258 (35%) | 1541 (28%) | 548 (34%) | 1091 (35%) | 1351 (30%) | 6635 (31%) |
| **BMI (kg/m²)** | | | | | | | |
| Median [Q1, Q3] | 28 [23, 35] | 29 [24, 36] | 28 [24, 33] | 28 [24, 34] | 29 [25, 34] | 27 [24, 31] | 28 [24, 33] |
| Missing | 245 (9.3%) | 295 (8.1%) | 277 (5%) | 105 (6.5%) | 128 (4.1%) | 209 (4.6%) | 1259 (6%) |
| **BSA (m²)** | | | | | | | |
| Median [Q1, Q3] | 1.8 [1.6, 2] | 1.8 [1.7, 2] | 1.8 [1.6, 1.9] | 2.1 [1.9, 2.3] | 2.1 [1.9, 2.3] | 2 [1.9, 2.1] | 1.9 [1.7, 2.1] |
| Missing | 90 (3.4%) | 82 (2.3%) | 104 (1.9%) | 28 (1.7%) | 41 (1.3%) | 74 (1.6%) | 419 (2%) |
| **Height (cm)** | | | | | | | |
| Median [Q1, Q3] | 160 [160, 170] | 160 [160, 170] | 160 [160, 170] | 180 [170, 180] | 180 [170, 180] | 180 [170, 180] | 170 [160, 180] |
| Missing | 267 (10%) | 305 (8.4%) | 327 (5.9%) | 113 (7%) | 144 (4.6%) | 235 (5.2%) | 1391 (6.6%) |
| **Weight (kg)** | | | | | | | |
| Median [Q1, Q3] | 75 [61, 94] | 77 [64, 94] | 72 [61, 85] | 88 [75, 110] | 90 [77, 110] | 84 [73, 97] | 80 [67, 96] |
| Missing | 287 (11%) | 335 (9.2%) | 369 (6.7%) | 121 (7.5%) | 143 (4.5%) | 261 (5.8%) | 1516 (7.2%) |
| **6MWD (m/6min)** | | | | | | | |
| Median [Q1, Q3] | 340 [250, 430] | 290 [190, 380] | 250 [180, 330] | 390 [280, 470] | 330 [220, 420] | 260 [190, 350] | 290 [200, 380] |
| Missing | 1400 (53%) | 2071 (57%) | 3066 (55%) | 1061 (65%) | 2086 (66%) | 2761 (61%) | 12445 (59%) |
| **BNP (pg/mL)** | | | | | | | |
| Median [Q1, Q3] | 200 [60, 520] | 200 [64, 560] | 240 [100, 570] | 280 [86, 820] | 300 [100, 780] | 310 [130, 740] | 260 [93, 650] |
| Missing | 1155 (44%) | 1637 (45%) | 2486 (45%) | 688 (42%) | 1285 (41%) | 1874 (41%) | 9125 (43%) |
| **mPAP (mmHg)** | | | | | | | |
| Median [Q1, Q3] | 44 [32, 55] | 39 [30, 50] | 37 [29, 46] | 40 [29, 51] | 37 [28, 46] | 36 [29, 44] | 38 [29, 48] |
| Missing | 0 (0%) | 0 (0%) | 0 (0%) | 0 (0%) | 0 (0%) | 0 (0%) | 0 (0%) |
| **sPAP (mmHg)** | | | | | | | |
| Median [Q1, Q3] | 68 [47, 86] | 61 [45, 81] | 60 [46, 77] | 59 [42, 79] | 56 [42, 73] | 57 [44, 73] | 60 [45, 78] |
| Missing | 133 (5%) | 221 (6.1%) | 428 (7.7%) | 87 (5.4%) | 176 (5.6%) | 341 (7.5%) | 1386 (6.6%) |
| **CVP (mmHg)** | | | | | | | |
| Median [Q1, Q3] | 8 [5, 13] | 8 [5, 12] | 8 [5, 12] | 8 [5, 12] | 8 [4, 12] | 8 [5, 12] | 8 [5, 12] |
| Missing | 1354 (51%) | 1952 (54%) | 2820 (51%) | 1027 (63%) | 1993 (63%) | 2514 (56%) | 11660 (55%) |
| **PAWP (mmHg)** | | | | | | | |
| Median [Q1, Q3] | 11 [8, 16] | 12 [9, 18] | 14 [10, 19] | 13 [9, 20] | 14 [10, 20] | 14 [10, 19] | 13 [9, 19] |
| Missing | 0 (0%) | 0 (0%) | 0 (0%) | 0 (0%) | 0 (0%) | 0 (0%) | 0 (0%) |
| **CO (L/min)** | | | | | | | |
| Median [Q1, Q3] | 4.2 [3.2, 5.5] | 4.5 [3.5, 5.7] | 4.2 [3.4, 5.3] | 5 [3.9, 6.2] | 5 [3.9, 6.2] | 4.7 [3.8, 5.8] | 4.5 [3.6, 5.7] |
| Missing | 0 (0%) | 0 (0%) | 0 (0%) | 0 (0%) | 0 (0%) | 0 (0%) | 0 (0%) |
| **CI (L/(min·m²))** | | | | | | | |
| Median [Q1, Q3] | 2.3 [1.8, 3] | 2.5 [2, 3.1] | 2.4 [2, 2.9] | 2.4 [1.9, 3] | 2.4 [1.9, 2.9] | 2.3 [1.9, 2.8] | 2.4 [1.9, 2.9] |
| Missing | 87 (3.3%) | 81 (2.2%) | 101 (1.8%) | 22 (1.4%) | 36 (1.1%) | 73 (1.6%) | 400 (1.9%) |
| **PVR (WU)** | | | | | | | |
| Median [Q1, Q3] | 7 [3.3, 13] | 5.2 [2.9, 9.5] | 4.9 [2.9, 8.7] | 4.2 [2.1, 8.5] | 3.7 [2.1, 7.2] | 4.2 [2.4, 7.1] | 4.7 [2.6, 8.7] |
| Missing | 0 (0%) | 0 (0%) | 0 (0%) | 0 (0%) | 0 (0%) | 0 (0%) | 0 (0%) |

(B)

| **Sex** | **female** | | | **male** | | | **Overall** |
| --- | --- | --- | --- | --- | --- | --- | --- |
| **AgeGroup** | **18-49** | **50-64** | **65-100** | **18-49** | **50-64** | **65-100** |  |
| N | 1210 | 1169 | 1390 | 406 | 527 | 672 | 5374 |
| **Age at diagnosis (years)** | | | | | | | |
| Median [Q1, Q3] | 38 [31, 44] | 58 [54, 61] | 72 [69, 77] | 41 [34, 46] | 58 [54, 61] | 73 [69, 77] | 60 [47, 70] |
| Missing | 0 (0%) | 0 (0%) | 0 (0%) | 0 (0%) | 0 (0%) | 0 (0%) | 0 (0%) |
| **WHO FC** | | | | | | | |
| I | 32 (3.4%) | 27 (3%) | 11 (0.96%) | 11 (3.5%) | 9 (2.2%) | 6 (1.1%) | 96 (2.3%) |
| II | 192 (21%) | 179 (20%) | 183 (16%) | 83 (27%) | 84 (21%) | 71 (13%) | 792 (19%) |
| III | 581 (62%) | 587 (65%) | 811 (71%) | 193 (62%) | 268 (66%) | 385 (69%) | 2825 (66%) |
| IV | 128 (14%) | 109 (12%) | 145 (13%) | 26 (8.3%) | 45 (11%) | 97 (17%) | 550 (13%) |
| Missing | 277 (23%) | 267 (23%) | 240 (17%) | 93 (23%) | 121 (23%) | 113 (17%) | 1111 (21%) |
| **BMI (kg/m²)** | | | | | | | |
| Median [Q1, Q3] | 26 [22, 31] | 28 [24, 33] | 26 [23, 31] | 26 [23, 31] | 28 [24, 32] | 27 [24, 30] | 27 [23, 31] |
| Missing | 170 (14%) | 126 (11%) | 76 (5.5%) | 38 (9.4%) | 33 (6.3%) | 38 (5.7%) | 481 (9%) |
| **BSA (m²)** | | | | | | | |
| Median [Q1, Q3] | 1.8 [1.6, 1.9] | 1.8 [1.6, 1.9] | 1.7 [1.6, 1.8] | 2 [1.9, 2.2] | 2 [1.9, 2.2] | 1.9 [1.8, 2.1] | 1.8 [1.6, 2] |
| Missing | 71 (5.9%) | 37 (3.2%) | 37 (2.7%) | 8 (2%) | 14 (2.7%) | 13 (1.9%) | 180 (3.3%) |
| **Height (cm)** | | | | | | | |
| Median [Q1, Q3] | 160 [160, 170] | 160 [160, 170] | 160 [150, 160] | 180 [170, 180] | 180 [170, 180] | 170 [170, 180] | 160 [160, 170] |
| Missing | 186 (15%) | 144 (12%) | 115 (8.3%) | 46 (11%) | 46 (8.7%) | 46 (6.8%) | 583 (11%) |
| **Weight (kg)** | | | | | | | |
| Median [Q1, Q3] | 68 [58, 84] | 73 [60, 87] | 67 [58, 78] | 81 [70, 96] | 86 [74, 99] | 80 [71, 91] | 74 [62, 87] |
| Missing | 200 (17%) | 152 (13%) | 122 (8.8%) | 47 (12%) | 46 (8.7%) | 53 (7.9%) | 620 (12%) |
| **6MWD (m/6min)** | | | | | | | |
| Median [Q1, Q3] | 350 [260, 430] | 300 [200, 400] | 240 [180, 330] | 400 [300, 480] | 320 [220, 420] | 250 [170, 340] | 300 [200, 400] |
| Missing | 410 (34%) | 438 (37%) | 564 (41%) | 138 (34%) | 190 (36%) | 259 (39%) | 1999 (37%) |
| **BNP (pg/mL)** | | | | | | | |
| Median [Q1, Q3] | 170 [50, 410] | 160 [60, 400] | 220 [87, 560] | 130 [39, 270] | 180 [70, 390] | 240 [100, 530] | 180 [69, 440] |
| Missing | 535 (44%) | 519 (44%) | 669 (48%) | 201 (50%) | 229 (43%) | 291 (43%) | 2444 (45%) |
| **mPAP (mmHg)** | | | | | | | |
| Median [Q1, Q3] | 52 [42, 61] | 45 [36, 55] | 41 [32, 50] | 50 [42, 61] | 47 [39, 55] | 42 [36, 50] | 46 [36, 55] |
| Missing | 0 (0%) | 0 (0%) | 0 (0%) | 0 (0%) | 0 (0%) | 0 (0%) | 0 (0%) |
| **sPAP (mmHg)** | | | | | | | |
| Median [Q1, Q3] | 81 [66, 95] | 75 [57, 90] | 69 [52, 84] | 80 [66, 94] | 76 [62, 88] | 71 [59, 83] | 75 [59, 89] |
| Missing | 73 (6%) | 80 (6.8%) | 128 (9.2%) | 35 (8.6%) | 38 (7.2%) | 70 (10%) | 424 (7.9%) |
| **CVP (mmHg)** | | | | | | | |
| Median [Q1, Q3] | 8 [5, 12] | 8 [5, 11] | 7 [4, 10] | 7 [4, 11] | 8 [5, 11] | 7 [5, 10] | 7 [5, 11] |
| Missing | 446 (37%) | 475 (41%) | 637 (46%) | 141 (35%) | 189 (36%) | 273 (41%) | 2161 (40%) |
| **PAWP (mmHg)** | | | | | | | |
| Median [Q1, Q3] | 8 [6, 11] | 10 [7, 12] | 10 [7.2, 12] | 9 [7, 12] | 10 [7, 12] | 10 [7, 12] | 9 [7, 12] |
| Missing | 0 (0%) | 0 (0%) | 0 (0%) | 0 (0%) | 0 (0%) | 0 (0%) | 0 (0%) |
| **CO (L/min)** | | | | | | | |
| Median [Q1, Q3] | 3.8 [3, 4.9] | 4.2 [3.3, 5.2] | 4 [3.1, 5] | 4.7 [3.8, 5.9] | 4.6 [3.6, 5.7] | 4.4 [3.5, 5.3] | 4.2 [3.2, 5.2] |
| Missing | 0 (0%) | 0 (0%) | 0 (0%) | 0 (0%) | 0 (0%) | 0 (0%) | 0 (0%) |
| **CI (L/(min·m²))** | | | | | | | |
| Median [Q1, Q3] | 2.2 [1.7, 2.8] | 2.3 [1.9, 2.9] | 2.3 [1.9, 2.9] | 2.3 [1.9, 3] | 2.3 [1.8, 2.7] | 2.2 [1.8, 2.7] | 2.3 [1.8, 2.8] |
| Missing | 71 (5.9%) | 37 (3.2%) | 36 (2.6%) | 7 (1.7%) | 13 (2.5%) | 13 (1.9%) | 177 (3.3%) |
| **PVR (WU)** | | | | | | | |
| Median [Q1, Q3] | 11 [7.3, 17] | 8.6 [5.4, 13] | 7.7 [4.6, 12] | 8.9 [5.8, 13] | 8.1 [5.5, 12] | 7.3 [5.1, 10] | 8.7 [5.5, 13] |
| Missing | 0 (0%) | 0 (0%) | 0 (0%) | 0 (0%) | 0 (0%) | 0 (0%) | 0 (0%) |

**Table E8: Distribution of comorbidities for the (A) PH patient group and the (B) PAH patient group.**

PH = pulmonary hypertension; PAH = pulmonary artery hypertension; eGFR = estimated glomerular filtration rate.

(A)

| **Sex** | **female** | **male** | **Overall** |
| --- | --- | --- | --- |
| N | 11830 | 9293 | 21123 |
| **Cardiovascular Disease** | | | |
| TRUE | 5242 (44%) | 5321 (57%) | 10563 (50%) |
| Missing | 2400 (20%) | 1384 (15%) | 3784 (18%) |
| **Obesity** | | | |
| TRUE | 4645 (42%) | 3304 (37%) | 7949 (40%) |
| Missing | 817 (6.9%) | 442 (4.8%) | 1259 (6%) |
| **Renal Failure: eGFR <90 mL/min/1.73m²** | | | |
| ≥90 | 6064 (51%) | 5238 (56%) | 11302 (54%) |
| Missing | 3916 (33%) | 2376 (26%) | 6292 (30%) |

(B)

| **Sex** | **female** | **male** | **Overall** |
| --- | --- | --- | --- |
| N | 3769 | 1605 | 5374 |
| **Cardiovascular Disease** | | | |
| TRUE | 1209 (32%) | 655 (41%) | 1864 (35%) |
| Missing | 1094 (29%) | 410 (26%) | 1504 (28%) |
| **Obesity** | | | |
| TRUE | 1110 (42%) | 454 (37%) | 1564 (29%) |
| Missing | 372 (10 %) | 109 (7%) | 481 (9%) |
| **Renal Failure: eGFR <90 mL/min/1.73m²** | | | |
| ≥90 | 1517 (40%) | 583 (36%) | 2100 (39%) |
| Missing | 1651 (44%) | 741 (46%) | 2392 (45%) |

**Table E9: Baseline characteristics stratified by sex for (A) PH and (B) PAH patients with cardiovascular comorbidities.**

Baseline characteristics are shown for patients from centers with reported cardiovascular comorbidities.

(A)

| **Sex** | **female** | **male** | **Overall** |
| --- | --- | --- | --- |
| N | 5242 | 5321 | 10563 |
| **Age at diagnosis (years)** | | | |
| Median [Q1, Q3] | 67 [57, 74] | 66 [57, 74] | 66 [57, 74] |
| Missing | 0 (0%) | 0 (0%) | 0 (0%) |
| **WHO FC** | | | |
| I | 90 (2.9%) | 156 (4.9%) | 246 (3.9%) |
| II | 679 (22%) | 728 (23%) | 1407 (22%) |
| III | 2020 (65%) | 1886 (59%) | 3906 (62%) |
| IV | 328 (11%) | 401 (13%) | 729 (12%) |
| Missing | 2125 (41%) | 2150 (40%) | 4275 (40%) |
| **BMI (kg/m²)** | | | |
| Median [Q1, Q3] | 29 [25, 35] | 29 [25, 33] | 29 [25, 34] |
| Missing | 390 (7.4%) | 242 (4.5%) | 632 (6%) |
| **BSA (m²)** | | | |
| Median [Q1, Q3] | 1.8 [1.7, 2] | 2.1 [1.9, 2.2] | 1.9 [1.8, 2.1] |
| Missing | 87 (1.7%) | 56 (1.1%) | 143 (1.4%) |
| **Height (cm)** | | | |
| Median [Q1, Q3] | 160 [160, 170] | 180 [170, 180] | 170 [160, 180] |
| Missing | 501 (9.6%) | 312 (5.9%) | 813 (7.7%) |
| **Weight (kg)** | | | |
| Median [Q1, Q3] | 76 [64, 92] | 89 [77, 100] | 83 [70, 99] |
| Missing | 515 (9.8%) | 311 (5.8%) | 826 (7.8%) |
| **6MWD (m/6min)** | | | |
| Median [Q1, Q3] | 280 [180, 350] | 300 [200, 390] | 290 [190, 370] |
| Missing | 3277 (63%) | 3905 (73%) | 7182 (68%) |
| **BNP (pg/mL)** | | | |
| Median [Q1, Q3] | 260 [99, 630] | 360 [140, 860] | 300 [110, 760] |
| Missing | 1763 (34%) | 1756 (33%) | 3519 (33%) |
| **mPAP (mmHg)** | | | |
| Median [Q1, Q3] | 35 [28, 45] | 34 [27, 43] | 35 [28, 44] |
| Missing | 0 (0%) | 0 (0%) | 0 (0%) |
| **sPAP (mmHg)** | | | |
| Median [Q1, Q3] | 55 [43, 74] | 52 [40, 68] | 54 [42, 70] |
| Missing | 322 (6.1%) | 267 (5%) | 589 (5.6%) |
| **CVP (mmHg)** | | | |
| Median [Q1, Q3] | 8 [5, 12] | 8 [5, 12] | 8 [5, 12] |
| Missing | 2591 (49%) | 3355 (63%) | 5946 (56%) |
| **PAWP (mmHg)** | | | |
| Median [Q1, Q3] | 14 [10, 20] | 15 [10, 22] | 15 [10, 21] |
| Missing | 0 (0%) | 0 (0%) | 0 (0%) |
| **CO (L/min)** | | | |
| Median [Q1, Q3] | 4.5 [3.5, 5.6] | 4.9 [4, 6] | 4.7 [3.8, 5.8] |
| Missing | 0 (0%) | 0 (0%) | 0 (0%) |
| **CI (L/(min·m²))** | | | |
| Median [Q1, Q3] | 2.5 [2, 3] | 2.4 [2, 2.9] | 2.4 [2, 3] |
| Missing | 81 (1.5%) | 45 (0.85%) | 126 (1.2%) |
| **PVR (WU)** | | | |
| Median [Q1, Q3] | 4.1 [2.5, 7.4] | 3.1 [1.9, 5.6] | 3.6 [2.1, 6.4] |
| Missing | 0 (0%) | 0 (0%) | 0 (0%) |

(B)

| **Sex** | **female** | **male** | **Overall** |
| --- | --- | --- | --- |
| N | 1209 | 655 | 1864 |
| **Age at diagnosis (years)** | | | |
| Median [Q1, Q3] | 64 [55, 73] | 66 [56, 74] | 65 [55, 73] |
| Missing | 0 (0%) | 0 (0%) | 0 (0%) |
| **WHO FC** | | | |
| I | 18 (2.1%) | 12 (2.5%) | 30 (2.2%) |
| II | 200 (23%) | 102 (21%) | 302 (22%) |
| III | 548 (64%) | 314 (64%) | 862 (64%) |
| IV | 89 (10%) | 60 (12%) | 149 (11%) |
| Missing | 354 (29%) | 167 (25%) | 521 (28%) |
| **BMI (kg/m²)** | | | |
| Median [Q1, Q3] | 28 [24, 32] | 27 [24, 31] | 27 [24, 32] |
| Missing | 145 (12%) | 58 (8.9%) | 203 (11%) |
| **BSA (m²)** | | | |
| Median [Q1, Q3] | 1.8 [1.6, 1.9] | 2 [1.9, 2.1] | 1.8 [1.7, 2] |
| Missing | 60 (5%) | 18 (2.7%) | 78 (4.2%) |
| **Height (cm)** | | | |
| Median [Q1, Q3] | 160 [160, 170] | 170 [170, 180] | 160 [160, 170] |
| Missing | 204 (17%) | 79 (12%) | 283 (15%) |
| **Weight (kg)** | | | |
| Median [Q1, Q3] | 71 [60, 85] | 83 [72, 94] | 75 [64, 89] |
| Missing | 211 (17%) | 84 (13%) | 295 (16%) |
| **6MWD (m/6min)** | | | |
| Median [Q1, Q3] | 280 [180, 360] | 310 [200, 400] | 290 [190, 380] |
| Missing | 465 (38%) | 234 (36%) | 699 (38%) |
| **BNP (pg/mL)** | | | |
| Median [Q1, Q3] | 190 [76, 480] | 210 [86, 480] | 200 [82, 480] |
| Missing | 369 (31%) | 189 (29%) | 558 (30%) |
| **mPAP (mmHg)** | | | |
| Median [Q1, Q3] | 42 [32, 52] | 44 [36, 51] | 43 [34, 52] |
| Missing | 0 (0%) | 0 (0%) | 0 (0%) |
| **sPAP (mmHg)** | | | |
| Median [Q1, Q3] | 70 [54, 86] | 73 [59, 84] | 72 [55, 85] |
| Missing | 89 (7.4%) | 55 (8.4%) | 144 (7.7%) |
| **CVP (mmHg)** | | | |
| Median [Q1, Q3] | 7 [4, 11] | 7 [5, 10] | 7 [4, 10] |
| Missing | 320 (26%) | 153 (23%) | 473 (25%) |
| **PAWP (mmHg)** | | | |
| Median [Q1, Q3] | 10 [7, 12] | 10 [7, 12] | 10 [7, 12] |
| Missing | 0 (0%) | 0 (0%) | 0 (0%) |
| **CO (L/min)** | | | |
| Median [Q1, Q3] | 4.2 [3.3, 5.1] | 4.6 [3.6, 5.5] | 4.3 [3.4, 5.3] |
| Missing | 0 (0%) | 0 (0%) | 0 (0%) |
| **CI (L/(min·m²))** | | | |
| Median [Q1, Q3] | 2.3 [1.9, 2.9] | 2.2 [1.9, 2.7] | 2.3 [1.9, 2.8] |
| Missing | 59 (4.9%) | 17 (2.6%) | 76 (4.1%) |
| **PVR (WU)** | | | |
| Median [Q1, Q3] | 7.8 [4.7, 12] | 7.3 [5, 10] | 7.6 [4.8, 11] |
| Missing | 0 (0%) | 0 (0%) | 0 (0%) |

**Table E10: Baseline characteristics stratified by sex for obese (A) PH and (B) PAH patients.**

Baseline characteristics are shown for patients from centers with reported obesity.

(A)

| **Sex** | **female** | **male** | **Overall** |
| --- | --- | --- | --- |
| N | 4907 | 3445 | 8352 |
| **Age at diagnosis (years)** | | | |
| Median [Q1, Q3] | 62 [51, 71] | 62 [53, 70] | 62 [52, 70] |
| Missing | 0 (0%) | 0 (0%) | 0 (0%) |
| **WHO FC** | | | |
| I | 74 (2.3%) | 74 (3.4%) | 148 (2.7%) |
| II | 511 (16%) | 410 (19%) | 921 (17%) |
| III | 2265 (70%) | 1453 (66%) | 3718 (68%) |
| IV | 389 (12%) | 253 (12%) | 642 (12%) |
| Missing | 1668 (34%) | 1255 (36%) | 2923 (35%) |
| **BMI (kg/m²)** | | | |
| Median [Q1, Q3] | 36 [32, 41] | 34 [32, 38] | 35 [32, 39] |
| Missing | 262 (5.3%) | 141 (4.1%) | 403 (4.8%) |
| **BSA (m²)** | | | |
| Median [Q1, Q3] | 2 [1.8, 2.1] | 2.2 [2.1, 2.4] | 2.1 [1.9, 2.3] |
| Missing | 102 (2.1%) | 58 (1.7%) | 160 (1.9%) |
| **Height (cm)** | | | |
| Median [Q1, Q3] | 160 [160, 170] | 180 [170, 180] | 170 [160, 180] |
| Missing | 307 (6.3%) | 178 (5.2%) | 485 (5.8%) |
| **Weight (kg)** | | | |
| Median [Q1, Q3] | 93 [84, 110] | 110 [97, 120] | 100 [88, 110] |
| Missing | 347 (7.1%) | 187 (5.4%) | 534 (6.4%) |
| **6MWD (m/6min)** | | | |
| Median [Q1, Q3] | 240 [170, 340] | 280 [200, 370] | 260 [180, 350] |
| Missing | 2981 (61%) | 2400 (70%) | 5381 (64%) |
| **BNP (pg/mL)** | | | |
| Median [Q1, Q3] | 190 [72, 470] | 250 [92, 580] | 220 [79, 520] |
| Missing | 2293 (47%) | 1507 (44%) | 3800 (45%) |
| **mPAP (mmHg)** | | | |
| Median [Q1, Q3] | 39 [30, 50] | 36 [28, 46] | 38 [29, 48] |
| Missing | 0 (0%) | 0 (0%) | 0 (0%) |
| **sPAP (mmHg)** | | | |
| Median [Q1, Q3] | 60 [45, 80] | 54 [41, 72] | 58 [44, 77] |
| Missing | 229 (4.7%) | 166 (4.8%) | 395 (4.7%) |
| **CVP (mmHg)** | | | |
| Median [Q1, Q3] | 10 [6, 14] | 10 [6, 13] | 10 [6, 14] |
| Missing | 2758 (56%) | 2326 (68%) | 5084 (61%) |
| **PAWP (mmHg)** | | | |
| Median [Q1, Q3] | 15 [10, 20] | 15 [11, 21] | 15 [11, 20] |
| Missing | 0 (0%) | 0 (0%) | 0 (0%) |
| **CO (L/min)** | | | |
| Median [Q1, Q3] | 4.8 [3.8, 6] | 5.4 [4.4, 6.6] | 5 [4, 6.3] |
| Missing | 0 (0%) | 0 (0%) | 0 (0%) |
| **CI (L/(min·m²))** | | | |
| Median [Q1, Q3] | 2.4 [2, 3] | 2.4 [2, 2.9] | 2.4 [2, 3] |
| Missing | 100 (2%) | 48 (1.4%) | 148 (1.8%) |
| **PVR (WU)** | | | |
| Median [Q1, Q3] | 4.3 [2.5, 8] | 3.1 [1.8, 5.7] | 3.8 [2.2, 6.9] |
| Missing | 0 (0%) | 0 (0%) | 0 (0%) |

(B)

| **Sex** | **female** | **male** | **Overall** |
| --- | --- | --- | --- |
| N | 1203 | 483 | 1686 |
| **Age at diagnosis (years)** | | | |
| Median [Q1, Q3] | 58 [46, 68] | 59 [51, 69] | 59 [48, 68] |
| Missing | 0 (0%) | 0 (0%) | 0 (0%) |
| **WHO FC** | | | |
| I | 13 (1.4%) | 3 (0.79%) | 16 (1.2%) |
| II | 141 (16%) | 63 (16%) | 204 (16%) |
| III | 624 (69%) | 266 (70%) | 890 (69%) |
| IV | 129 (14%) | 50 (13%) | 179 (14%) |
| Missing | 296 (25%) | 101 (21%) | 397 (24%) |
| **BMI (kg/m²)** | | | |
| Median [Q1, Q3] | 35 [32, 38] | 34 [31, 36] | 34 [32, 38] |
| Missing | 93 (7.7%) | 29 (6%) | 122 (7.2%) |
| **BSA (m²)** | | | |
| Median [Q1, Q3] | 1.9 [1.8, 2.1] | 2.2 [2.1, 2.3] | 2 [1.9, 2.2] |
| Missing | 49 (4.1%) | 15 (3.1%) | 64 (3.8%) |
| **Height (cm)** | | | |
| Median [Q1, Q3] | 160 [160, 170] | 170 [170, 180] | 160 [160, 170] |
| Missing | 122 (10%) | 45 (9.3%) | 167 (9.9%) |
| **Weight (kg)** | | | |
| Median [Q1, Q3] | 89 [82, 100] | 100 [94, 110] | 93 [84, 110] |
| Missing | 136 (11%) | 47 (9.7%) | 183 (11%) |
| **6MWD (m/6min)** | | | |
| Median [Q1, Q3] | 260 [180, 360] | 280 [200, 390] | 270 [180, 370] |
| Missing | 489 (41%) | 169 (35%) | 658 (39%) |
| **BNP (pg/mL)** | | | |
| Median [Q1, Q3] | 160 [62, 390] | 190 [75, 380] | 170 [66, 380] |
| Missing | 580 (48%) | 221 (46%) | 801 (48%) |
| **mPAP (mmHg)** | | | |
| Median [Q1, Q3] | 48 [38, 56] | 48 [40, 55] | 48 [38, 56] |
| Missing | 0 (0%) | 0 (0%) | 0 (0%) |
| **sPAP (mmHg)** | | | |
| Median [Q1, Q3] | 76 [60, 92] | 77 [65, 90] | 77 [62, 90] |
| Missing | 68 (5.7%) | 35 (7.2%) | 103 (6.1%) |
| **CVP (mmHg)** | | | |
| Median [Q1, Q3] | 8 [6, 12] | 9 [6, 12] | 8 [6, 12] |
| Missing | 497 (41%) | 196 (41%) | 693 (41%) |
| **PAWP (mmHg)** | | | |
| Median [Q1, Q3] | 10 [8, 12] | 11 [8, 13] | 10 [8, 13] |
| Missing | 0 (0%) | 0 (0%) | 0 (0%) |
| **CO (L/min)** | | | |
| Median [Q1, Q3] | 4.4 [3.5, 5.4] | 5 [4, 6] | 4.5 [3.6, 5.6] |
| Missing | 0 (0%) | 0 (0%) | 0 (0%) |
| **CI (L/(min·m²))** | | | |
| Median [Q1, Q3] | 2.2 [1.8, 2.8] | 2.3 [1.9, 2.8] | 2.3 [1.8, 2.8] |
| Missing | 49 (4.1%) | 13 (2.7%) | 62 (3.7%) |
| **PVR (WU)** | | | |
| Median [Q1, Q3] | 8.5 [5.1, 12] | 7.2 [5.2, 10] | 8.1 [5.1, 12] |
| Missing | 0 (0%) | 0 (0%) | 0 (0%) |

**Table E11: Baseline characteristics stratified by sex for (A) PH and (B) PAH patients with chronic kidney disease.**

Baseline characteristics are shown for patients from centers with reported eGFR value.

(A)

| **Sex** | **female** | **male** | **Overall** |
| --- | --- | --- | --- |
| N | 6064 | 5238 | 11302 |
| **Age at diagnosis (years)** | | | |
| Median [Q1, Q3] | 65 [55, 74] | 66 [57, 74] | 65 [56, 74] |
| Missing | 0 (0%) | 0 (0%) | 0 (0%) |
| **WHO FC** | | | |
| I | 110 (2.8%) | 152 (4.6%) | 262 (3.6%) |
| II | 736 (19%) | 715 (21%) | 1451 (20%) |
| III | 2512 (64%) | 1961 (59%) | 4473 (62%) |
| IV | 546 (14%) | 498 (15%) | 1044 (14%) |
| Missing | 2160 (36%) | 1912 (37%) | 4072 (36%) |
| **BMI (kg/m²)** | | | |
| Median [Q1, Q3] | 29 [24, 34] | 28 [25, 32] | 28 [24, 33] |
| Missing | 452 (7.5%) | 241 (4.6%) | 693 (6.1%) |
| **BSA (m²)** | | | |
| Median [Q1, Q3] | 1.8 [1.7, 2] | 2 [1.9, 2.2] | 1.9 [1.7, 2.1] |
| Missing | 116 (1.9%) | 70 (1.3%) | 186 (1.6%) |
| **Height (cm)** | | | |
| Median [Q1, Q3] | 160 [160, 170] | 180 [170, 180] | 170 [160, 180] |
| Missing | 535 (8.8%) | 300 (5.7%) | 835 (7.4%) |
| **Weight (kg)** | | | |
| Median [Q1, Q3] | 75 [63, 90] | 88 [76, 100] | 81 [68, 97] |
| Missing | 579 (9.5%) | 311 (5.9%) | 890 (7.9%) |
| **6MWD (m/6min)** | | | |
| Median [Q1, Q3] | 280 [180, 370] | 300 [190, 390] | 290 [180, 380] |
| Missing | 3423 (56%) | 3571 (68%) | 6994 (62%) |
| **BNP (pg/mL)** | | | |
| Median [Q1, Q3] | 270 [100, 670] | 380 [150, 930] | 320 [120, 780] |
| Missing | 1481 (24%) | 1359 (26%) | 2840 (25%) |
| **mPAP (mmHg)** | | | |
| Median [Q1, Q3] | 37 [29, 48] | 35 [27, 44] | 36 [28, 46] |
| Missing | 0 (0%) | 0 (0%) | 0 (0%) |
| **sPAP (mmHg)** | | | |
| Median [Q1, Q3] | 59 [44, 78] | 54 [41, 70] | 56 [43, 75] |
| Missing | 374 (6.2%) | 290 (5.5%) | 664 (5.9%) |
| **CVP (mmHg)** | | | |
| Median [Q1, Q3] | 8 [5, 12] | 8 [5, 12] | 8 [5, 12] |
| Missing | 2843 (47%) | 3097 (59%) | 5940 (53%) |
| **PAWP (mmHg)** | | | |
| Median [Q1, Q3] | 14 [9, 19] | 15 [10, 22] | 14 [10, 20] |
| Missing | 0 (0%) | 0 (0%) | 0 (0%) |
| **CO (L/min)** | | | |
| Median [Q1, Q3] | 4.2 [3.3, 5.4] | 4.8 [3.8, 6] | 4.5 [3.6, 5.6] |
| Missing | 0 (0%) | 0 (0%) | 0 (0%) |
| **CI (L/(min·m²))** | | | |
| Median [Q1, Q3] | 2.3 [1.9, 2.9] | 2.3 [1.9, 2.8] | 2.3 [1.9, 2.9] |
| Missing | 111 (1.8%) | 62 (1.2%) | 173 (1.5%) |
| **PVR (WU)** | | | |
| Median [Q1, Q3] | 4.8 [2.7, 9.4] | 3.3 [1.9, 6.3] | 4 [2.3, 7.8] |
| Missing | 0 (0%) | 0 (0%) | 0 (0%) |

(B)

| **Sex** | **female** | **male** | **Overall** |
| --- | --- | --- | --- |
| N | 1517 | 583 | 2100 |
| **Age at diagnosis (years)** | | | |
| Median [Q1, Q3] | 60 [48, 70] | 62 [51, 72] | 60 [49, 70] |
| Missing | 0 (0%) | 0 (0%) | 0 (0%) |
| **WHO FC** | | | |
| I | 25 (2.1%) | 11 (2.4%) | 36 (2.2%) |
| II | 222 (19%) | 92 (20%) | 314 (19%) |
| III | 767 (64%) | 290 (63%) | 1057 (64%) |
| IV | 183 (15%) | 70 (15%) | 253 (15%) |
| Missing | 320 (21%) | 120 (21%) | 440 (21%) |
| **BMI (kg/m²)** | | | |
| Median [Q1, Q3] | 27 [23, 32] | 27 [24, 31] | 27 [23, 31] |
| Missing | 182 (12%) | 40 (6.9%) | 222 (11%) |
| **BSA (m²)** | | | |
| Median [Q1, Q3] | 1.8 [1.6, 1.9] | 2 [1.9, 2.2] | 1.8 [1.7, 2] |
| Missing | 39 (2.6%) | 12 (2.1%) | 51 (2.4%) |
| **Height (cm)** | | | |
| Median [Q1, Q3] | 160 [160, 170] | 180 [170, 180] | 160 [160, 170] |
| Missing | 244 (16%) | 70 (12%) | 314 (15%) |
| **Weight (kg)** | | | |
| Median [Q1, Q3] | 69 [59, 83] | 83 [72, 94] | 74 [62, 88] |
| Missing | 245 (16%) | 70 (12%) | 315 (15%) |
| **6MWD (m/6min)** | | | |
| Median [Q1, Q3] | 300 [190, 380] | 300 [200, 410] | 300 [190, 390] |
| Missing | 449 (30%) | 154 (26%) | 603 (29%) |
| **BNP (pg/mL)** | | | |
| Median [Q1, Q3] | 230 [89, 540] | 250 [99, 520] | 240 [92, 540] |
| Missing | 291 (19%) | 124 (21%) | 415 (20%) |
| **mPAP (mmHg)** | | | |
| Median [Q1, Q3] | 47 [36, 56] | 47 [40, 54] | 47 [38, 56] |
| Missing | 0 (0%) | 0 (0%) | 0 (0%) |
| **sPAP (mmHg)** | | | |
| Median [Q1, Q3] | 76 [61, 92] | 76 [65, 87] | 76 [62, 90] |
| Missing | 109 (7.2%) | 40 (6.9%) | 149 (7.1%) |
| **CVP (mmHg)** | | | |
| Median [Q1, Q3] | 7 [4.8, 11] | 8 [5, 12] | 8 [5, 11] |
| Missing | 365 (24%) | 90 (15%) | 455 (22%) |
| **PAWP (mmHg)** | | | |
| Median [Q1, Q3] | 9 [7, 11] | 9 [7, 12] | 9 [7, 12] |
| Missing | 0 (0%) | 0 (0%) | 0 (0%) |
| **CO (L/min)** | | | |
| Median [Q1, Q3] | 3.8 [3, 4.8] | 4.4 [3.4, 5.4] | 4 [3.1, 5] |
| Missing | 0 (0%) | 0 (0%) | 0 (0%) |
| **CI (L/(min·m²))** | | | |
| Median [Q1, Q3] | 2.2 [1.7, 2.7] | 2.2 [1.8, 2.7] | 2.2 [1.7, 2.7] |
| Missing | 38 (2.5%) | 11 (1.9%) | 49 (2.3%) |
| **PVR (WU)** | | | |
| Median [Q1, Q3] | 9.9 [6.1, 15] | 8.4 [6.1, 12] | 9.5 [6.1, 14] |
| Missing | 0 (0%) | 0 (0%) | 0 (0%) |

**Table E12: Baseline characteristics stratified by race and sex in PH patients.**

Baseline characteristics are shown for patients with reported race.

| **Sex** | **female** | | | **male** | | | **Overall** |
| --- | --- | --- | --- | --- | --- | --- | --- |
| **Race** | **White** | **Black** | **Asian** | **White** | **Black** | **Asian** |  |
| N | 6246 | 1074 | 382 | 5633 | 666 | 228 | 14229 |
| **Age at diagnosis (years)** | | | | | | | |
| Median [Q1, Q3] | 64 [52, 73] | 55 [45, 65] | 56 [42, 69] | 64 [55, 72] | 54 [46, 64] | 59 [47, 66] | 63 [52, 71] |
| Missing | 0 (0%) | 0 (0%) | 0 (0%) | 0 (0%) | 0 (0%) | 0 (0%) | 0 (0%) |
| **WHO FC** | | | | | | | |
| I | 109 (3.1%) | 28 (5%) | 11 (3.3%) | 159 (5%) | 33 (8.4%) | 16 (7.7%) | 356 (4.3%) |
| II | 737 (21%) | 136 (24%) | 64 (19%) | 695 (22%) | 90 (23%) | 71 (34%) | 1793 (22%) |
| III | 2203 (62%) | 331 (59%) | 211 (64%) | 1885 (59%) | 211 (54%) | 101 (49%) | 4942 (60%) |
| IV | 480 (14%) | 62 (11%) | 43 (13%) | 456 (14%) | 58 (15%) | 20 (9.6%) | 1119 (14%) |
| Missing | 2717 (43%) | 517 (48%) | 53 (14%) | 2438 (43%) | 274 (41%) | 20 (8.8%) | 6019 (42%) |
| **BMI (kg/m²)** | | | | | | | |
| Median [Q1, Q3] | 28 [24, 35] | 31 [25, 37] | 24 [21, 29] | 28 [25, 33] | 29 [24, 33] | 25 [22, 28] | 28 [24, 34] |
| Missing | 379 (6.1%) | 109 (10%) | 49 (13%) | 257 (4.6%) | 47 (7.1%) | 10 (4.4%) | 851 (6%) |
| **BSA (m²)** | | | | | | | |
| Median [Q1, Q3] | 1.8 [1.7, 2] | 1.9 [1.7, 2.1] | 1.6 [1.5, 1.7] | 2.1 [1.9, 2.2] | 2.1 [1.9, 2.3] | 1.8 [1.7, 2] | 1.9 [1.7, 2.1] |
| Missing | 137 (2.2%) | 19 (1.8%) | 24 (6.3%) | 85 (1.5%) | 9 (1.4%) | 6 (2.6%) | 280 (2%) |
| **Height (cm)** | | | | | | | |
| Median [Q1, Q3] | 160 [160, 170] | 160 [160, 170] | 160 [150, 160] | 180 [170, 180] | 180 [170, 190] | 170 [160, 180] | 170 [160, 180] |
| Missing | 476 (7.6%) | 99 (9.2%) | 44 (12%) | 319 (5.7%) | 50 (7.5%) | 9 (3.9%) | 997 (7%) |
| **Weight (kg)** | | | | | | | |
| Median [Q1, Q3] | 75 [63, 91] | 81 [68, 99] | 60 [52, 71] | 88 [77, 100] | 90 [77, 110] | 73 [62, 82] | 82 [68, 98] |
| Missing | 522 (8.4%) | 108 (10%) | 55 (14%) | 333 (5.9%) | 49 (7.4%) | 13 (5.7%) | 1080 (7.6%) |
| **6MWD (m/6min)** | | | | | | | |
| Median [Q1, Q3] | 290 [180, 370] | 250 [160, 340] | 290 [180, 370] | 300 [200, 390] | 320 [220, 430] | 380 [220, 450] | 290 [190, 380] |
| Missing | 3894 (62%) | 809 (75%) | 109 (29%) | 4055 (72%) | 586 (88%) | 65 (29%) | 9518 (67%) |
| **BNP (pg/mL)** | | | | | | | |
| Median [Q1, Q3] | 240 [91, 600] | 390 [120, 1100] | 160 [43, 480] | 340 [130, 820] | 700 [230, 1600] | 160 [53, 400] | 290 [100, 760] |
| Missing | 2451 (39%) | 401 (37%) | 82 (21%) | 2109 (37%) | 203 (30%) | 24 (11%) | 5270 (37%) |
| **mPAP (mmHg)** | | | | | | | |
| Median [Q1, Q3] | 36 [28, 47] | 38 [30, 47] | 43 [33, 51] | 34 [27, 44] | 35 [28, 44] | 39 [30, 46] | 36 [28, 46] |
| Missing | 0 (0%) | 0 (0%) | 0 (0%) | 0 (0%) | 0 (0%) | 0 (0%) | 0 (0%) |
| **sPAP (mmHg)** | | | | | | | |
| Median [Q1, Q3] | 58 [43, 76] | 58 [45, 75] | 70 [55, 84] | 52 [40, 70] | 53 [42, 67] | 66 [50, 81] | 56 [42, 73] |
| Missing | 117 (1.9%) | 4 (0.37%) | 1 (0.26%) | 108 (1.9%) | 1 (0.15%) | 0 (0%) | 231 (1.6%) |
| **CVP (mmHg)** | | | | | | | |
| Median [Q1, Q3] | 8 [5, 12] | 10 [5, 14] | 7 [4, 11] | 8 [4, 12] | 10 [6, 13] | 6 [3, 8] | 8 [5, 12] |
| Missing | 2782 (45%) | 702 (65%) | 81 (21%) | 3280 (58%) | 551 (83%) | 36 (16%) | 7432 (52%) |
| **PAWP (mmHg)** | | | | | | | |
| Median [Q1, Q3] | 13 [9, 19] | 15 [10, 21] | 10 [7, 14] | 15 [10, 21] | 17 [13, 24] | 10 [8, 14] | 14 [10, 20] |
| Missing | 0 (0%) | 0 (0%) | 0 (0%) | 0 (0%) | 0 (0%) | 0 (0%) | 0 (0%) |
| **CO (L/min)** | | | | | | | |
| Median [Q1, Q3] | 4.4 [3.5, 5.5] | 4.5 [3.6, 5.7] | 3.4 [2.7, 4.3] | 5 [4, 6.1] | 4.9 [3.9, 6.2] | 3.9 [2.9, 5.1] | 4.6 [3.7, 5.8] |
| Missing | 0 (0%) | 0 (0%) | 0 (0%) | 0 (0%) | 0 (0%) | 0 (0%) | 0 (0%) |
| **CI (L/(min·m²))** | | | | | | | |
| Median [Q1, Q3] | 2.4 [2, 3] | 2.4 [1.9, 3] | 2.1 [1.7, 2.6] | 2.4 [2, 2.9] | 2.3 [1.9, 2.9] | 2.2 [1.7, 2.6] | 2.4 [1.9, 3] |
| Missing | 131 (2.1%) | 19 (1.8%) | 24 (6.3%) | 76 (1.3%) | 8 (1.2%) | 6 (2.6%) | 264 (1.9%) |
| **PVR (WU)** | | | | | | | |
| Median [Q1, Q3] | 4.5 [2.5, 8.6] | 4.2 [2.6, 7.8] | 9.1 [5.6, 13] | 3.2 [1.9, 5.8] | 3.2 [2, 5.1] | 7.1 [4.1, 11] | 3.9 [2.2, 7.4] |
| Missing | 0 (0%) | 0 (0%) | 0 (0%) | 0 (0%) | 0 (0%) | 0 (0%) | 0 (0%) |
