# Supplementary material for: Male survival disadvantage in pulmonary hypertension: independent of aetiology, age, disease severity, comorbidities and treatment
Source: eBioMedicine. 2025 Dec 16;123:106063. doi: 10.1016/j.ebiom.2025.106063 (PMC12768861; doi:10.1016/j.ebiom.2025.106063)
Supplement: Consortium Supplement [file mmc9.docx]

**The PVRI Consortium further consists of:**

| **First names** | **Surnames** |
| --- | --- |
| James¹ | Anderson |
| Tobiah²˒³ | Antoine |
| Felix⁴ | Ballmann |
| Harm Jan⁵ | Bogaard |
| Victoria⁶ | Damonte |
| Nathan⁷ | Dwyer |
| Diego⁸ | Echazarreta |
| Kai⁹˒²² | Förster |
| Lars¹⁰ | Harbaum |
| Melanie¹¹ | Heberling |
| Anne⁹˒²² | Hilgendorff |
| Ernesto⁶ | Junaeda |
| Ingrid¹² | King |
| Hans¹⁰ | Klose |
| Philipp²˒³ | Krieb |
| Edmund¹³ | Lau |
| Melanie¹⁴ | Lavender |
| Kurt² | Marquardt |
| Mauricio¹⁵ | Orozco-Levi |
| Karen¹⁶ | Osborn |
| Stephan⁴˒¹⁷ | Rosenkranz |
| Siva¹⁸ | Sivakumaran |
| Ioan¹⁹ | Tilea |
| Andrea¹⁹ | Varga |
| Helen M.²⁰˒²¹˒²² | Whitford |
| Christoph B.²³ | Wiedenroth |

^1^Sunshine Coast University Hospital, ^2^Department of Internal Medicine, Universities of Giessen and Marburg Lung Center (UGMLC), Member of the German Center for Lung Research (DZL), Giessen (Germany), ^3^Institute for Lung Health (ILH), Cardio-Pulmonary Institute (CPI), Giessen (Germany), ^4^Clinic III for Internal Medicine (Department of Cardiology), Heart Center at the University Hospital Cologne, Kerpener Str. 62, D-50937 Cologne, Germany, ^5^Amsterdam UMC, ^6^University of Cordoba (Argentina), ^7^Royal Hobart Hospital, ^8^Universidad Nacional de La Plata (Argentina), ^9^Ludwig-Maximillians-University Munich (Germany), ^10^Universitatsklinikum Hamburg-Eppendorf, ^11^Division of Pulmonology, Medical Department I, University Hospital Carl Gustav Carus of Technical University Dresden, Dresden, Germany, ^12^Murdoch Children’s Research Institute, University of Melbourne, Melbourne, VIC, Australia , ^13^Royal Prince Alfred Hospital , ^14^Fiona Stanley Hospital , ^15^Fundacion Cardiovascular de Colombia, ^16^Pulmonary Vascular Research Institute (PVRI) Canterbury (UK), ^17^Cologne Cardiovascular Research Center (CCRC), Hospital Cologne and Medical Faculty, Heart Center at the University, University of Cologne, Cologne, Germany, ^18^Gold Coast University Hospital, ^19^George Emil Palade University of Medicine (Romania), ^20^The Royal Children’s Hospital, ^21^Department of Respiratory Medicine, The Alfred Hospital, Melbourne, VIC, Australia, ^22^Faculty of Medicine, Nursing and Health Sciences, Central Clinical School, Monash University, Melbourne, VIC, Australia, ^23^Kerckhoff Klinik and German Centre for Lung Research (DZL/UGMLC)
